# Supplementary material for: The pro-regenerative effects of hyperIL6 in drug-induced liver injury are unexpectedly due to competitive inhibition of IL11 signaling
Source: eLife. 2021 Aug 26;10:e68843. doi: 10.7554/eLife.68843 (PMC8445623; doi:10.7554/eLife.68843)
Supplement: Figure 1—source data 2. [file elife-68843-fig1-data2.zip › Figure 1-Uncropped WB images with markers.pptx]

## Slide 1
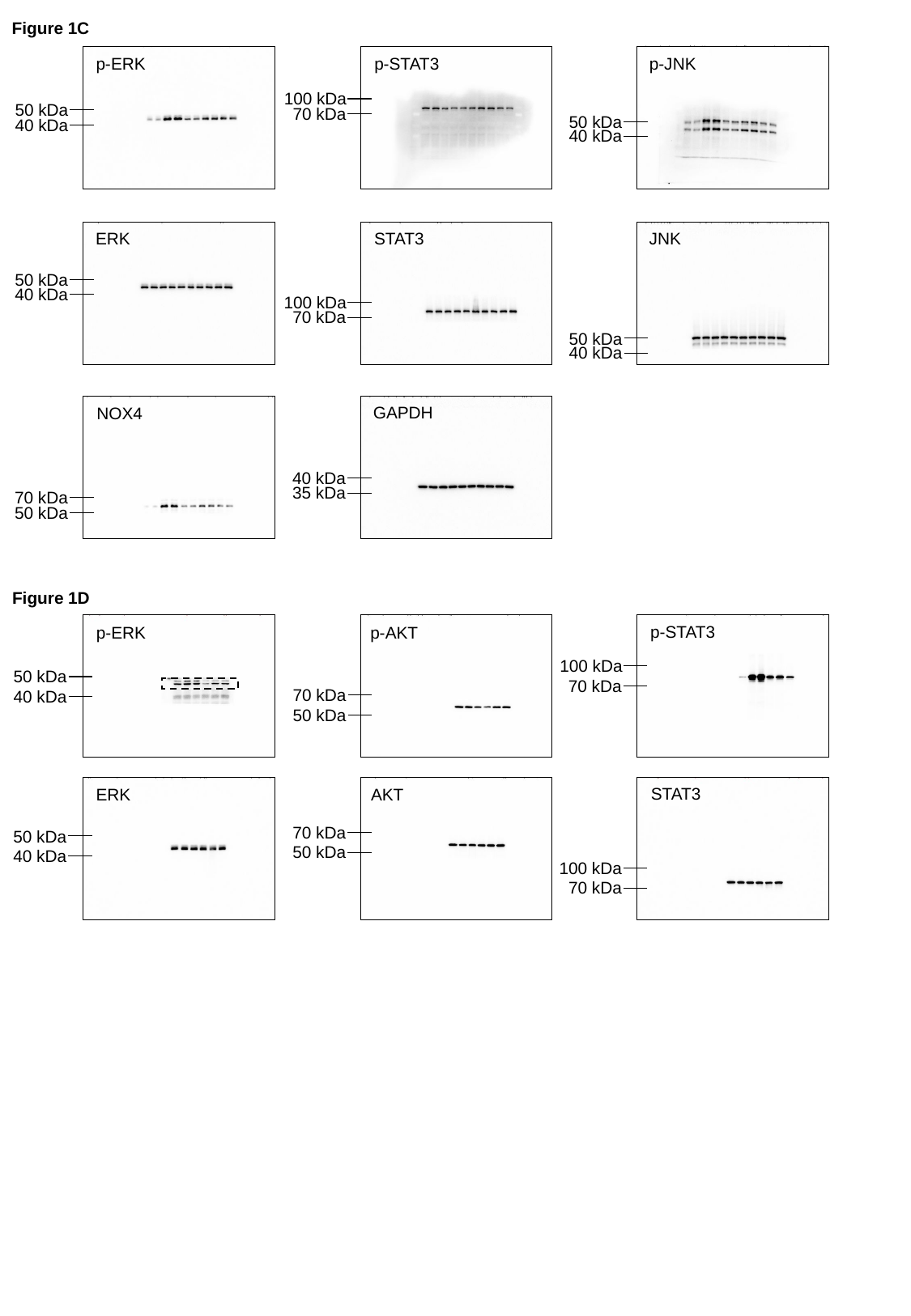

Figure 1C
p-JNK
p-ERK
p-STAT3
100 kDa
70 kDa
50 kDa
40 kDa
50 kDa
40 kDa
JNK
ERK
STAT3
50 kDa
40 kDa
100 kDa
70 kDa
50 kDa
40 kDa
GAPDH
NOX4
40 kDa
35 kDa
70 kDa
50 kDa
Figure 1D
p-STAT3
p-ERK
p-AKT
100 kDa
70 kDa
50 kDa
40 kDa
70 kDa
50 kDa
STAT3
ERK
AKT
70 kDa
50 kDa
50 kDa
40 kDa
100 kDa
70 kDa

## Slide 2
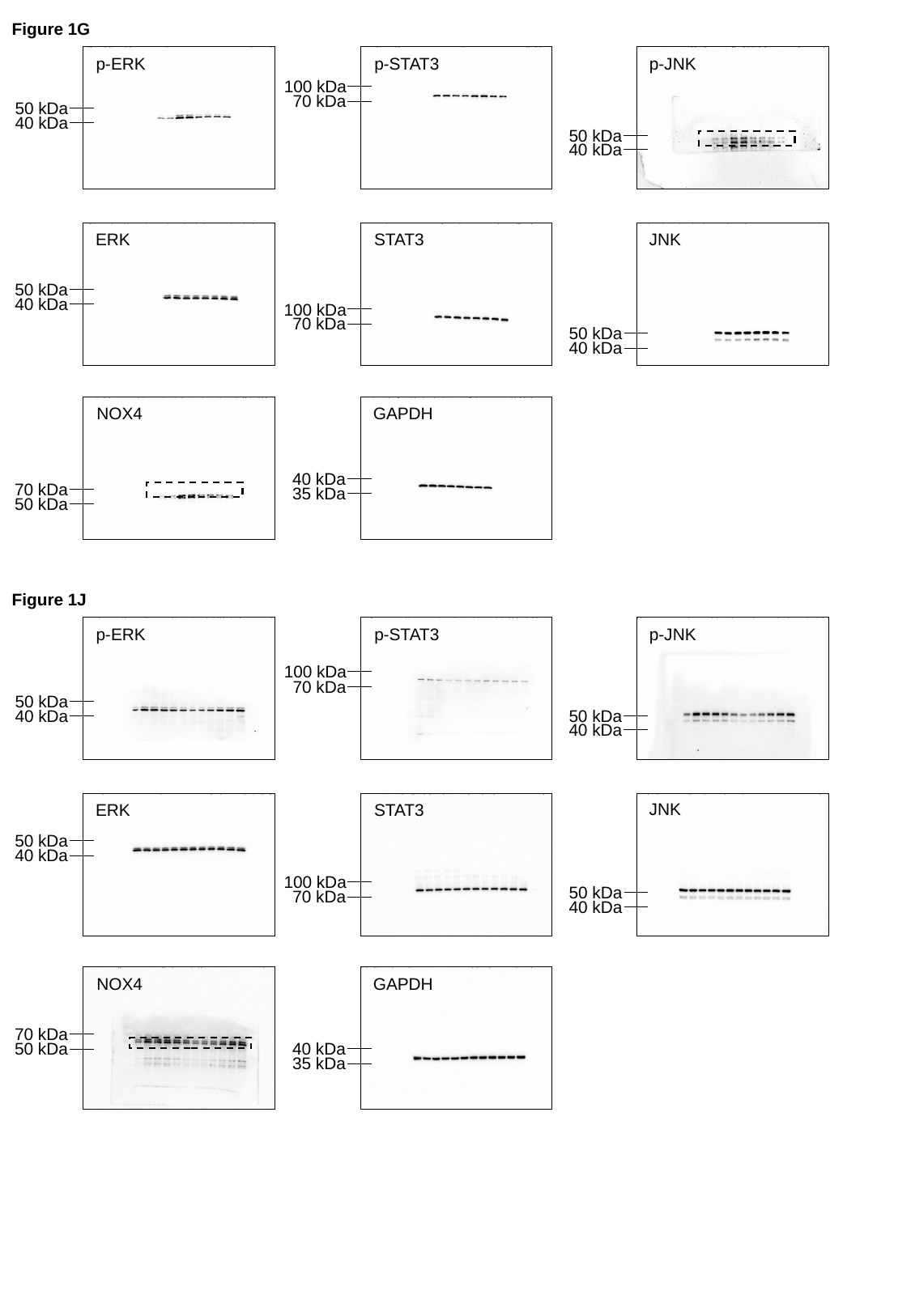

Figure 1G
p-JNK
p-ERK
p-STAT3
100 kDa
70 kDa
50 kDa
40 kDa
50 kDa
40 kDa
JNK
ERK
STAT3
50 kDa
40 kDa
100 kDa
70 kDa
50 kDa
40 kDa
GAPDH
NOX4
40 kDa
35 kDa
70 kDa
50 kDa
Figure 1J
p-JNK
p-ERK
p-STAT3
100 kDa
70 kDa
50 kDa
40 kDa
50 kDa
40 kDa
JNK
ERK
STAT3
50 kDa
40 kDa
100 kDa
70 kDa
50 kDa
40 kDa
GAPDH
NOX4
70 kDa
50 kDa
40 kDa
35 kDa
