# Supplementary figures and images for: The pro-regenerative effects of hyperIL6 in drug-induced liver injury are unexpectedly due to competitive inhibition of IL11 signaling
Source: eLife. 2021 Aug 26;10:e68843. doi: 10.7554/eLife.68843 (PMC8445623; doi:10.7554/eLife.68843)

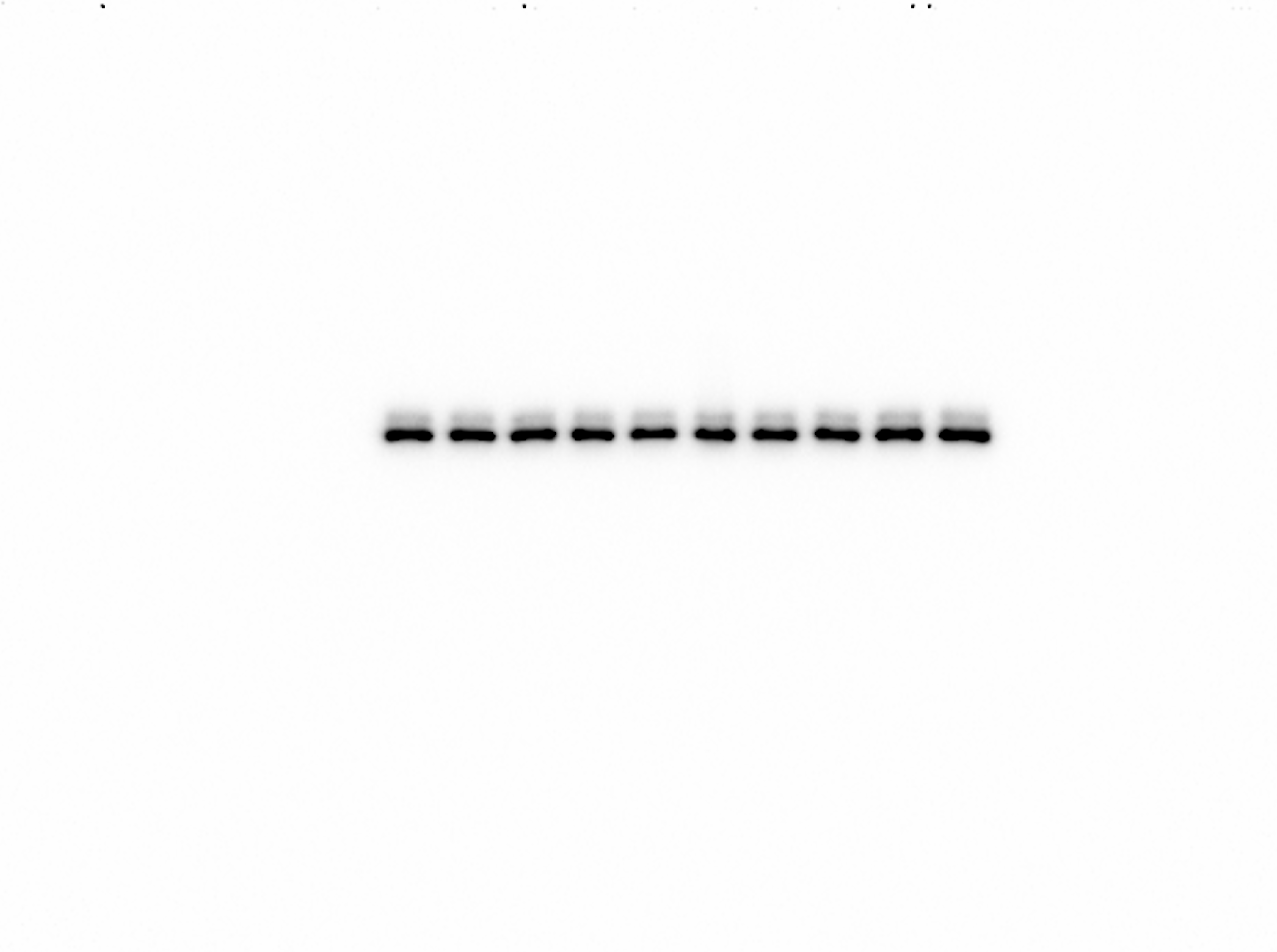

Supplement: Figure 1—source data 2. [file elife-68843-fig1-data2.zip › Figure 1C-Original WB images/Fig.1C ERK.tif]

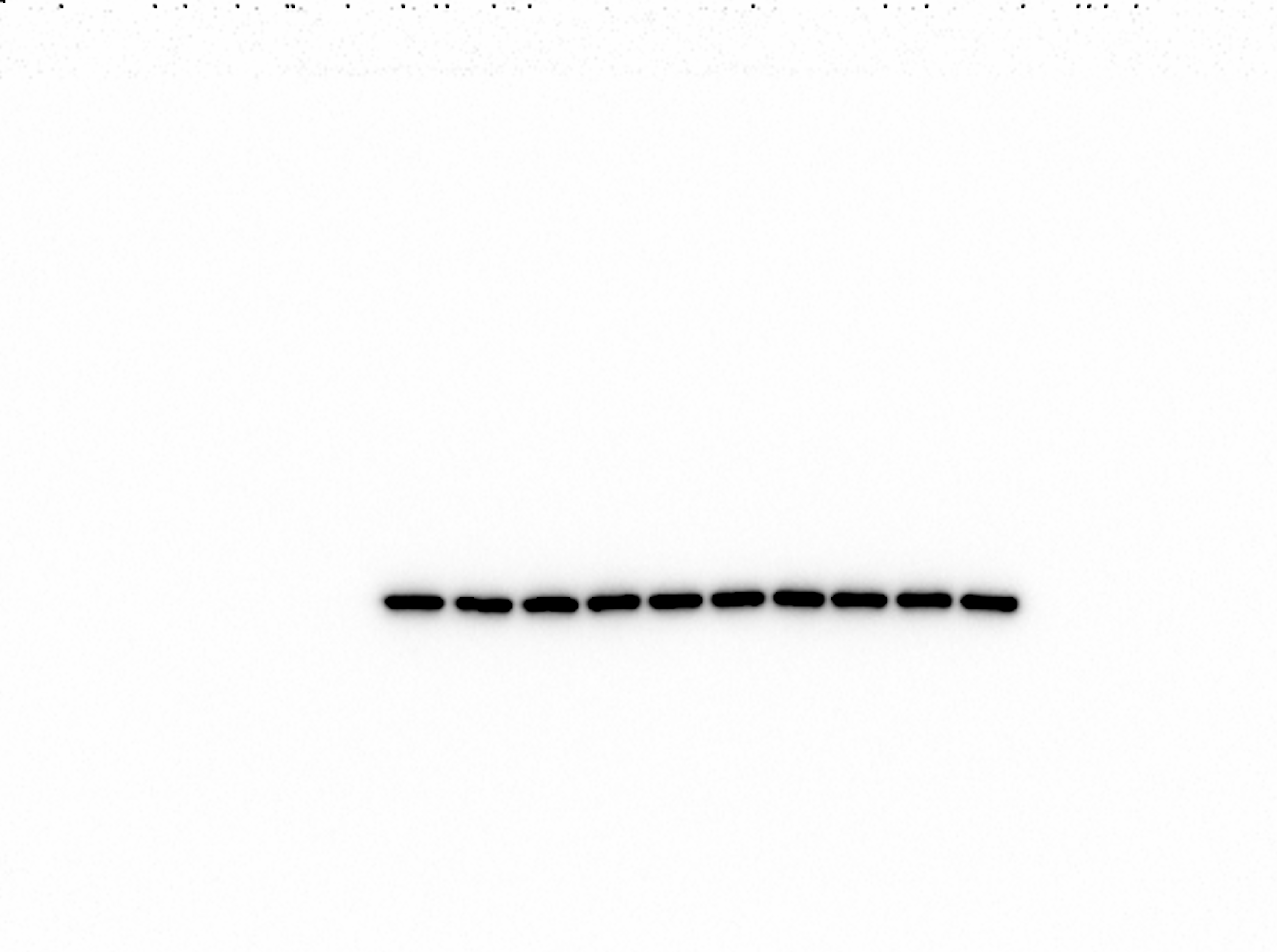

Supplement: Figure 1—source data 2. [file elife-68843-fig1-data2.zip › Figure 1C-Original WB images/Fig.1C GAPDH.tif]

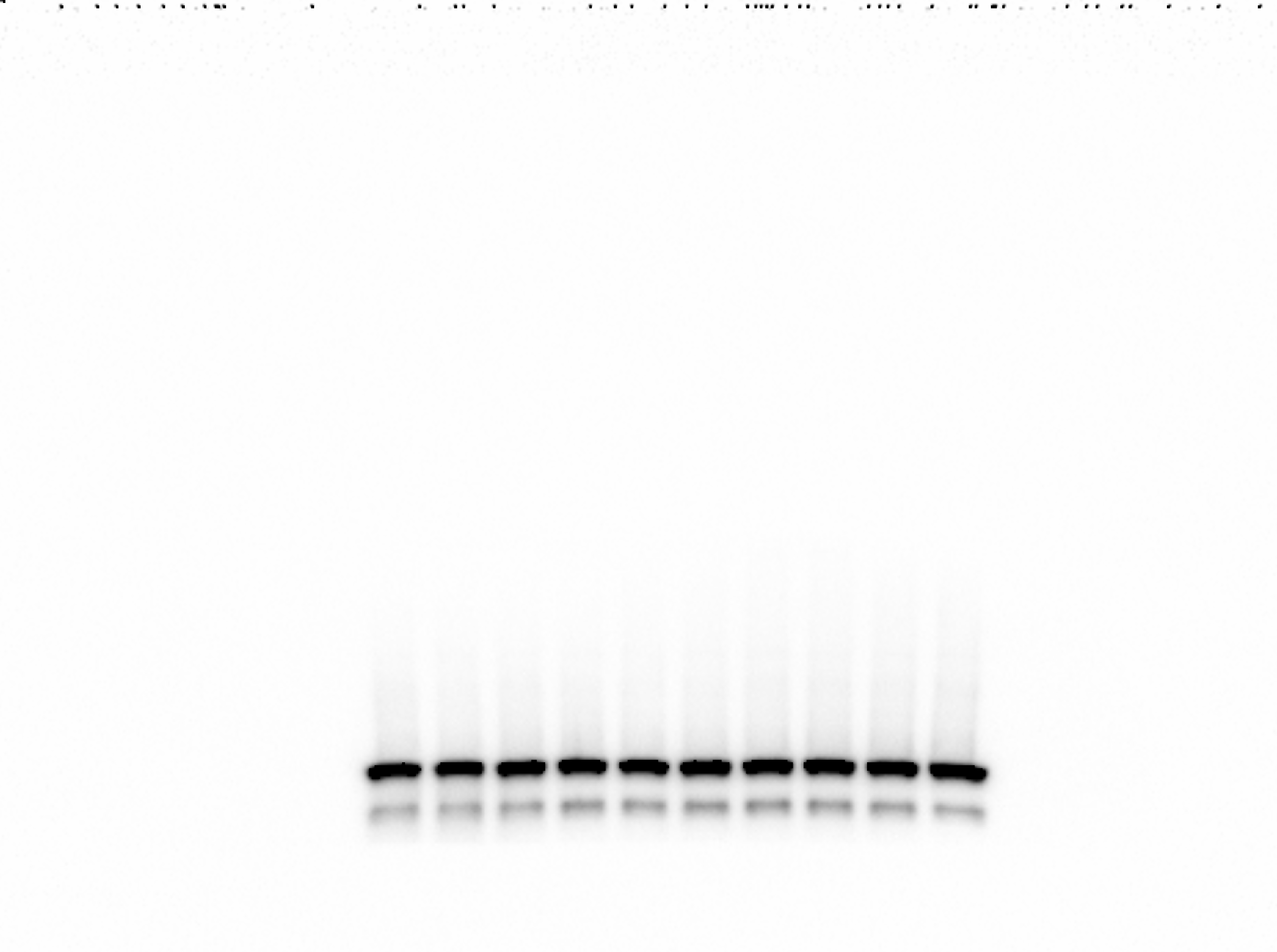

Supplement: Figure 1—source data 2. [file elife-68843-fig1-data2.zip › Figure 1C-Original WB images/Fig.1C JNK.tif]

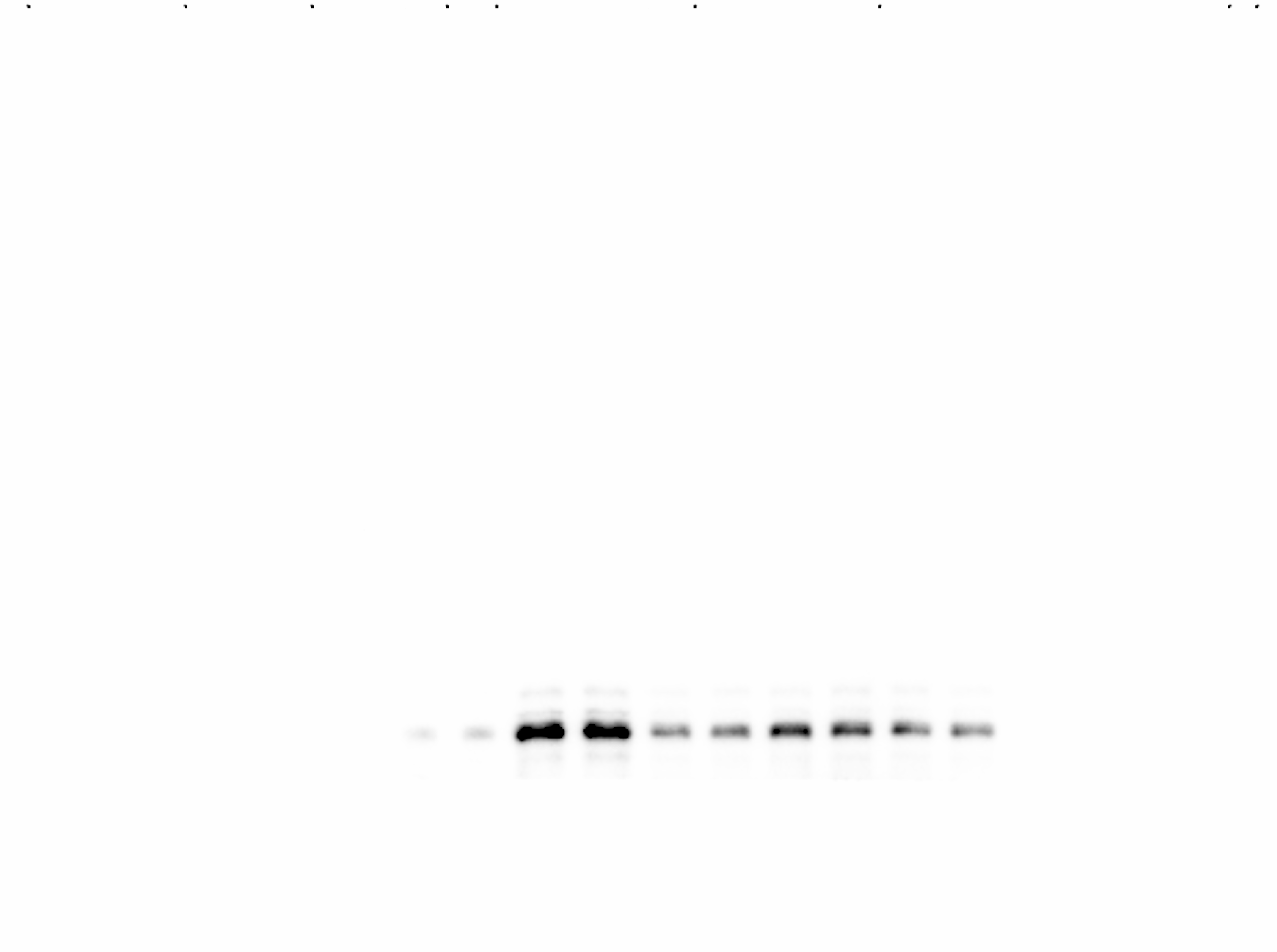

Supplement: Figure 1—source data 2. [file elife-68843-fig1-data2.zip › Figure 1C-Original WB images/Fig.1C NOX4.tif]

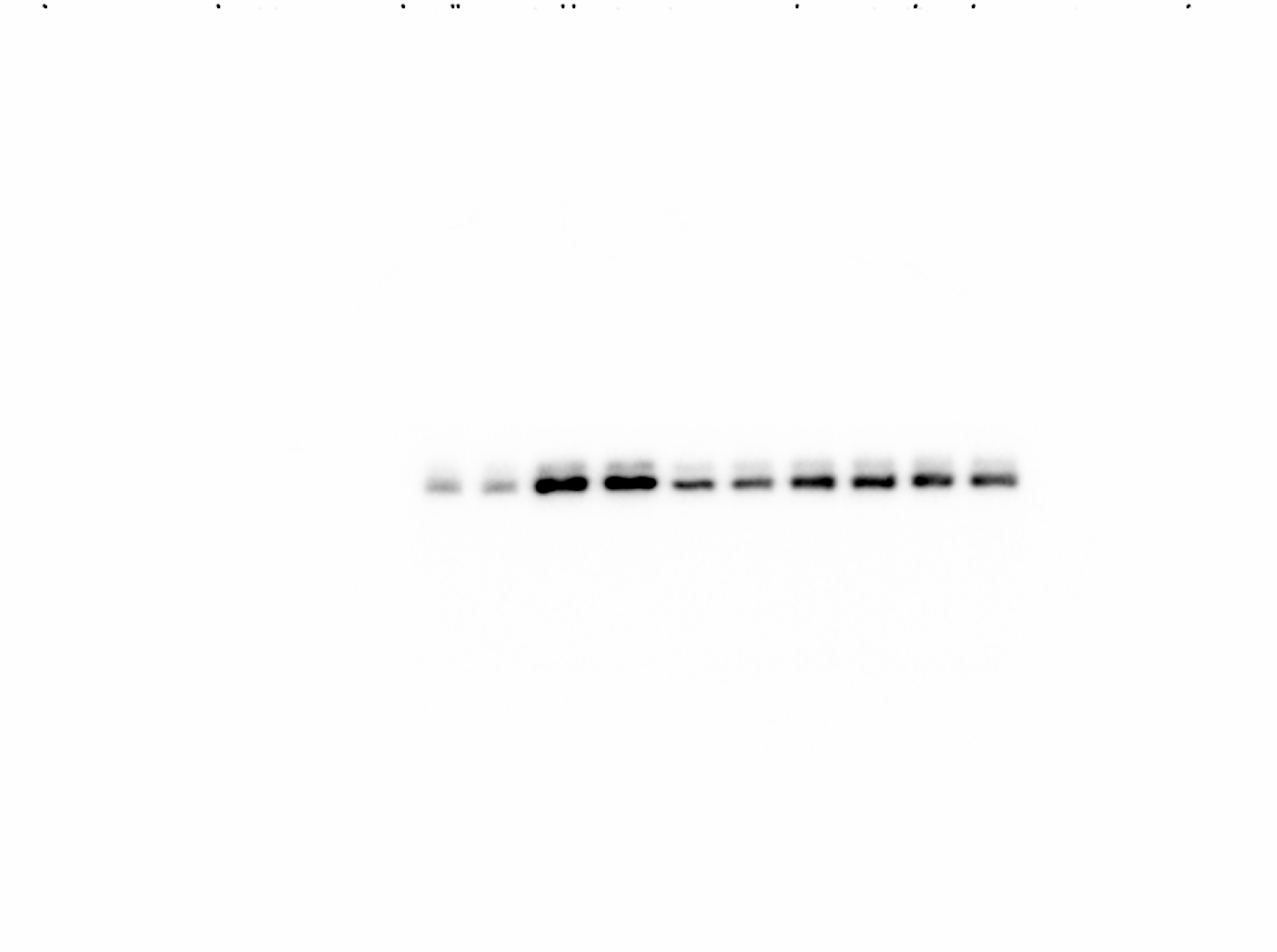

Supplement: Figure 1—source data 2. [file elife-68843-fig1-data2.zip › Figure 1C-Original WB images/Fig.1C p-ERK.tif]

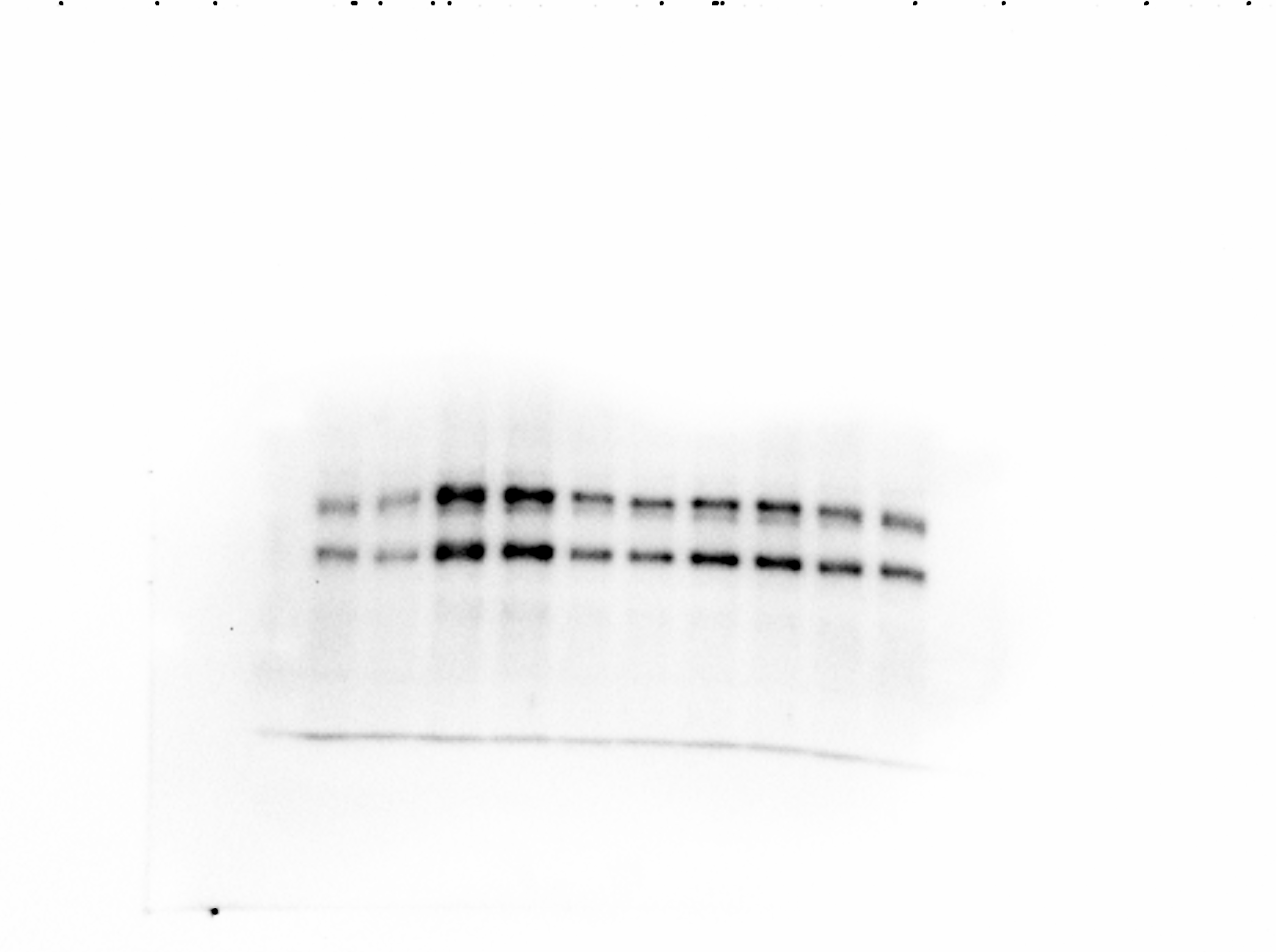

Supplement: Figure 1—source data 2. [file elife-68843-fig1-data2.zip › Figure 1C-Original WB images/Fig.1C p-JNK.tif]

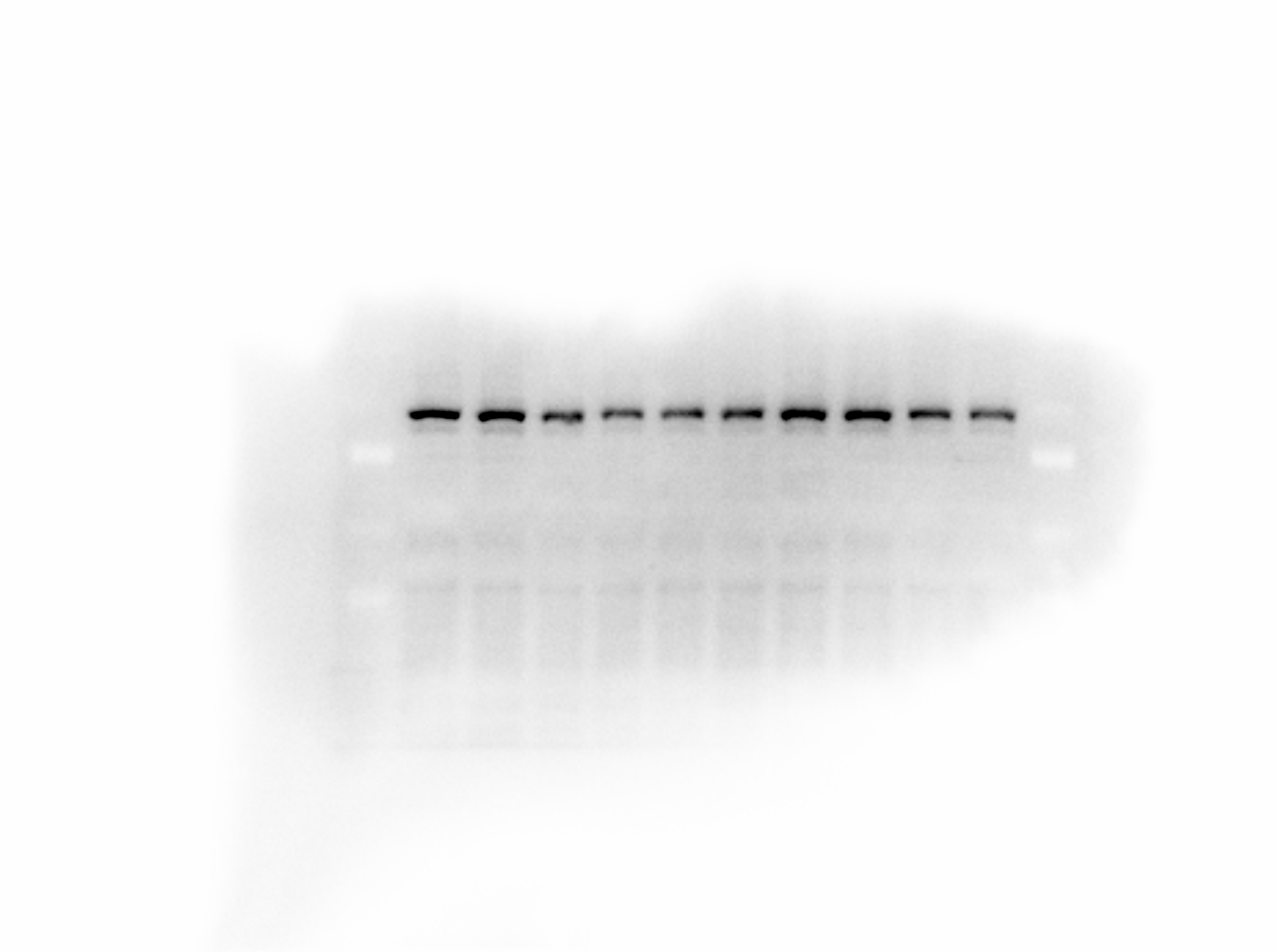

Supplement: Figure 1—source data 2. [file elife-68843-fig1-data2.zip › Figure 1C-Original WB images/Fig.1C p-STAT3.tif]

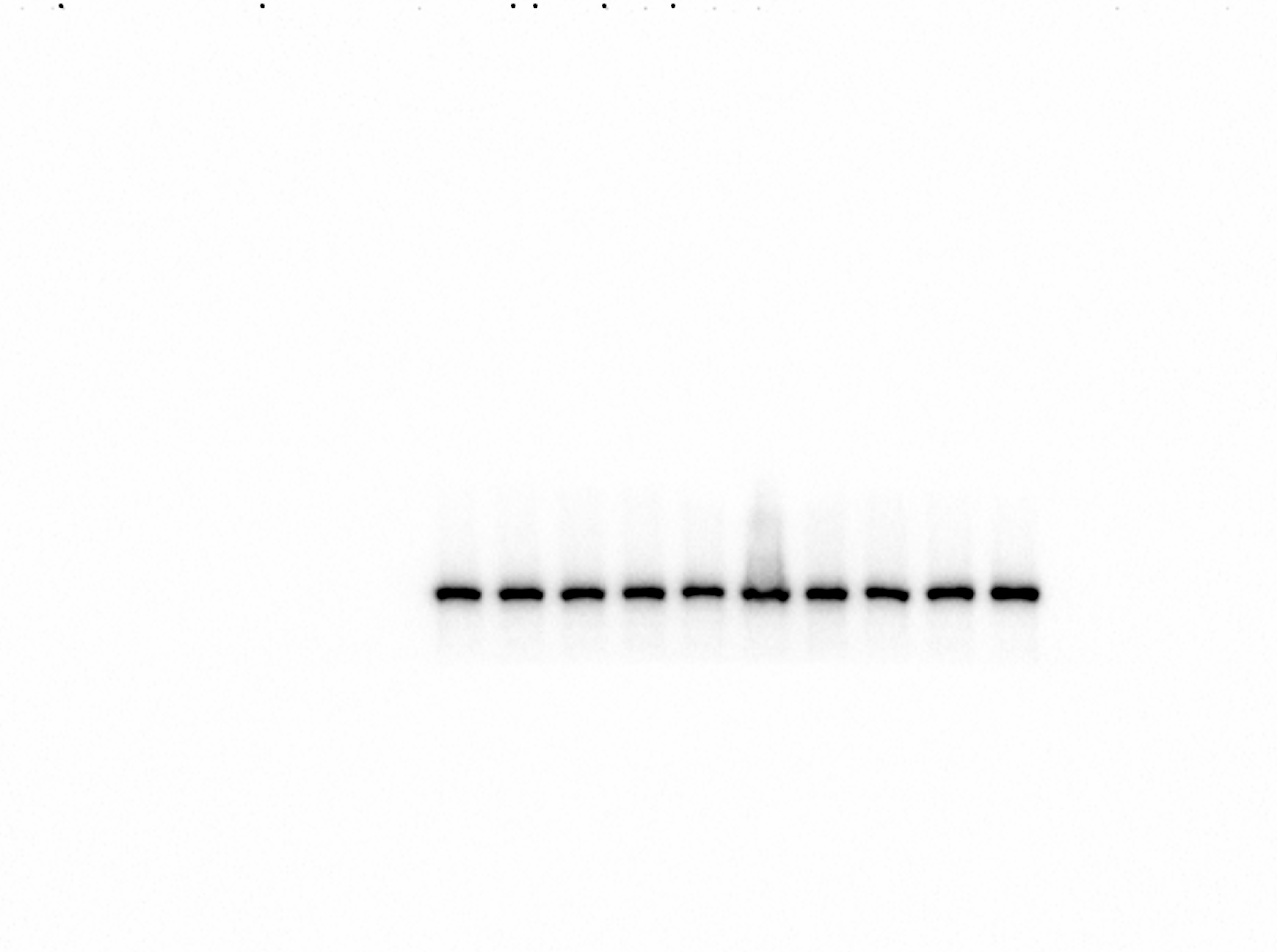

Supplement: Figure 1—source data 2. [file elife-68843-fig1-data2.zip › Figure 1C-Original WB images/Fig.1C STAT3.tif]

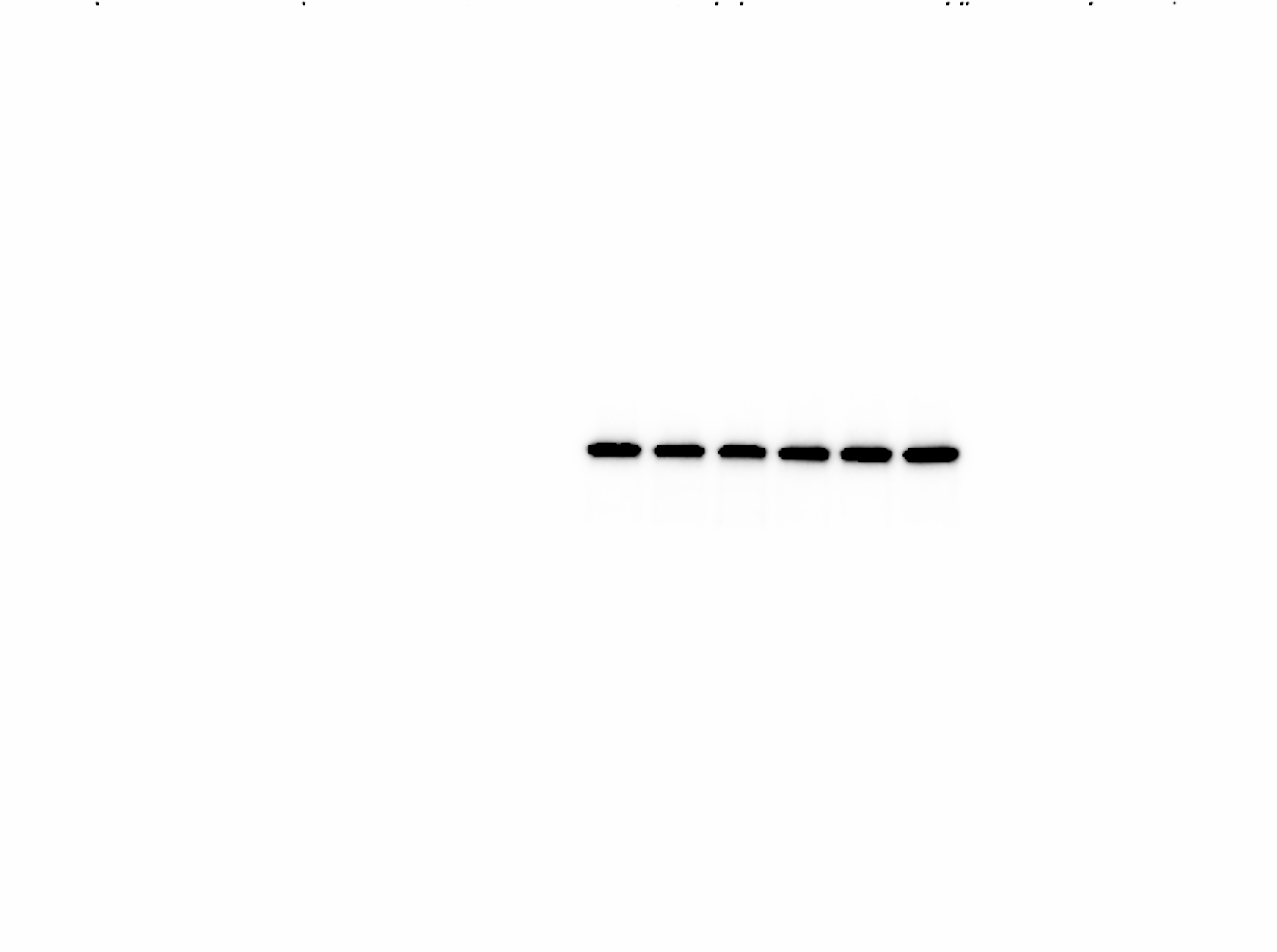

Supplement: Figure 1—source data 2. [file elife-68843-fig1-data2.zip › Figure 1D-Original WB images/Fig.1D AKT.tif]

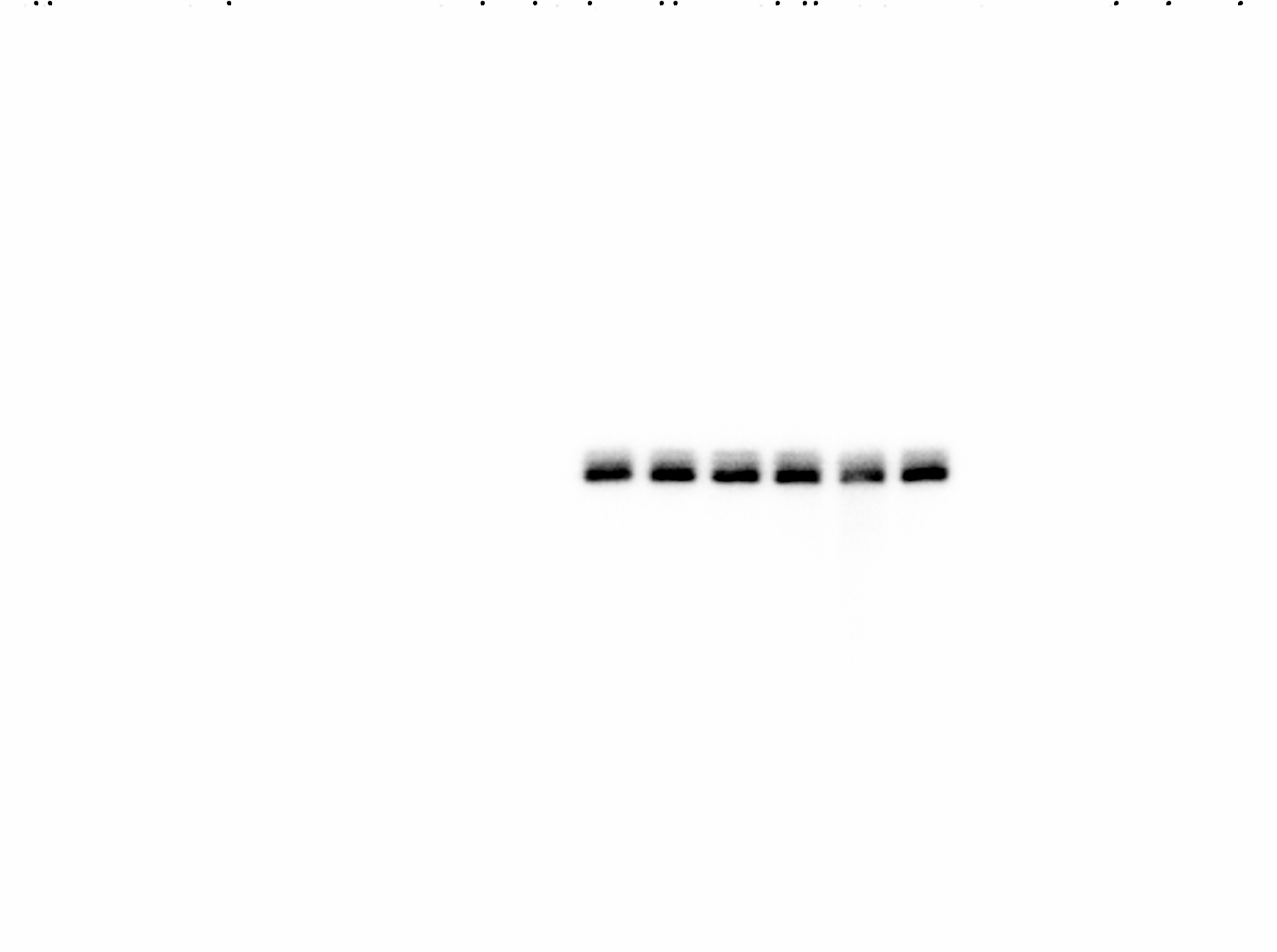

Supplement: Figure 1—source data 2. [file elife-68843-fig1-data2.zip › Figure 1D-Original WB images/Fig.1D ERK.tif]

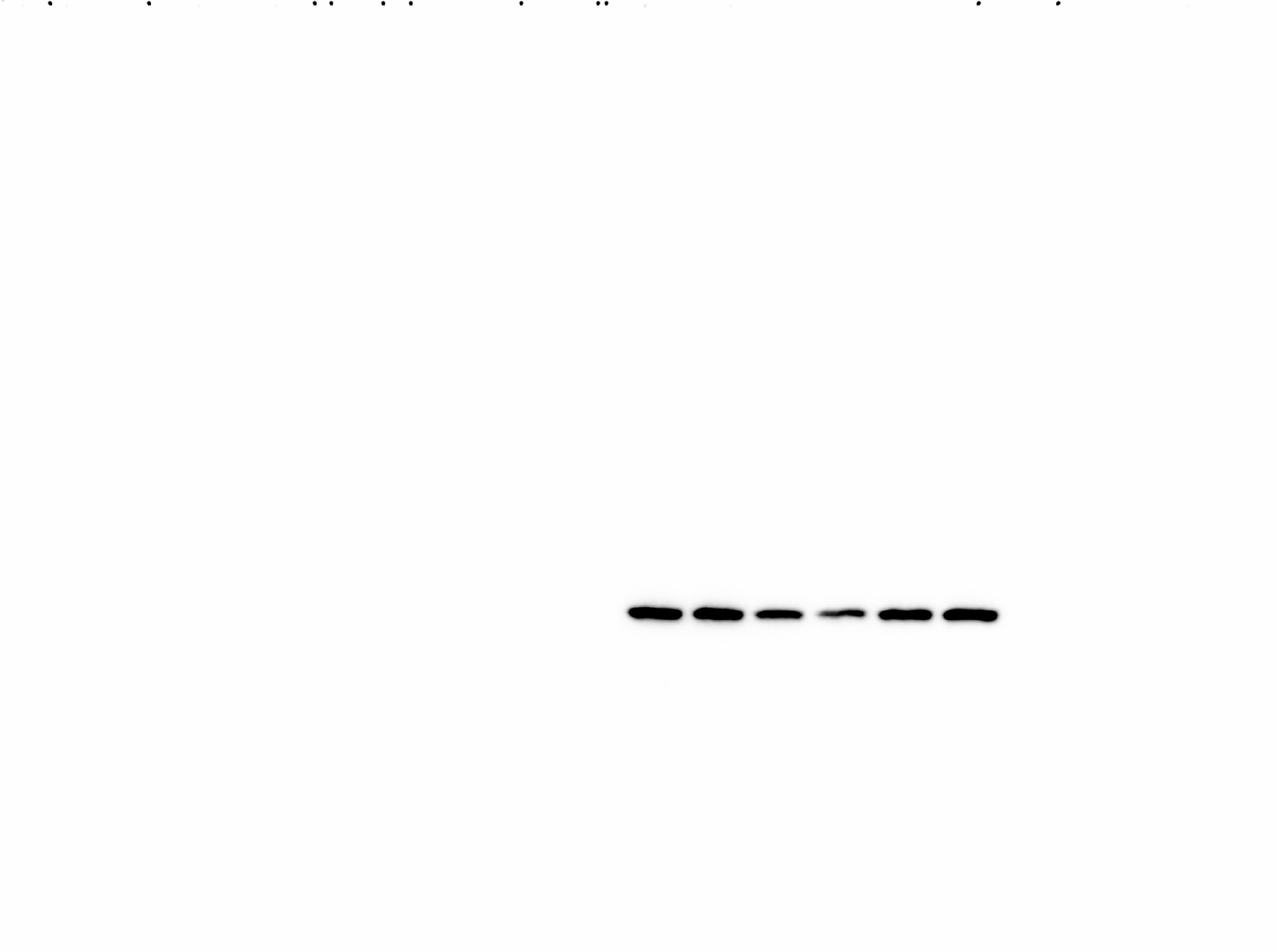

Supplement: Figure 1—source data 2. [file elife-68843-fig1-data2.zip › Figure 1D-Original WB images/Fig.1D p-AKT.tif]

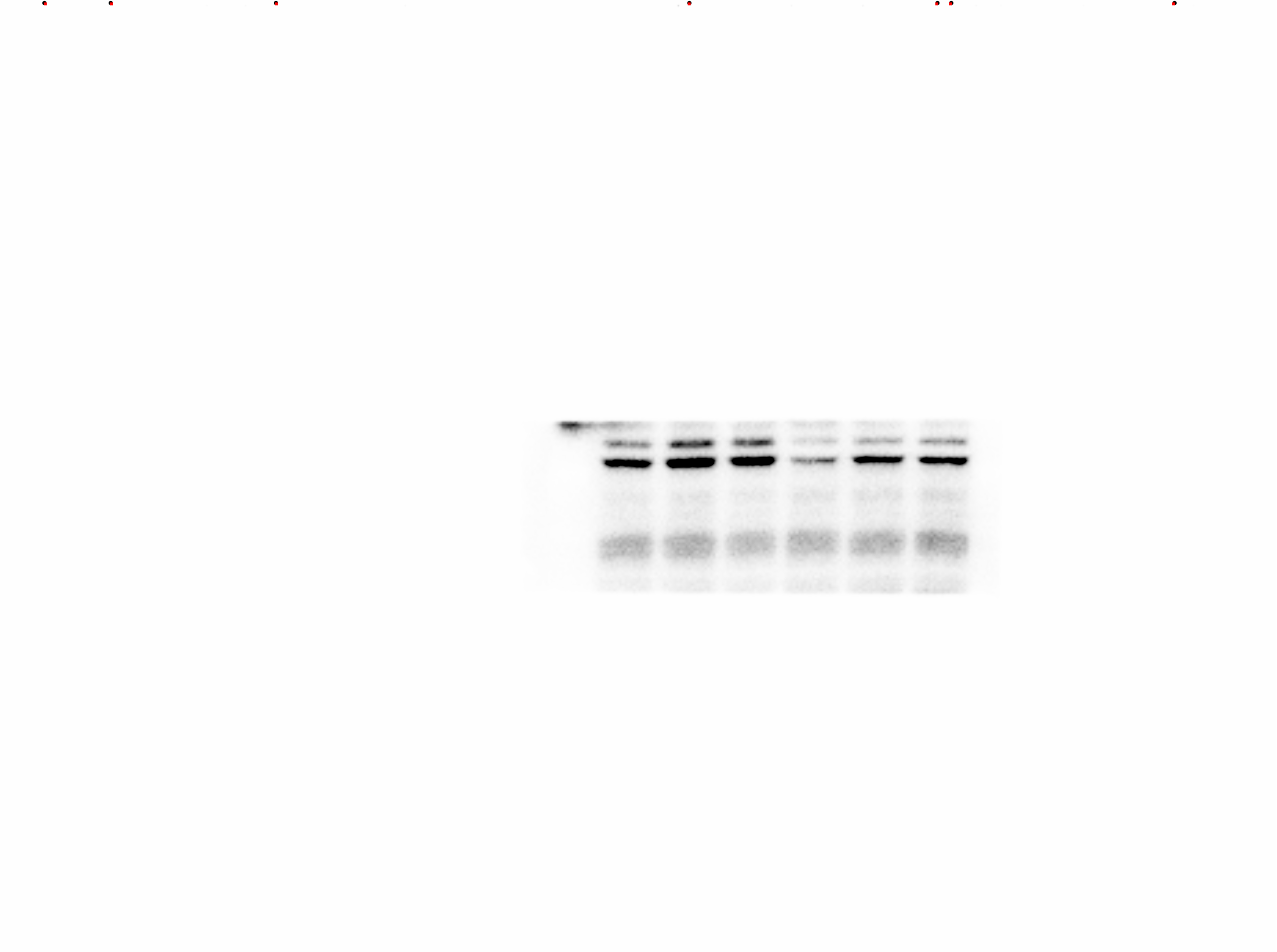

Supplement: Figure 1—source data 2. [file elife-68843-fig1-data2.zip › Figure 1D-Original WB images/Fig.1D p-ERK.tif]

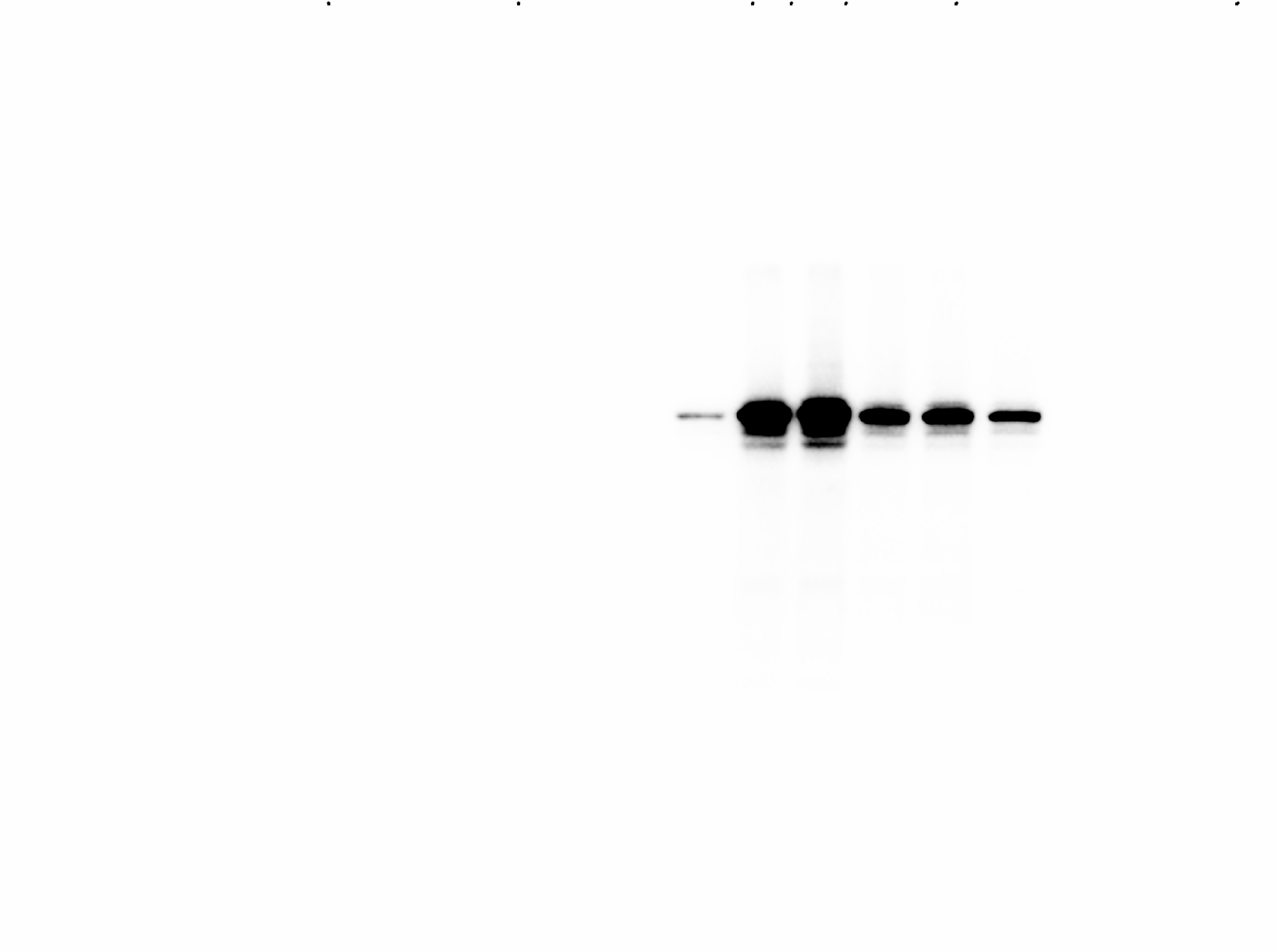

Supplement: Figure 1—source data 2. [file elife-68843-fig1-data2.zip › Figure 1D-Original WB images/Fig.1D p-STAT3.tif]

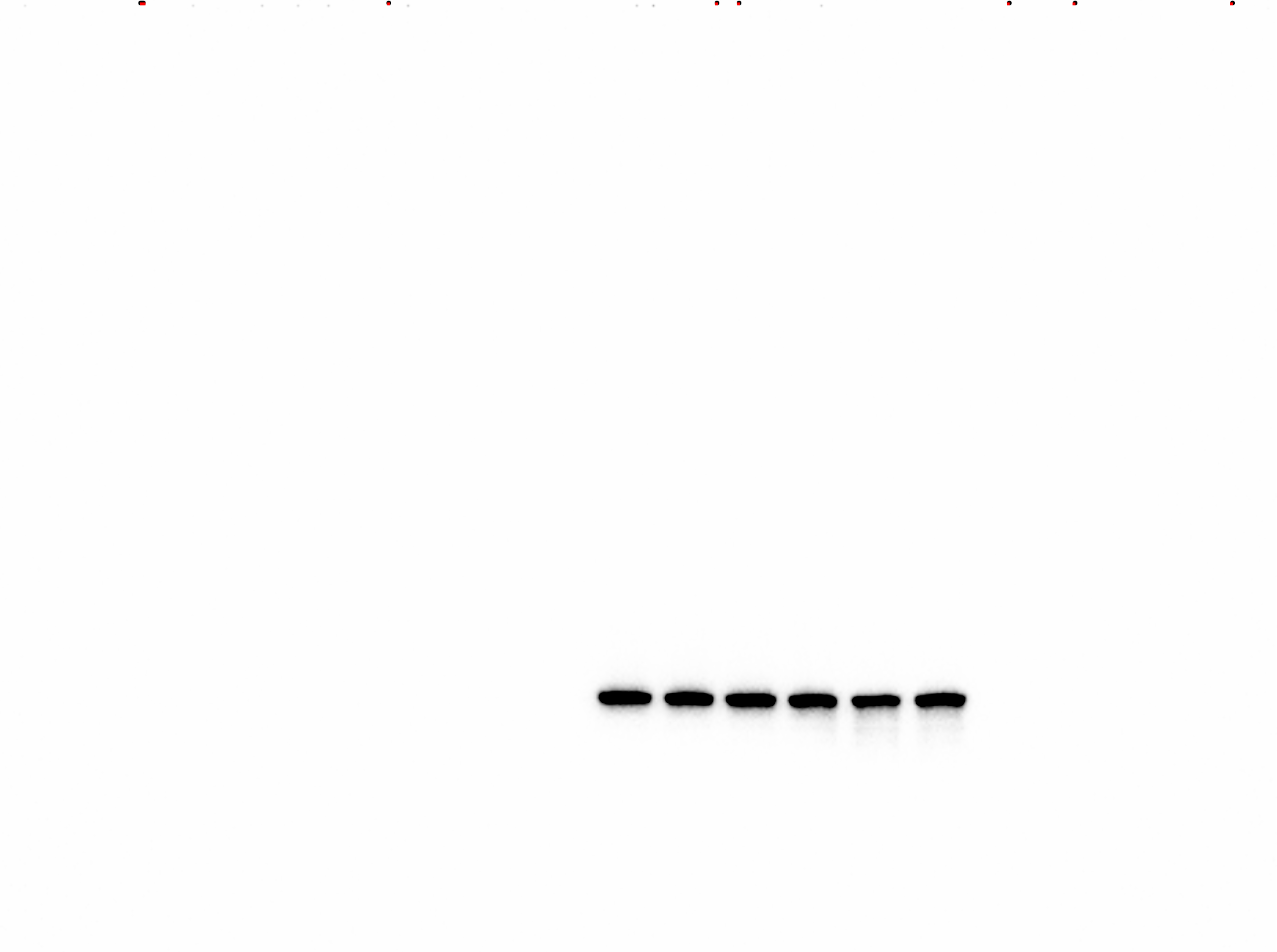

Supplement: Figure 1—source data 2. [file elife-68843-fig1-data2.zip › Figure 1D-Original WB images/Fig.1D STAT3.tif]

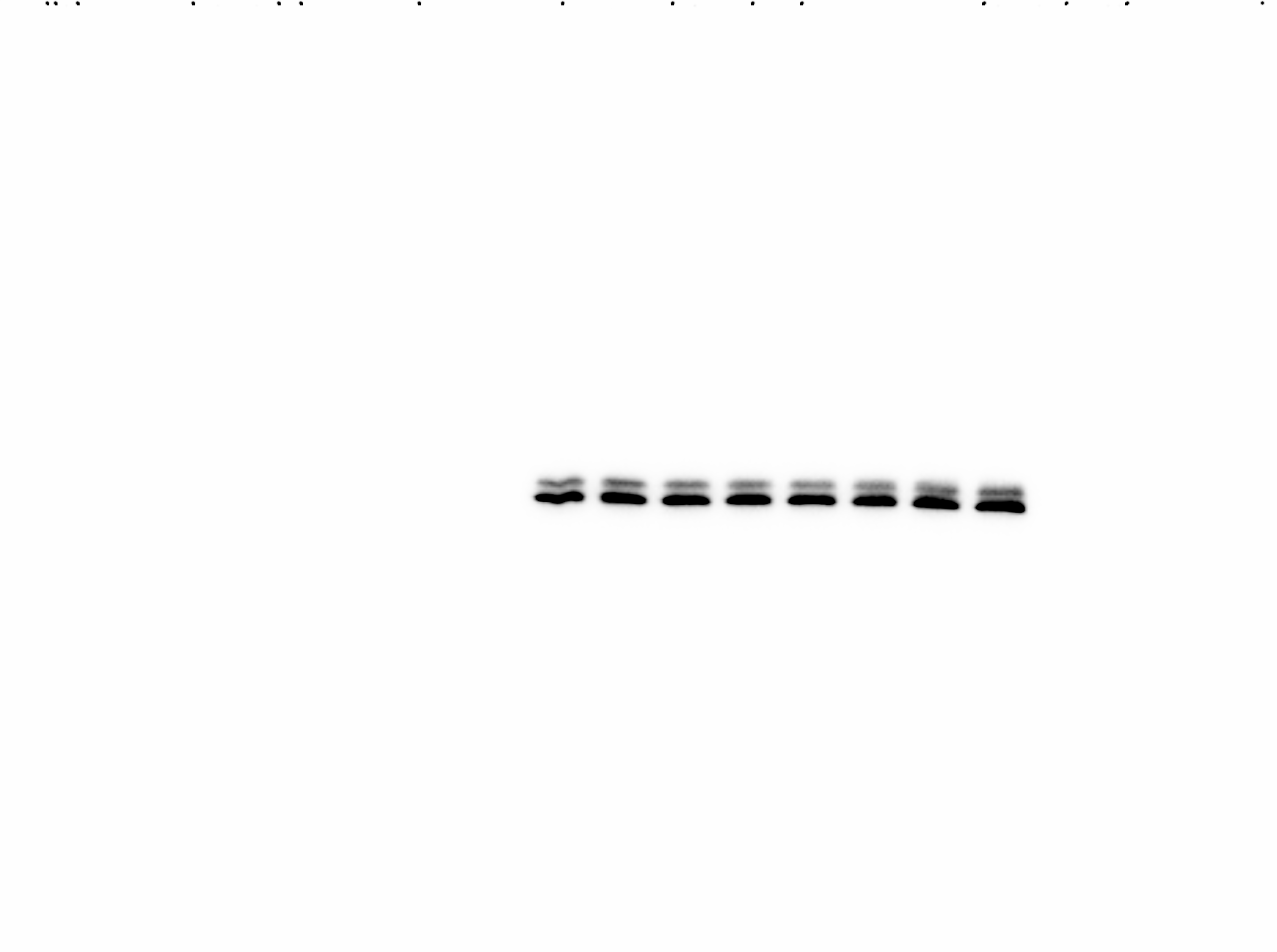

Supplement: Figure 1—source data 2. [file elife-68843-fig1-data2.zip › Figure 1G-Original WB images/Fig.1G ERK.tif]

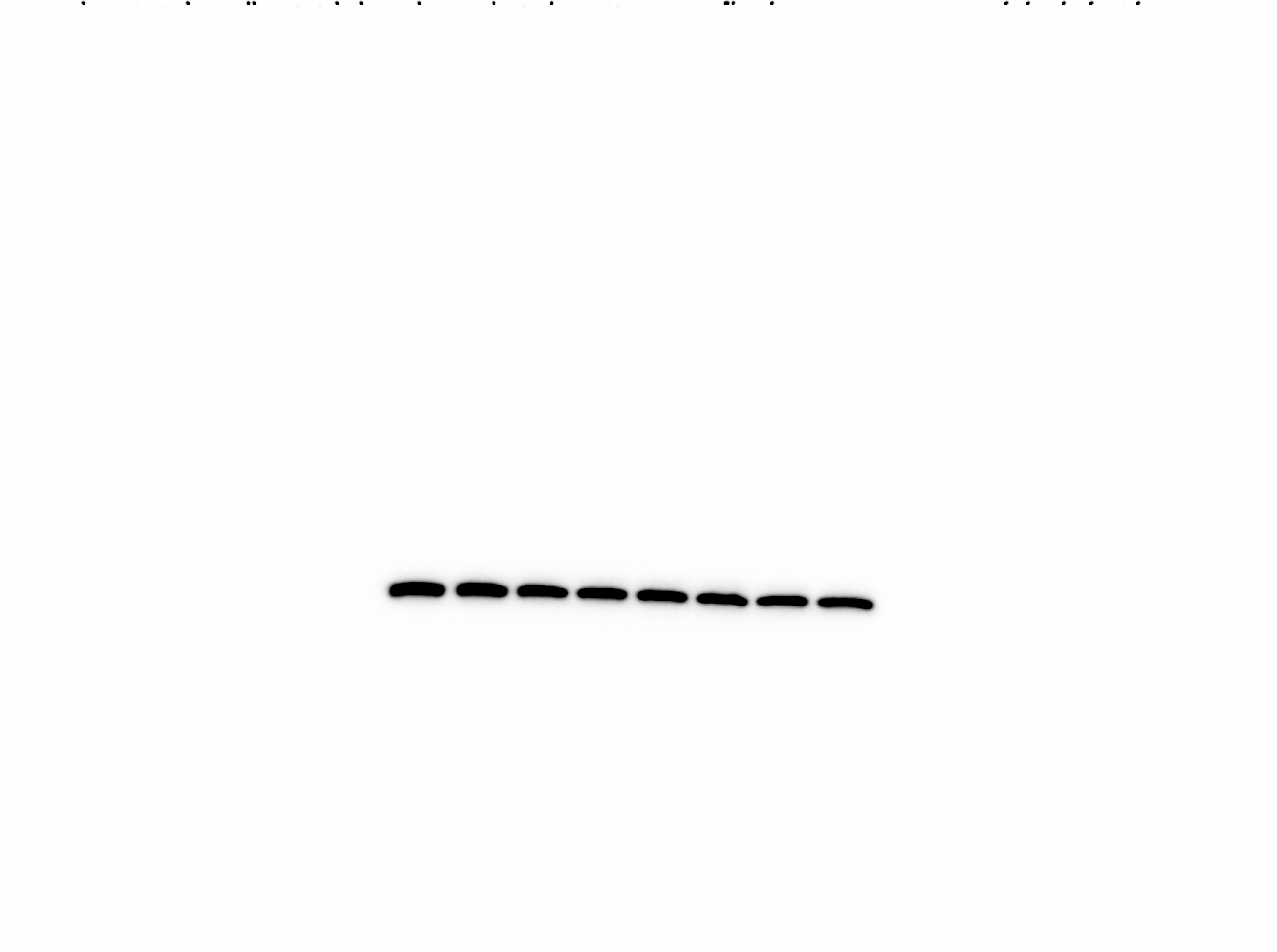

Supplement: Figure 1—source data 2. [file elife-68843-fig1-data2.zip › Figure 1G-Original WB images/Fig.1G GAPDH.tif]

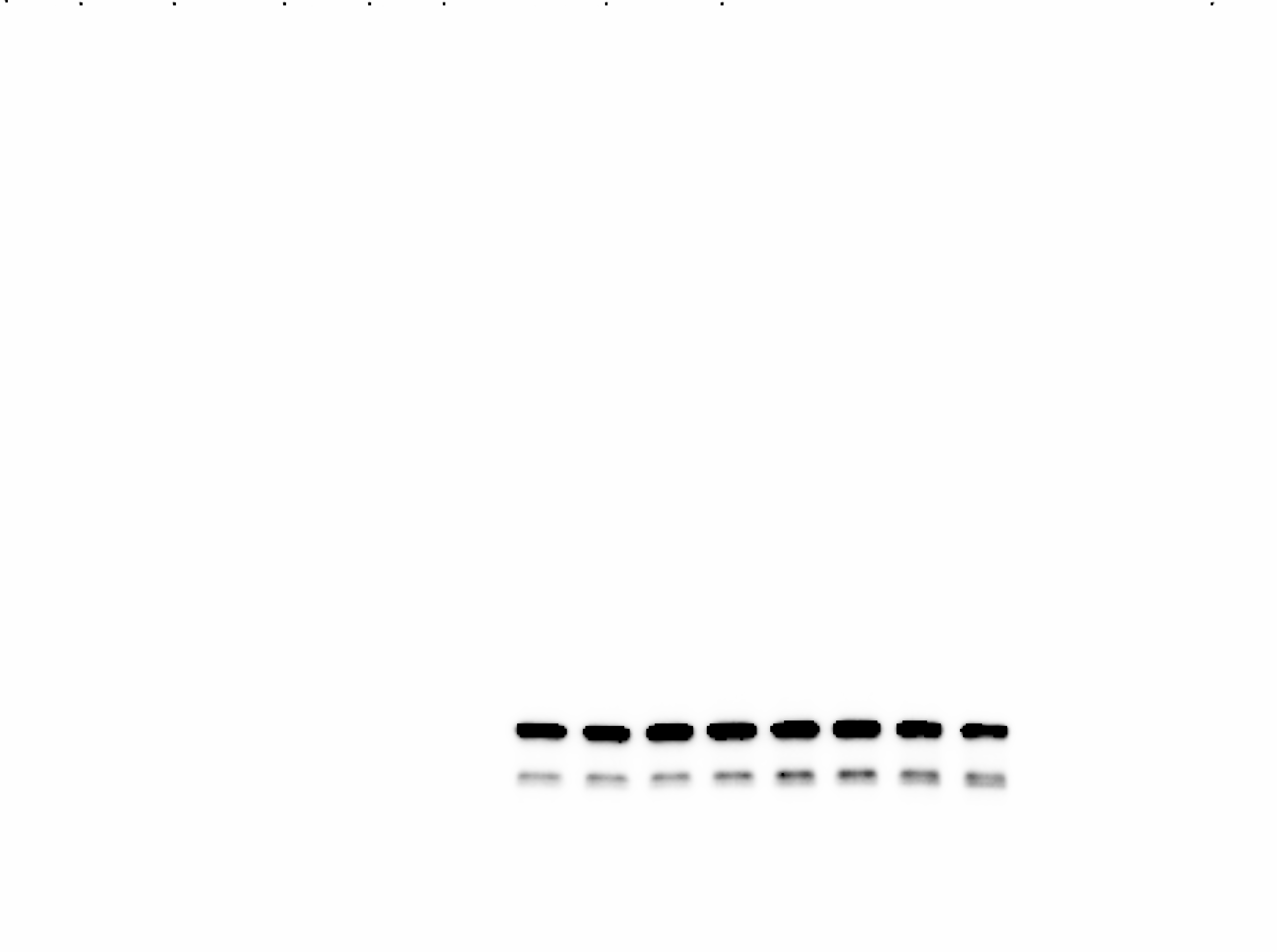

Supplement: Figure 1—source data 2. [file elife-68843-fig1-data2.zip › Figure 1G-Original WB images/Fig.1G JNK.tif]

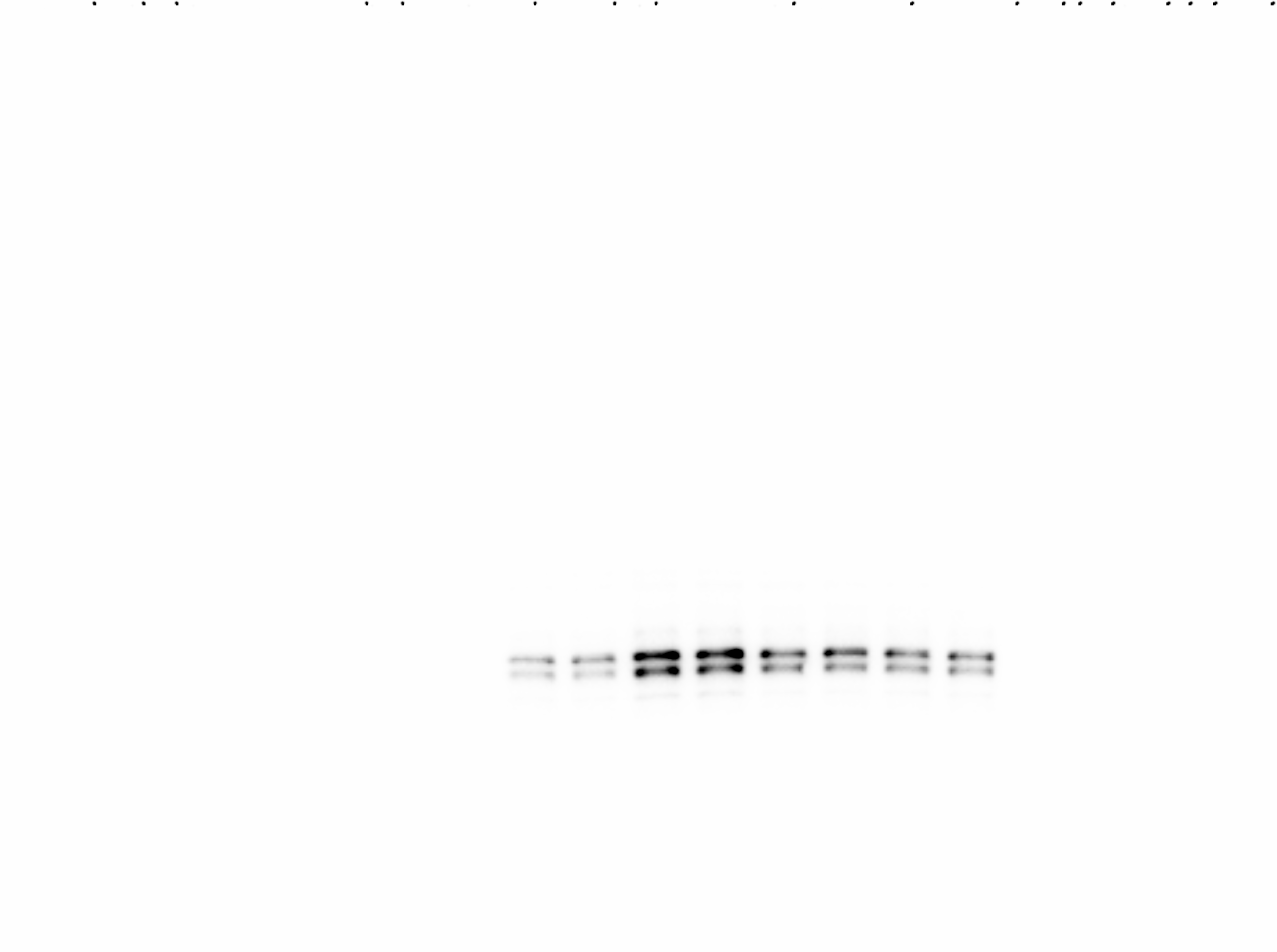

Supplement: Figure 1—source data 2. [file elife-68843-fig1-data2.zip › Figure 1G-Original WB images/Fig.1G NOX4.tif]

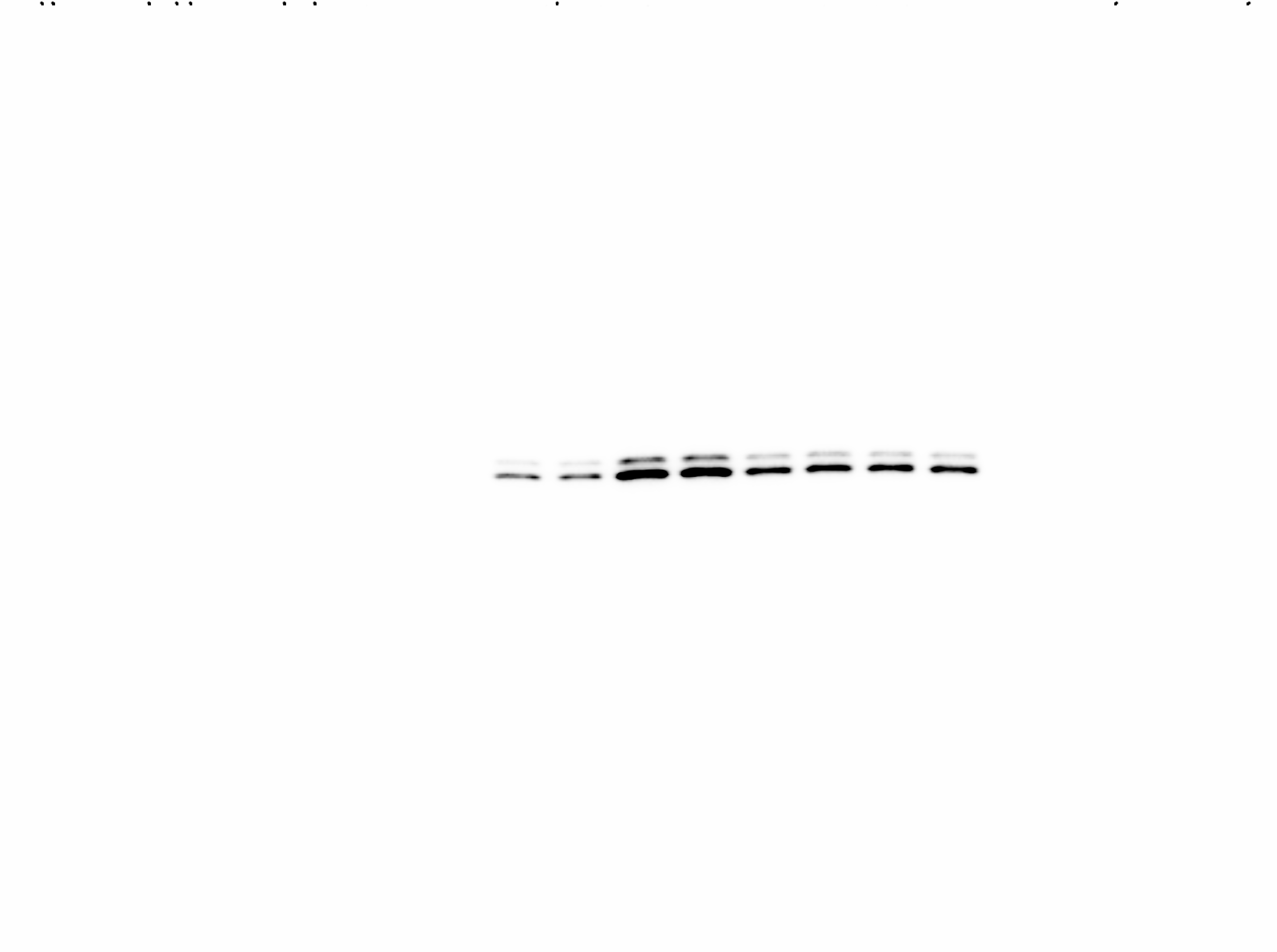

Supplement: Figure 1—source data 2. [file elife-68843-fig1-data2.zip › Figure 1G-Original WB images/Fig.1G p-ERK.tif]

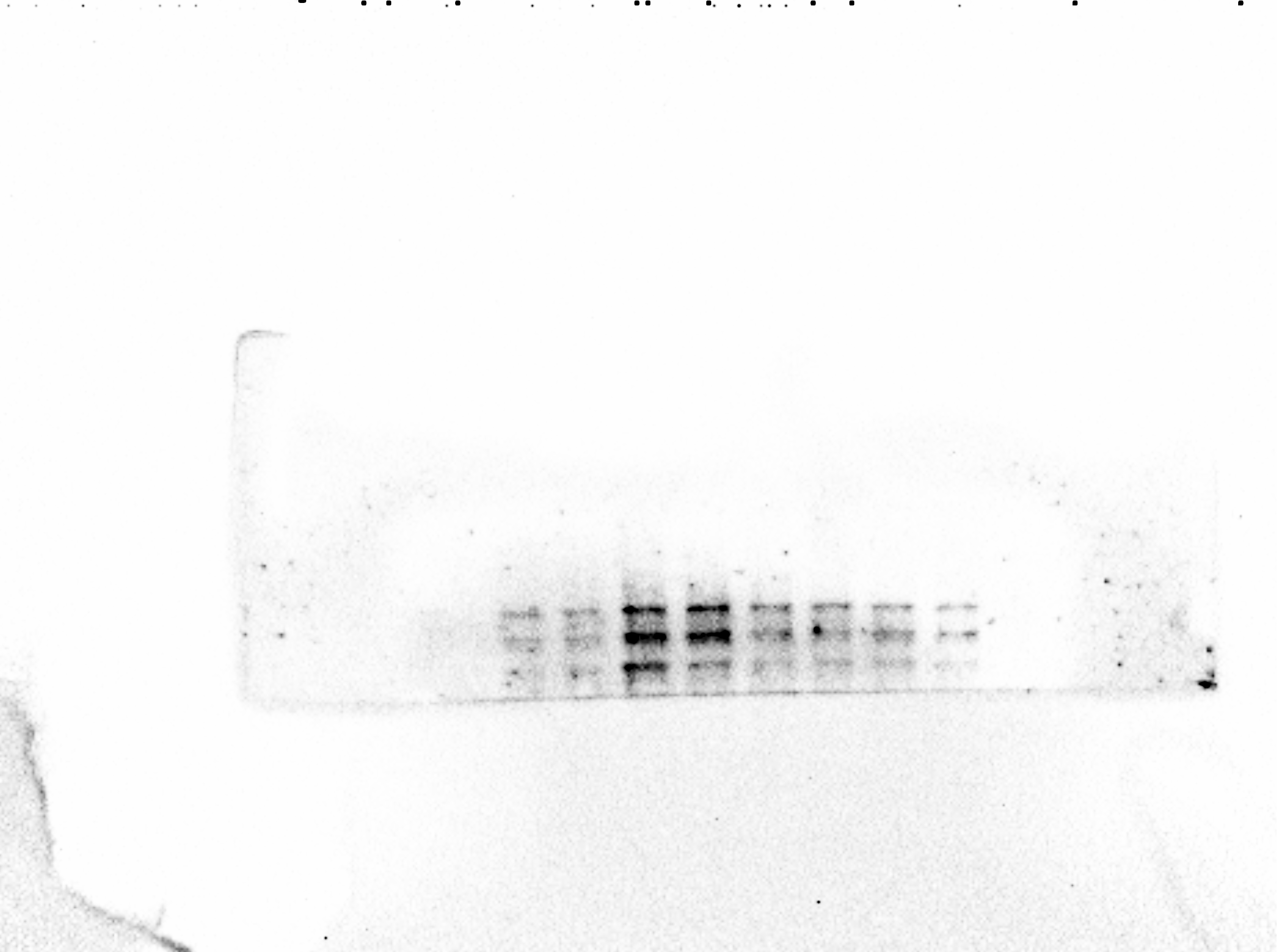

Supplement: Figure 1—source data 2. [file elife-68843-fig1-data2.zip › Figure 1G-Original WB images/Fig.1G p-JNK.tif]

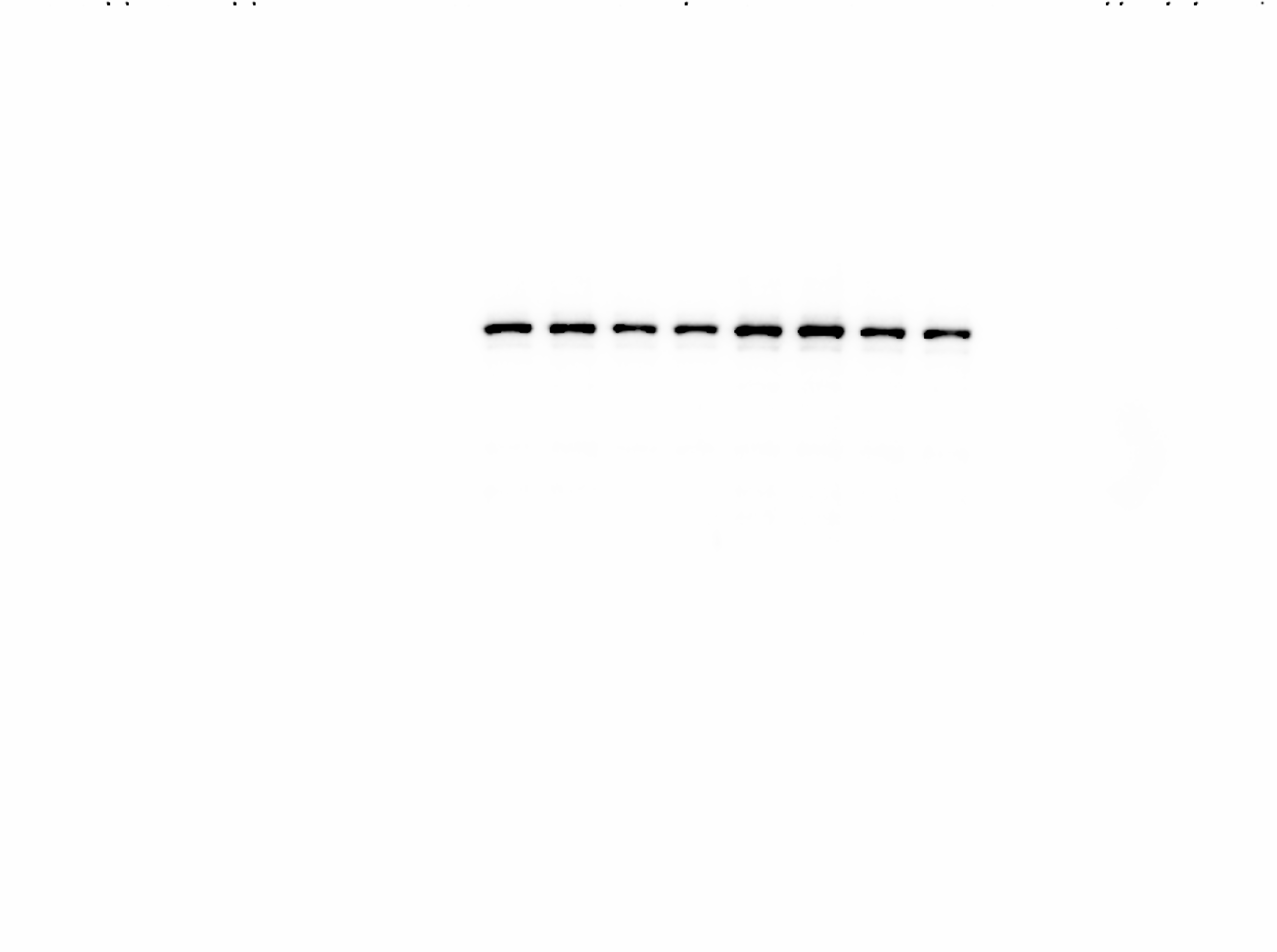

Supplement: Figure 1—source data 2. [file elife-68843-fig1-data2.zip › Figure 1G-Original WB images/Fig.1G p-STAT3.tif]

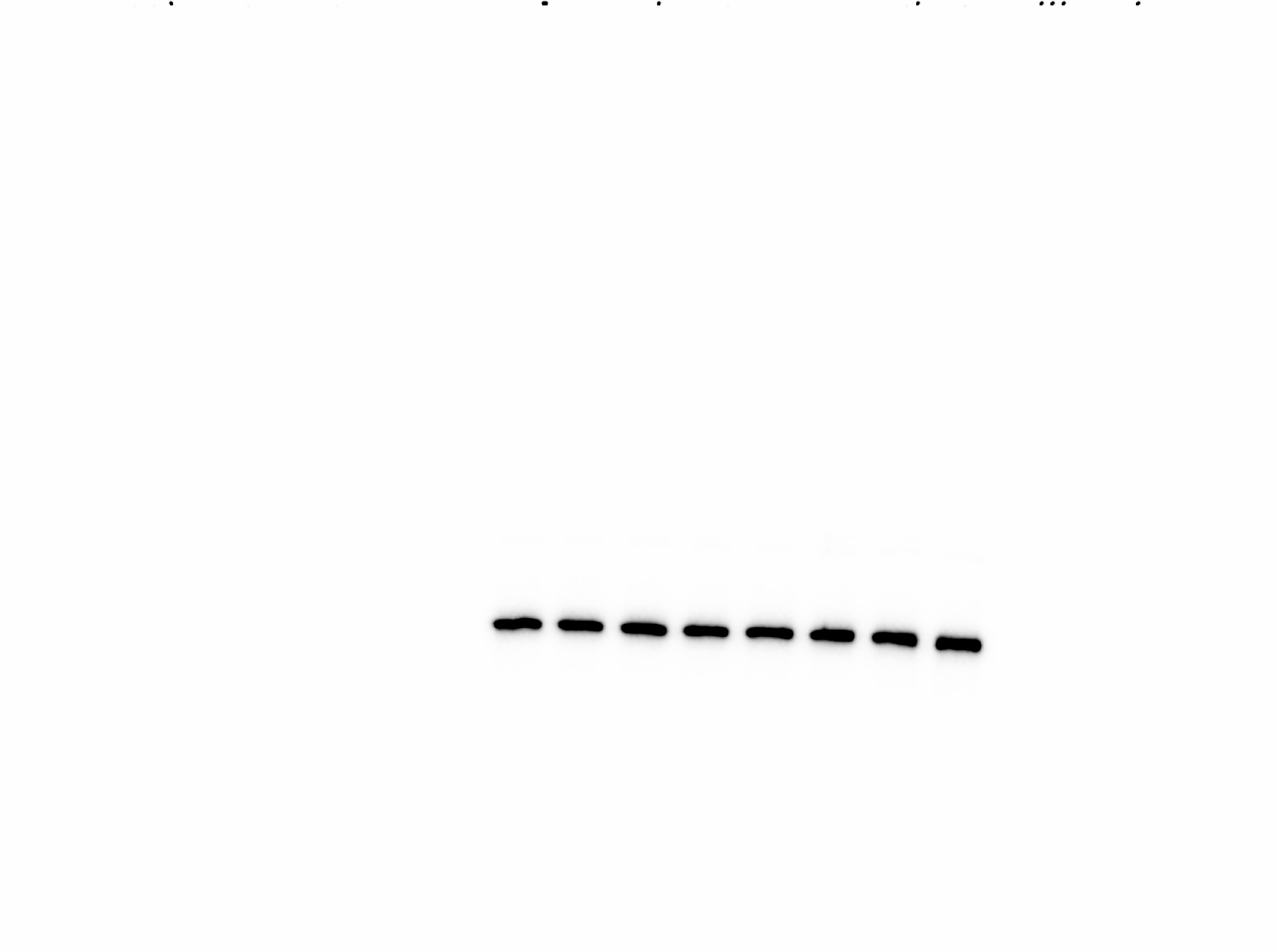

Supplement: Figure 1—source data 2. [file elife-68843-fig1-data2.zip › Figure 1G-Original WB images/Fig.1G STAT3.tif]

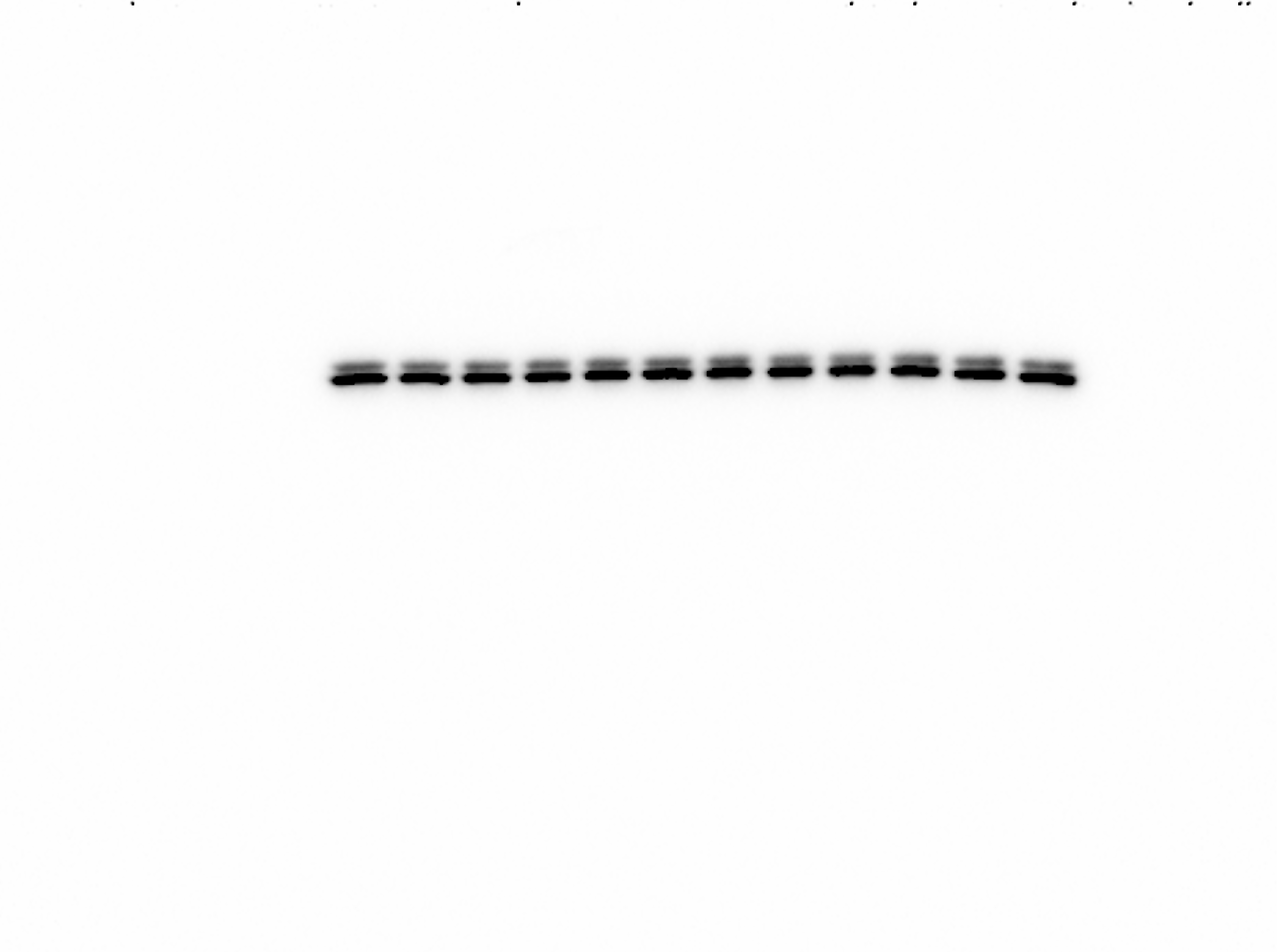

Supplement: Figure 1—source data 2. [file elife-68843-fig1-data2.zip › Figure 1J-Original WB images/Fig.1J ERK.tif]

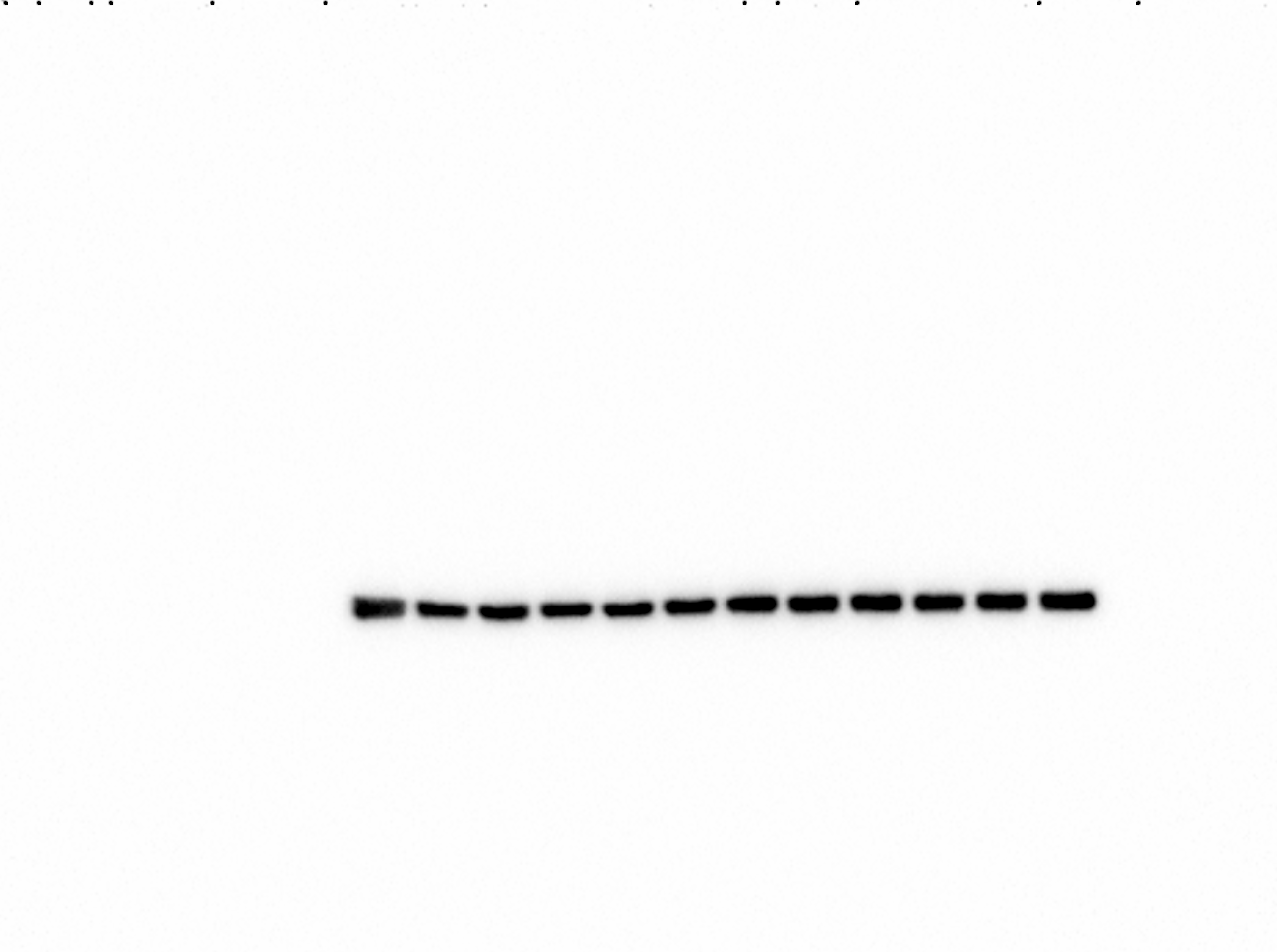

Supplement: Figure 1—source data 2. [file elife-68843-fig1-data2.zip › Figure 1J-Original WB images/Fig.1J GAPDH.tif]

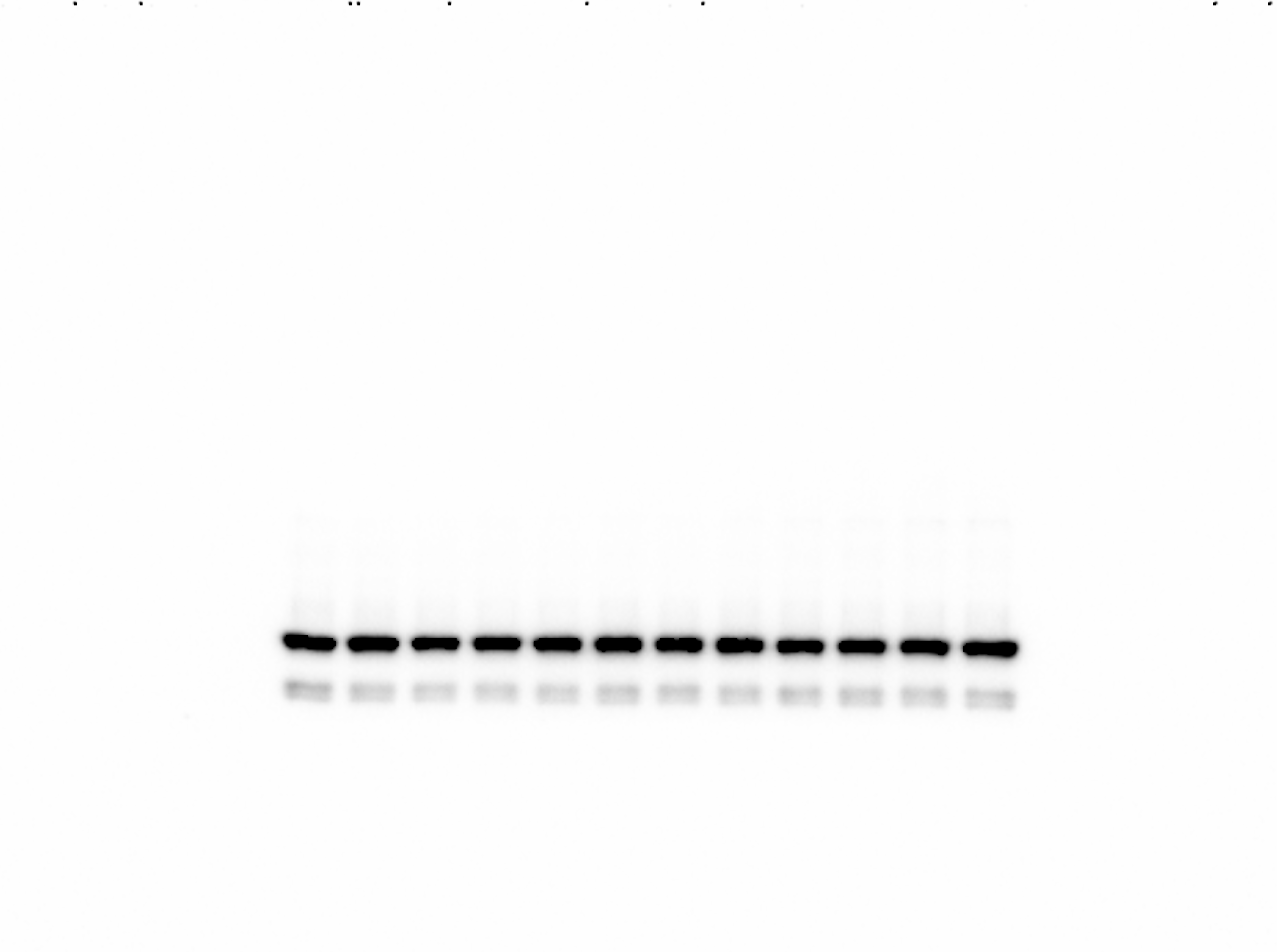

Supplement: Figure 1—source data 2. [file elife-68843-fig1-data2.zip › Figure 1J-Original WB images/Fig.1J JNK.tif]

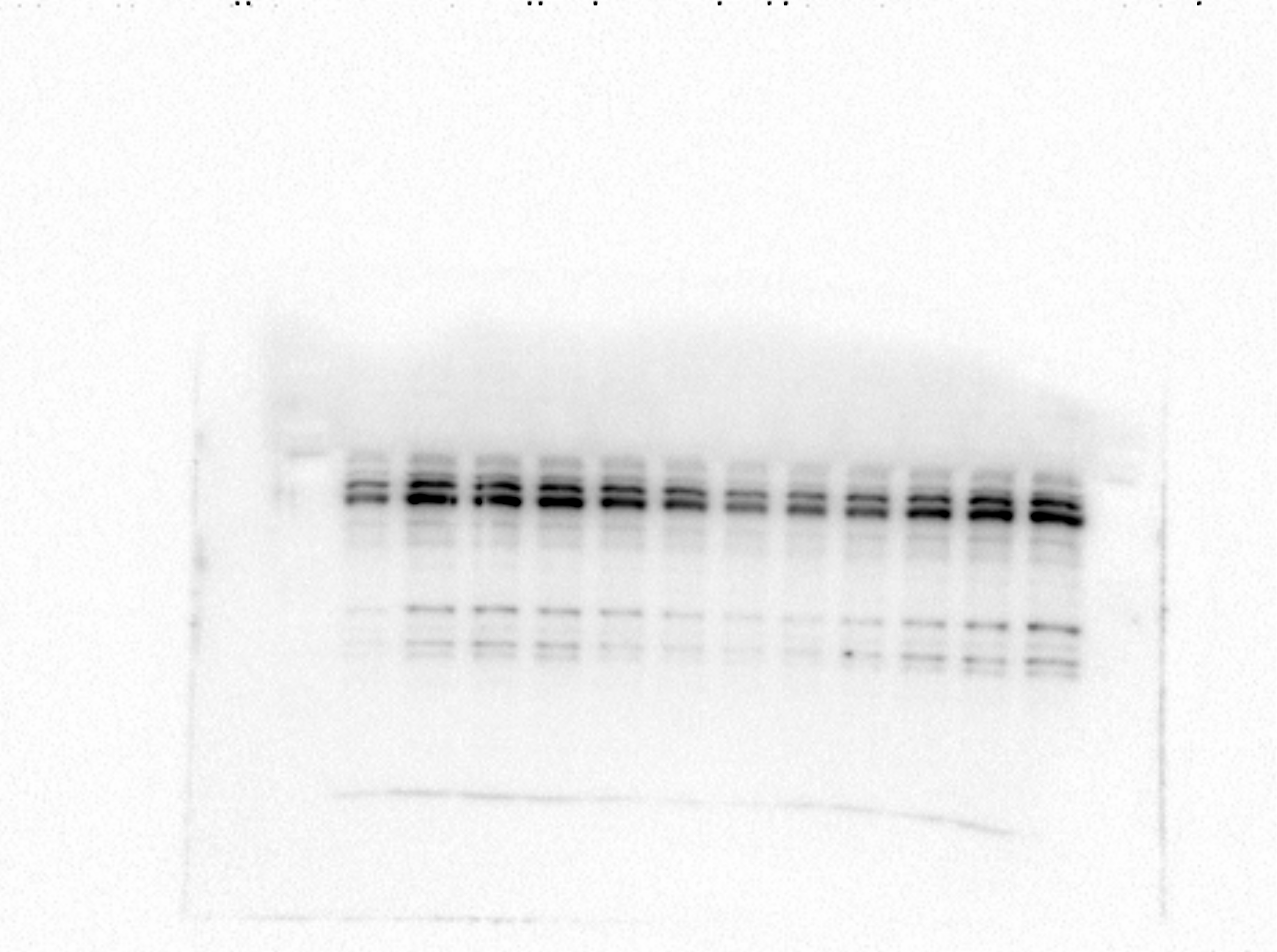

Supplement: Figure 1—source data 2. [file elife-68843-fig1-data2.zip › Figure 1J-Original WB images/Fig.1J NOX4.tif]

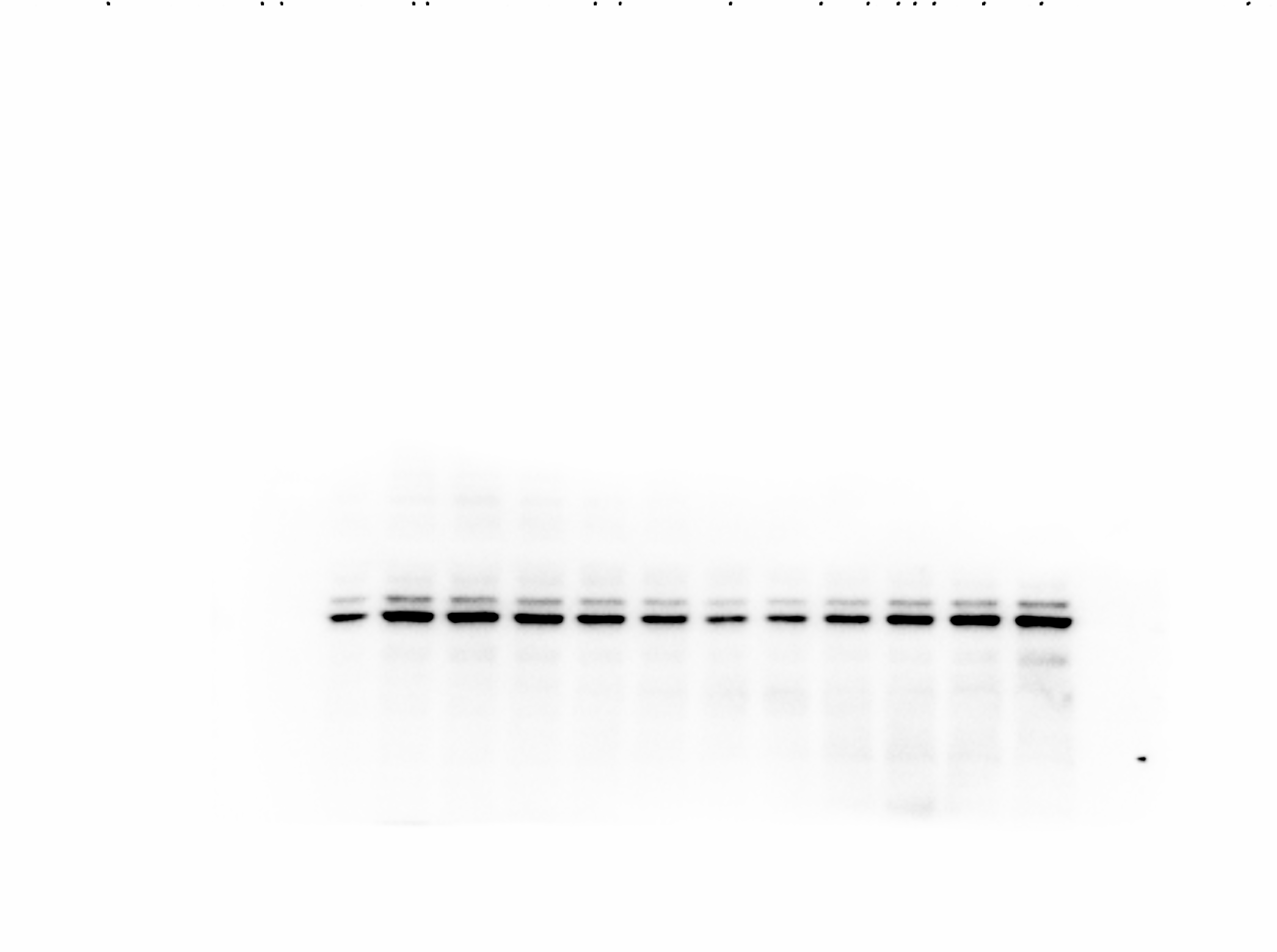

Supplement: Figure 1—source data 2. [file elife-68843-fig1-data2.zip › Figure 1J-Original WB images/Fig.1J p-ERK.tif]

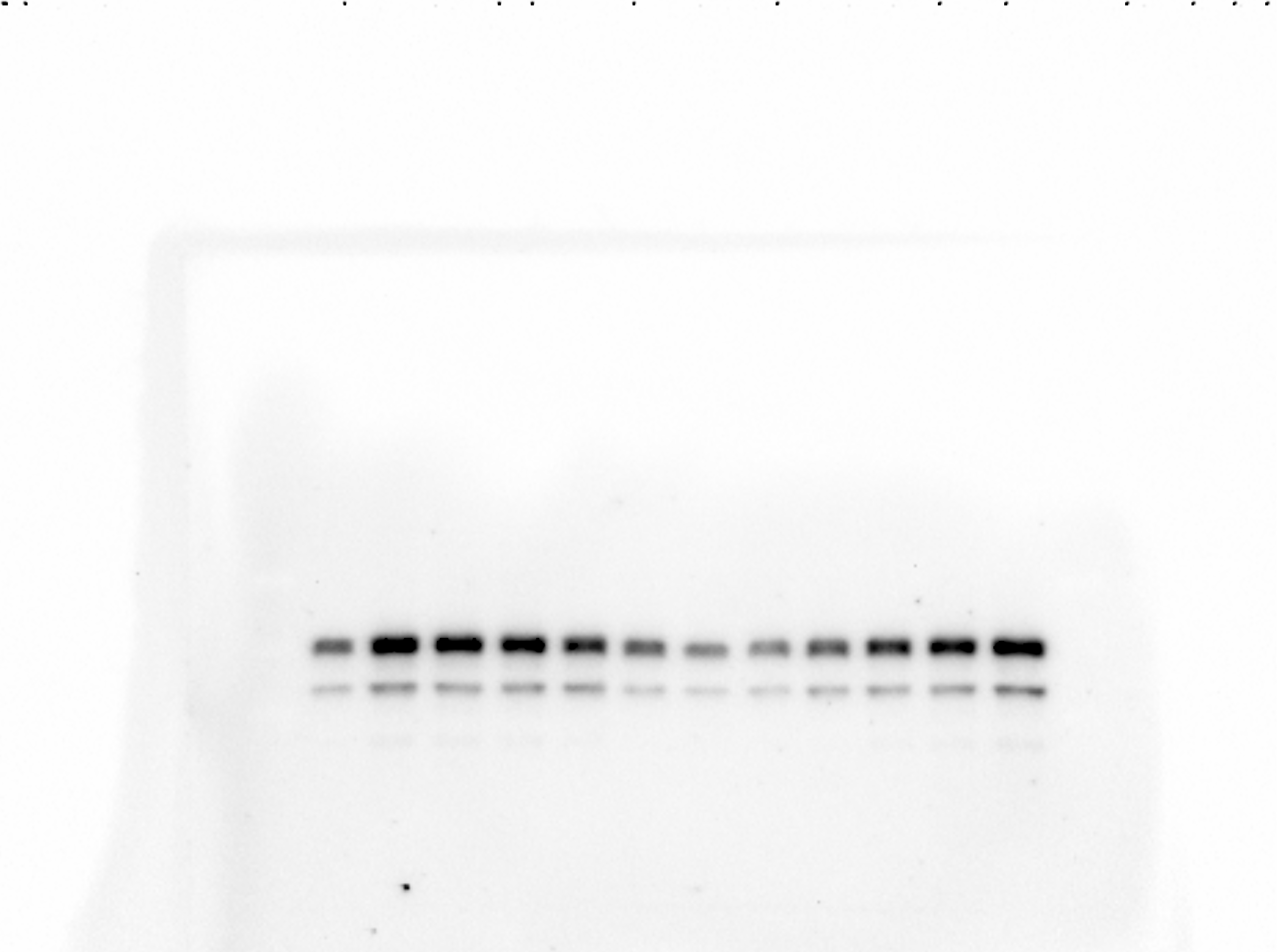

Supplement: Figure 1—source data 2. [file elife-68843-fig1-data2.zip › Figure 1J-Original WB images/Fig.1J p-JNK.tif]

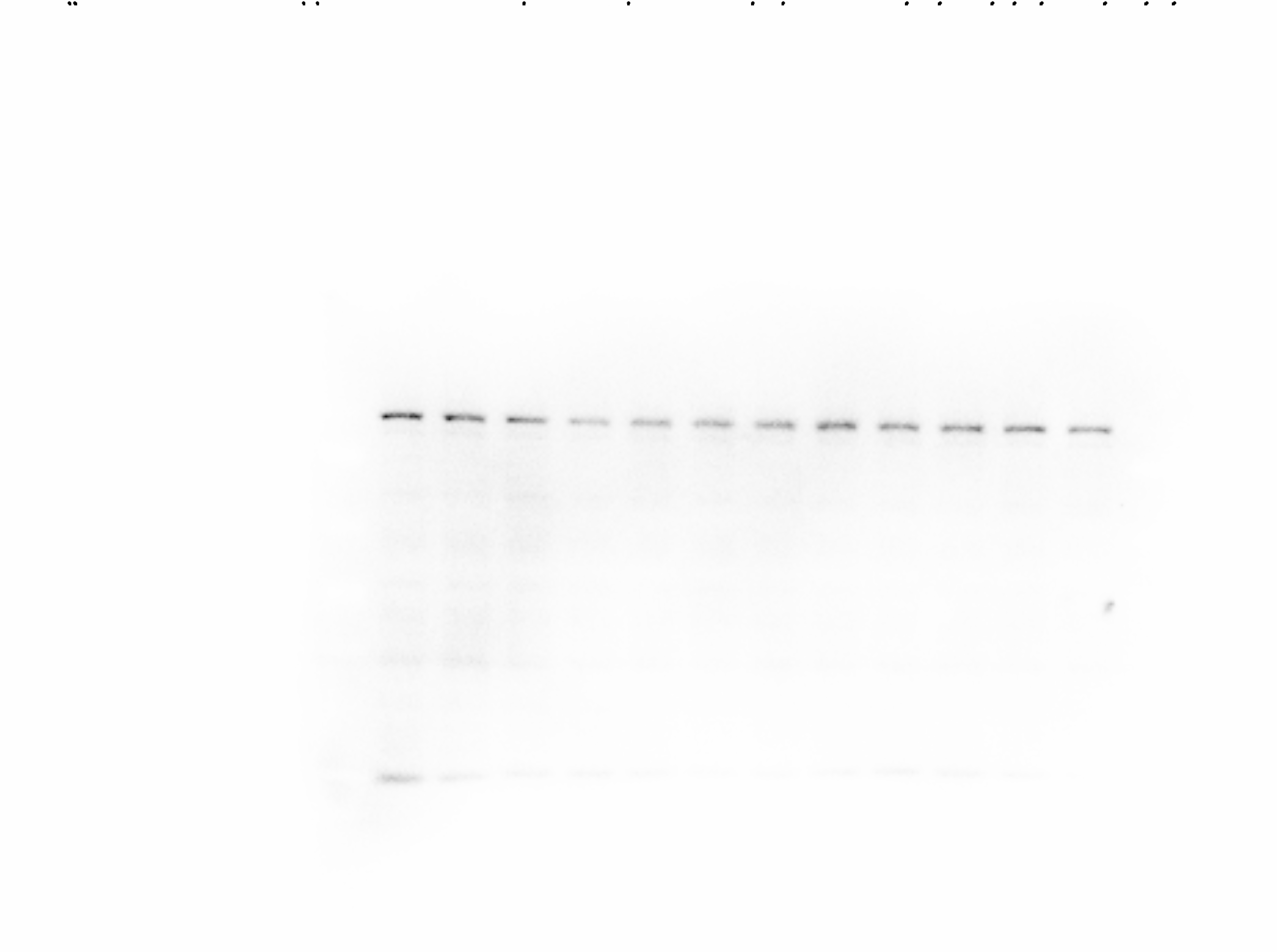

Supplement: Figure 1—source data 2. [file elife-68843-fig1-data2.zip › Figure 1J-Original WB images/Fig.1J p-STAT3.tif]

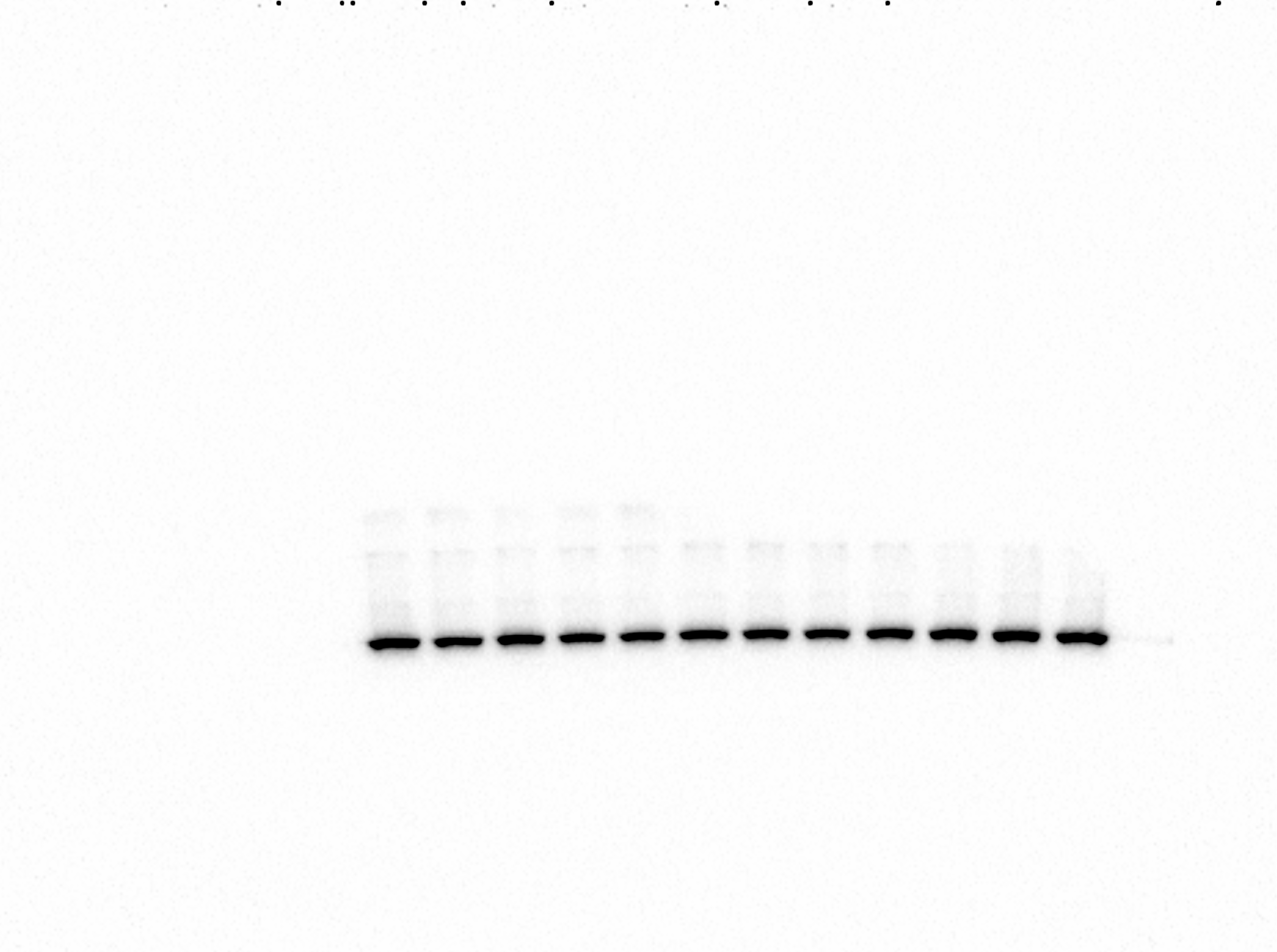

Supplement: Figure 1—source data 2. [file elife-68843-fig1-data2.zip › Figure 1J-Original WB images/Fig.1J STAT3.tif]

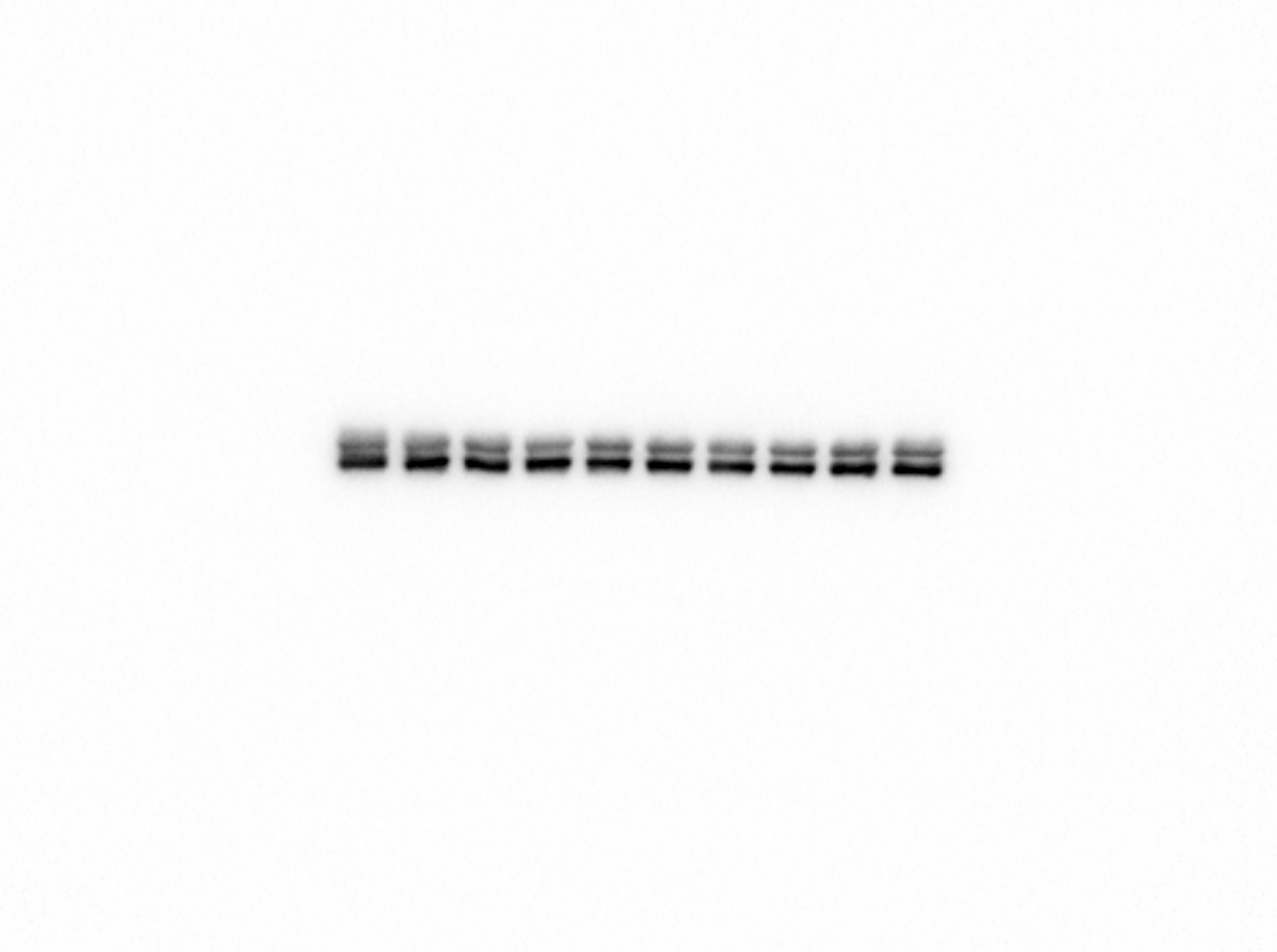

Supplement: Figure 1—figure supplement 1—source data 2. [file elife-68843-fig1-figsupp1-data2.zip › Figure 1-figure supplement 1F-Original WB images/Fig. S1F ERK.tif]

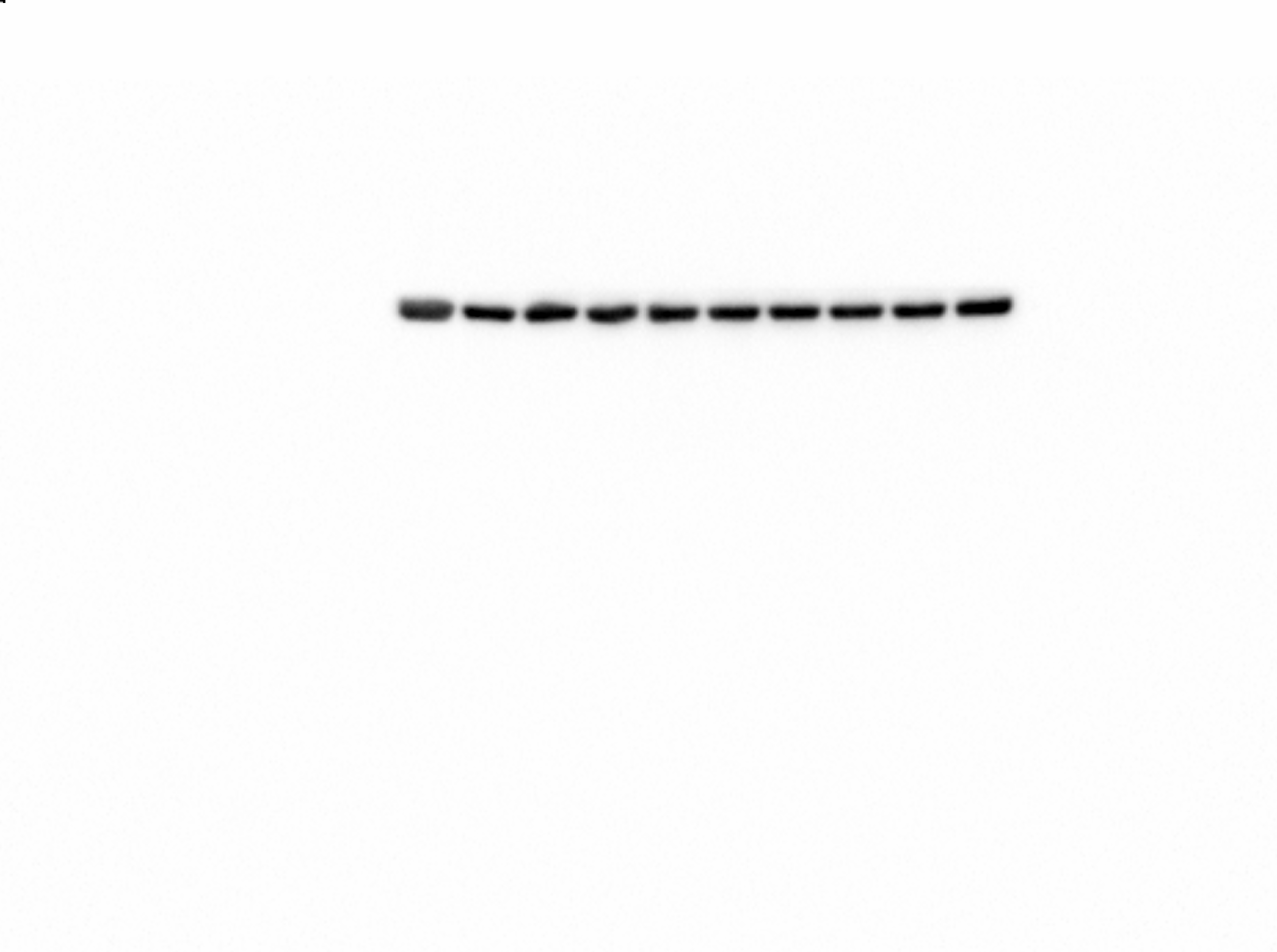

Supplement: Figure 1—figure supplement 1—source data 2. [file elife-68843-fig1-figsupp1-data2.zip › Figure 1-figure supplement 1F-Original WB images/Fig. S1F GAPDH.tif]

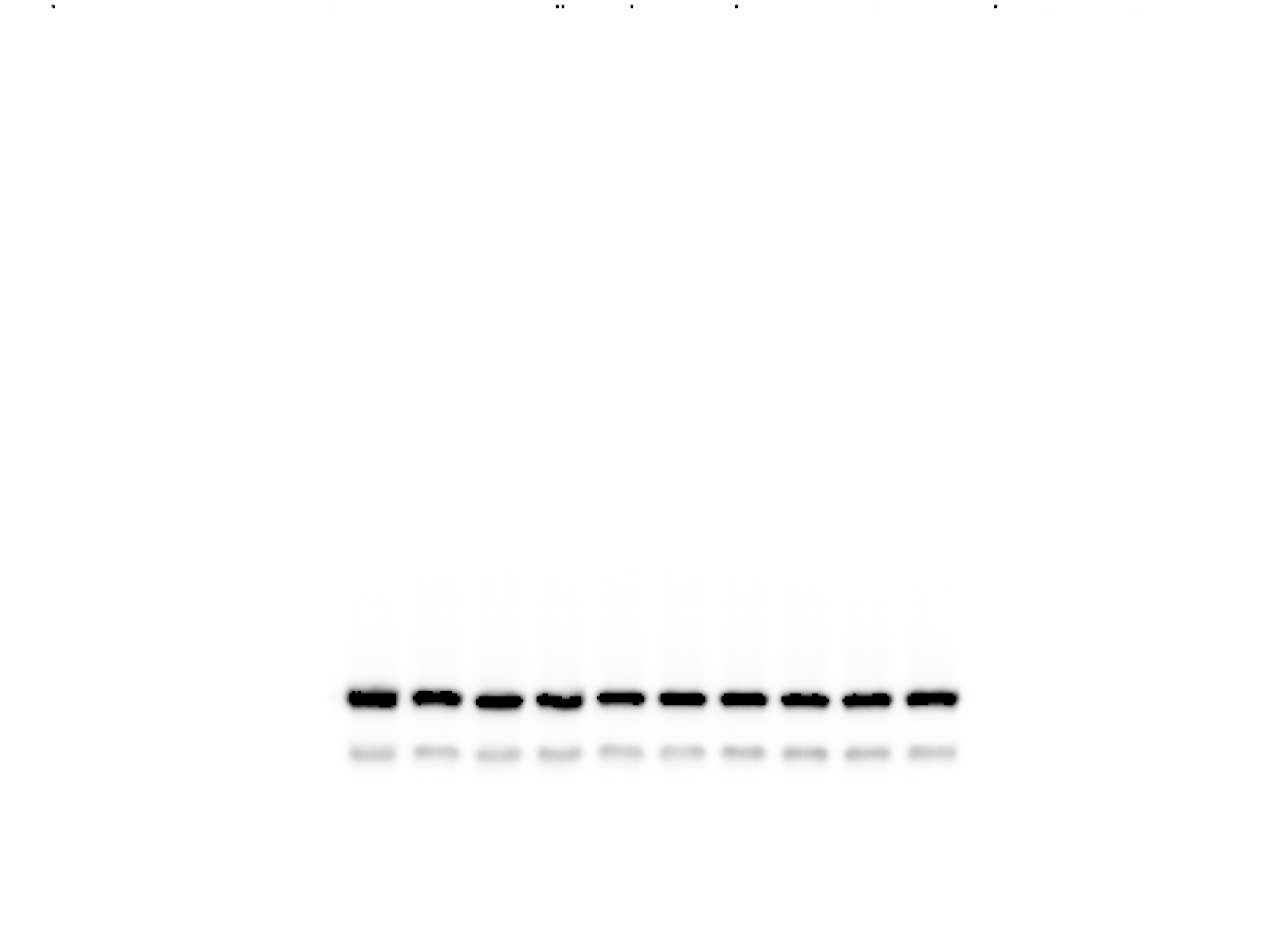

Supplement: Figure 1—figure supplement 1—source data 2. [file elife-68843-fig1-figsupp1-data2.zip › Figure 1-figure supplement 1F-Original WB images/Fig. S1F JNK.tif]

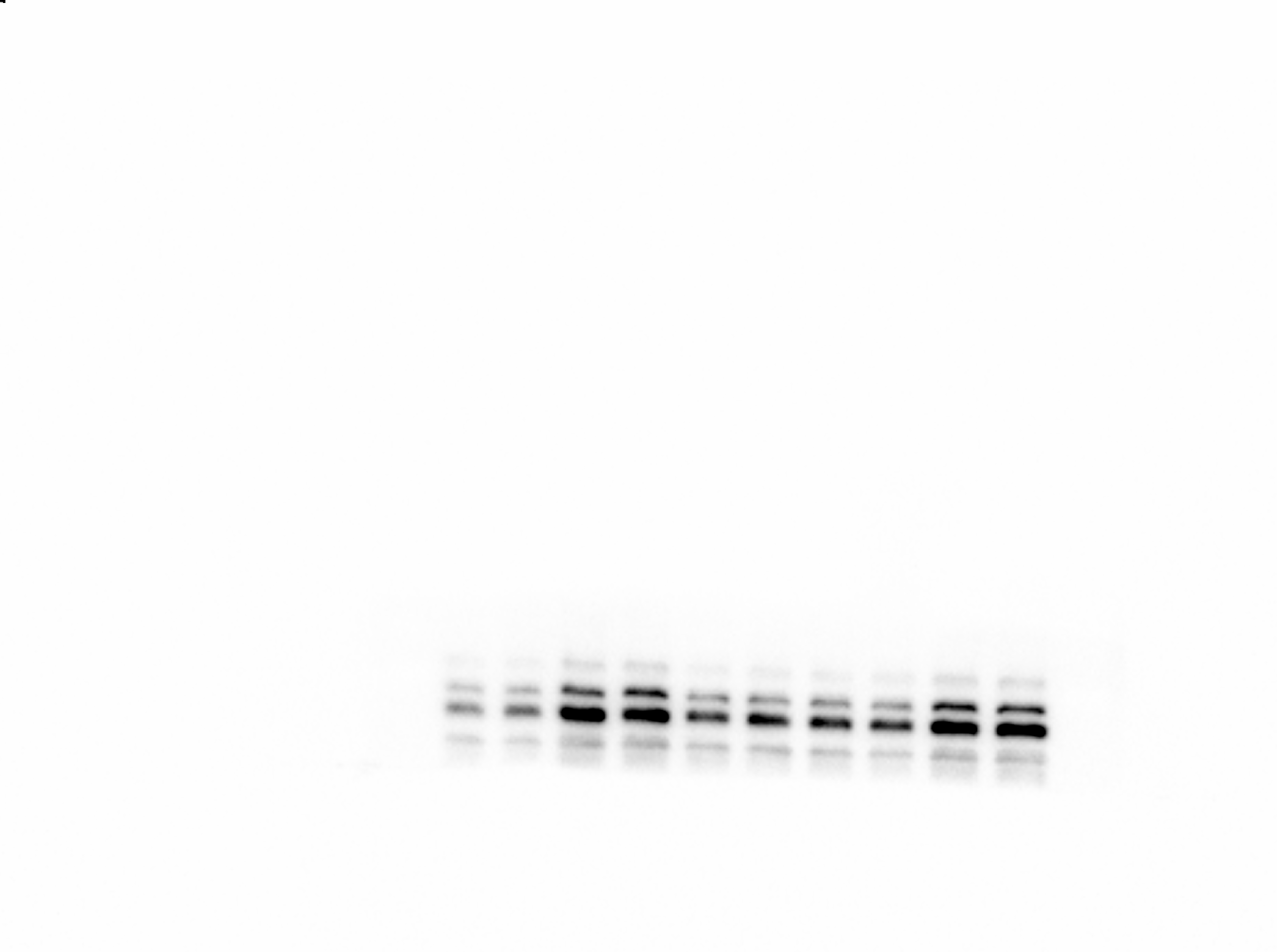

Supplement: Figure 1—figure supplement 1—source data 2. [file elife-68843-fig1-figsupp1-data2.zip › Figure 1-figure supplement 1F-Original WB images/Fig. S1F NOX4.tif]

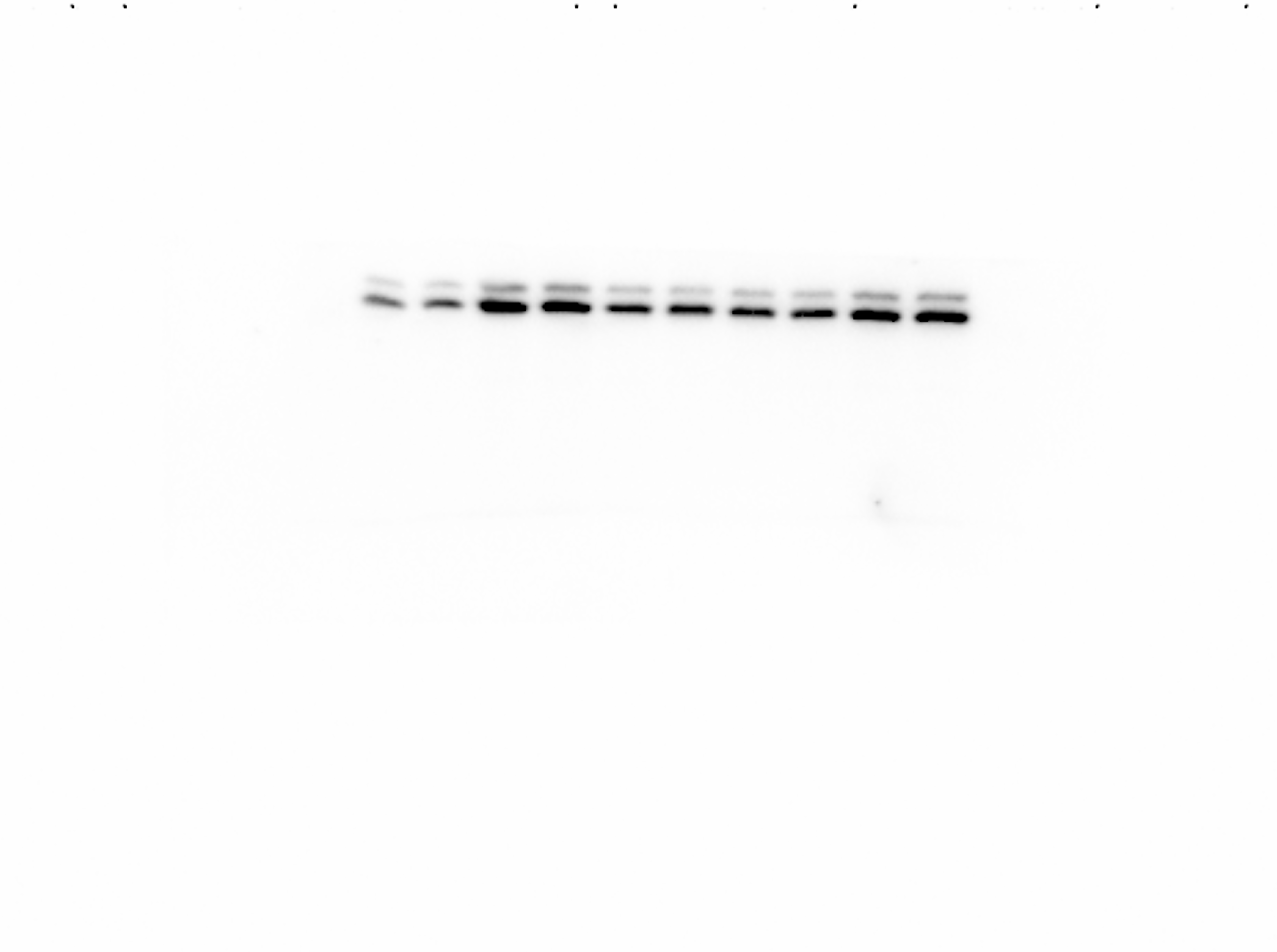

Supplement: Figure 1—figure supplement 1—source data 2. [file elife-68843-fig1-figsupp1-data2.zip › Figure 1-figure supplement 1F-Original WB images/Fig. S1F p-ERK.tif]

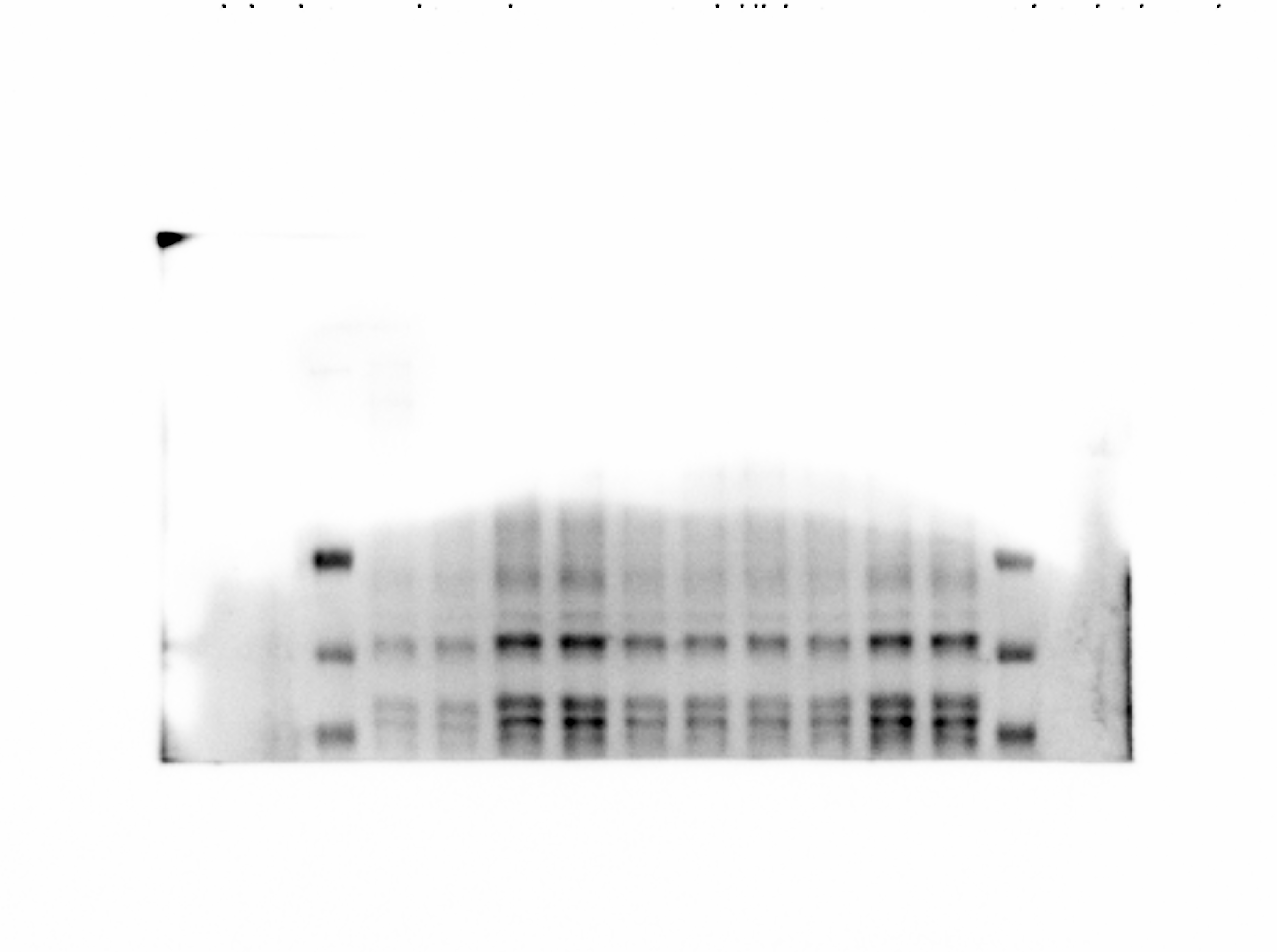

Supplement: Figure 1—figure supplement 1—source data 2. [file elife-68843-fig1-figsupp1-data2.zip › Figure 1-figure supplement 1F-Original WB images/Fig. S1F p-JNK.tif]

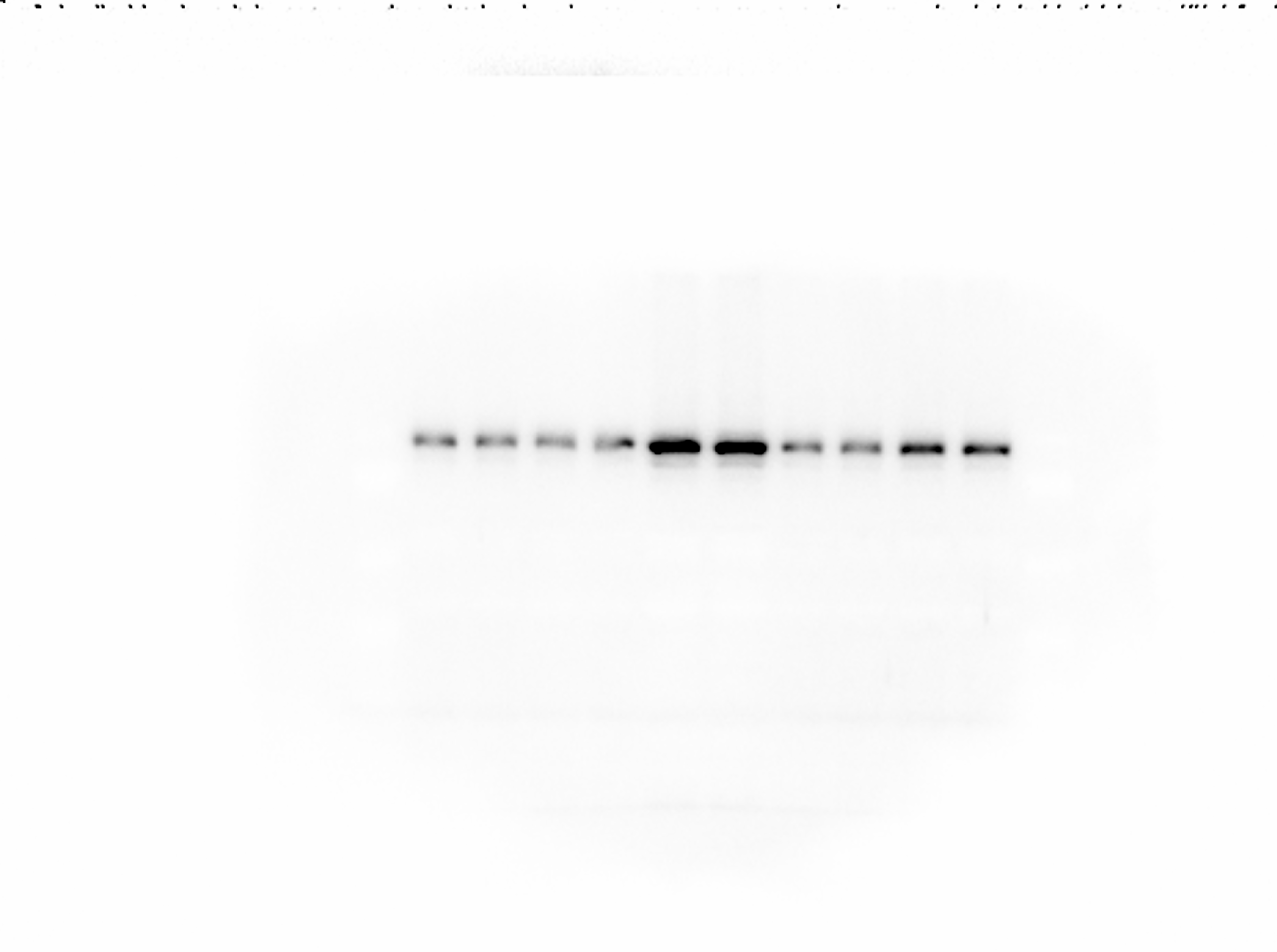

Supplement: Figure 1—figure supplement 1—source data 2. [file elife-68843-fig1-figsupp1-data2.zip › Figure 1-figure supplement 1F-Original WB images/Fig. S1F p-STAT3.tif]

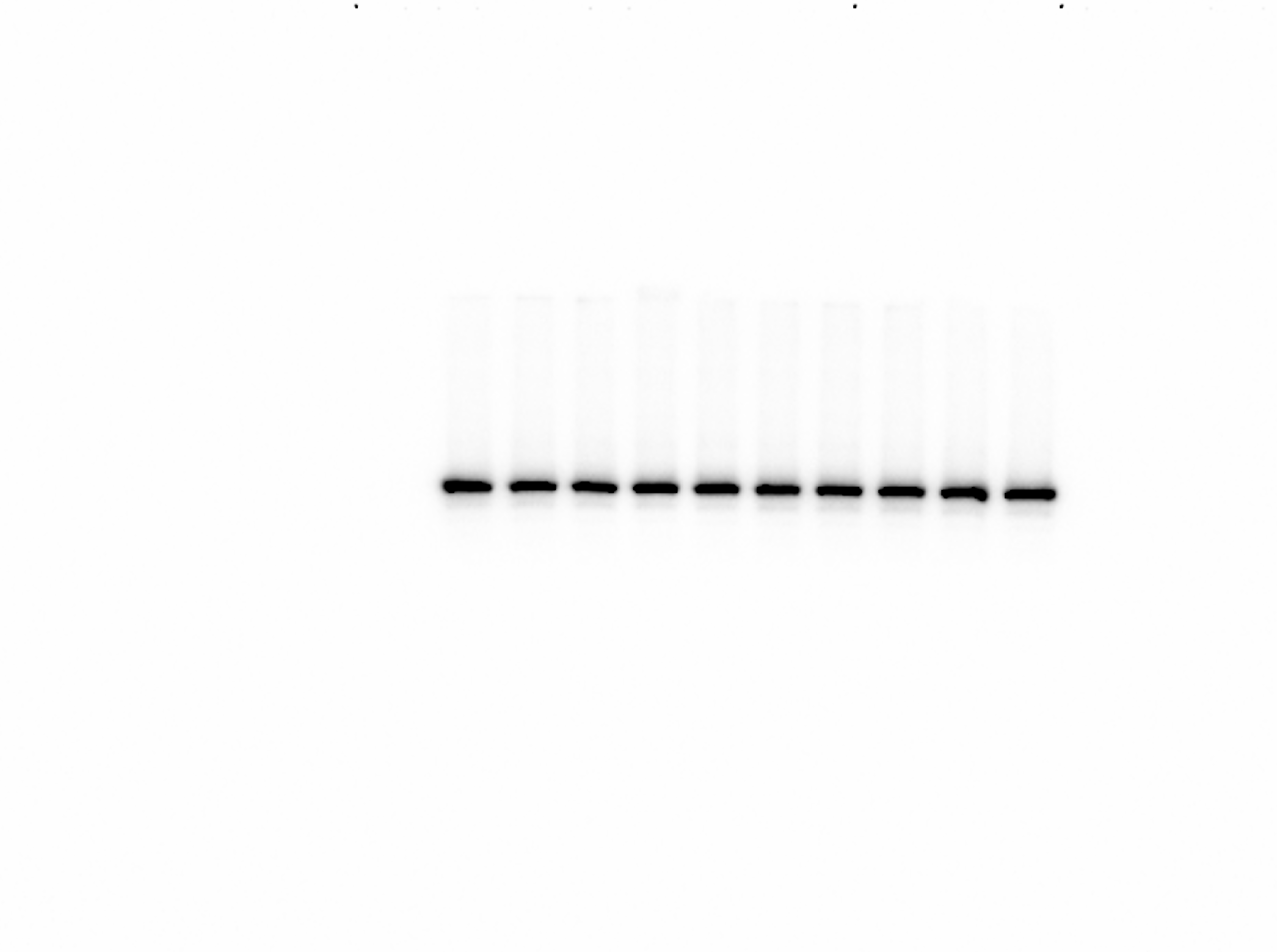

Supplement: Figure 1—figure supplement 1—source data 2. [file elife-68843-fig1-figsupp1-data2.zip › Figure 1-figure supplement 1F-Original WB images/Fig. S1F STAT3.tif]

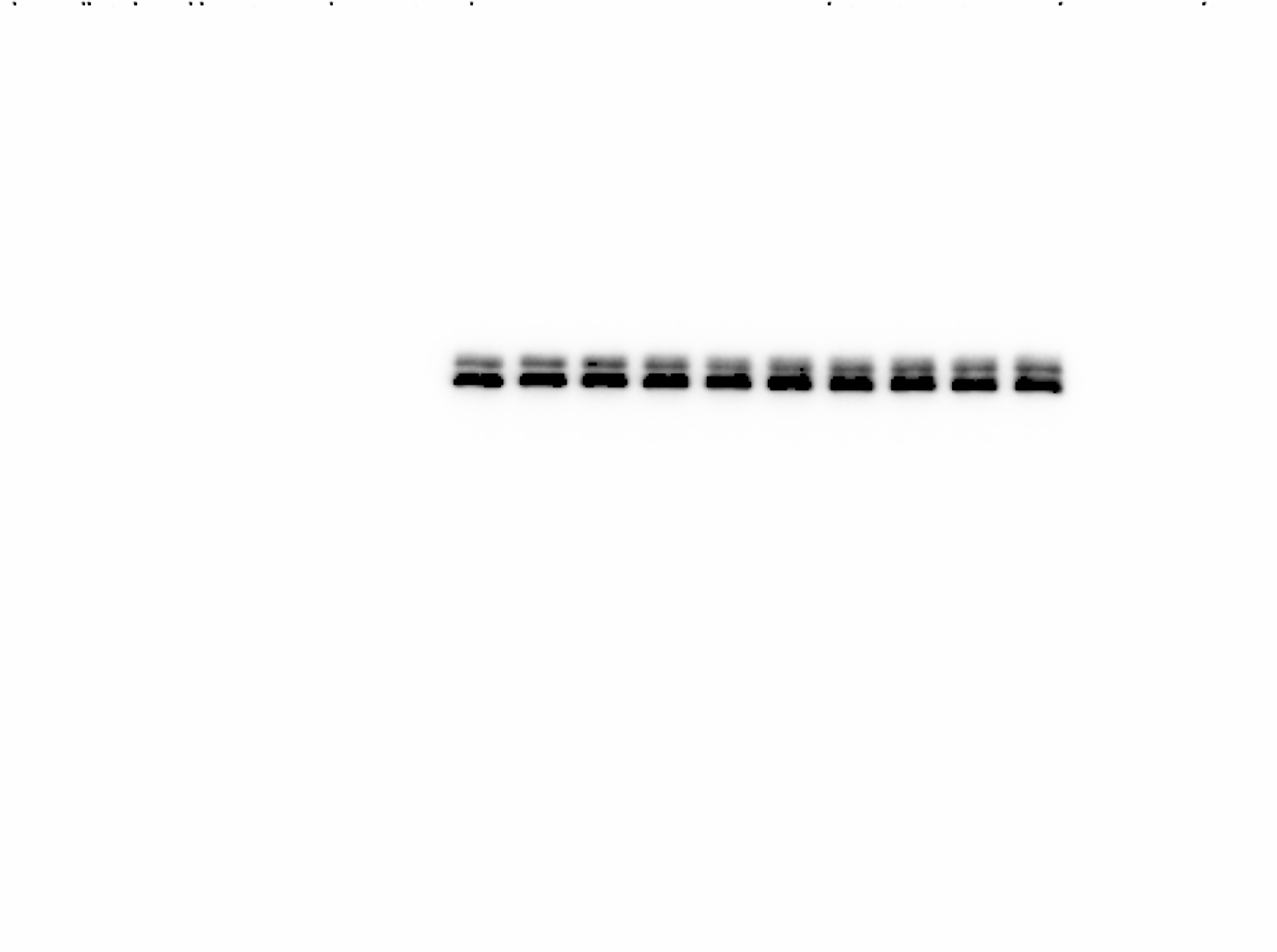

Supplement: Figure 1—figure supplement 1—source data 2. [file elife-68843-fig1-figsupp1-data2.zip › Figure 1-figure supplement 1G-Original WB images/Fig. S1G ERK.tif]

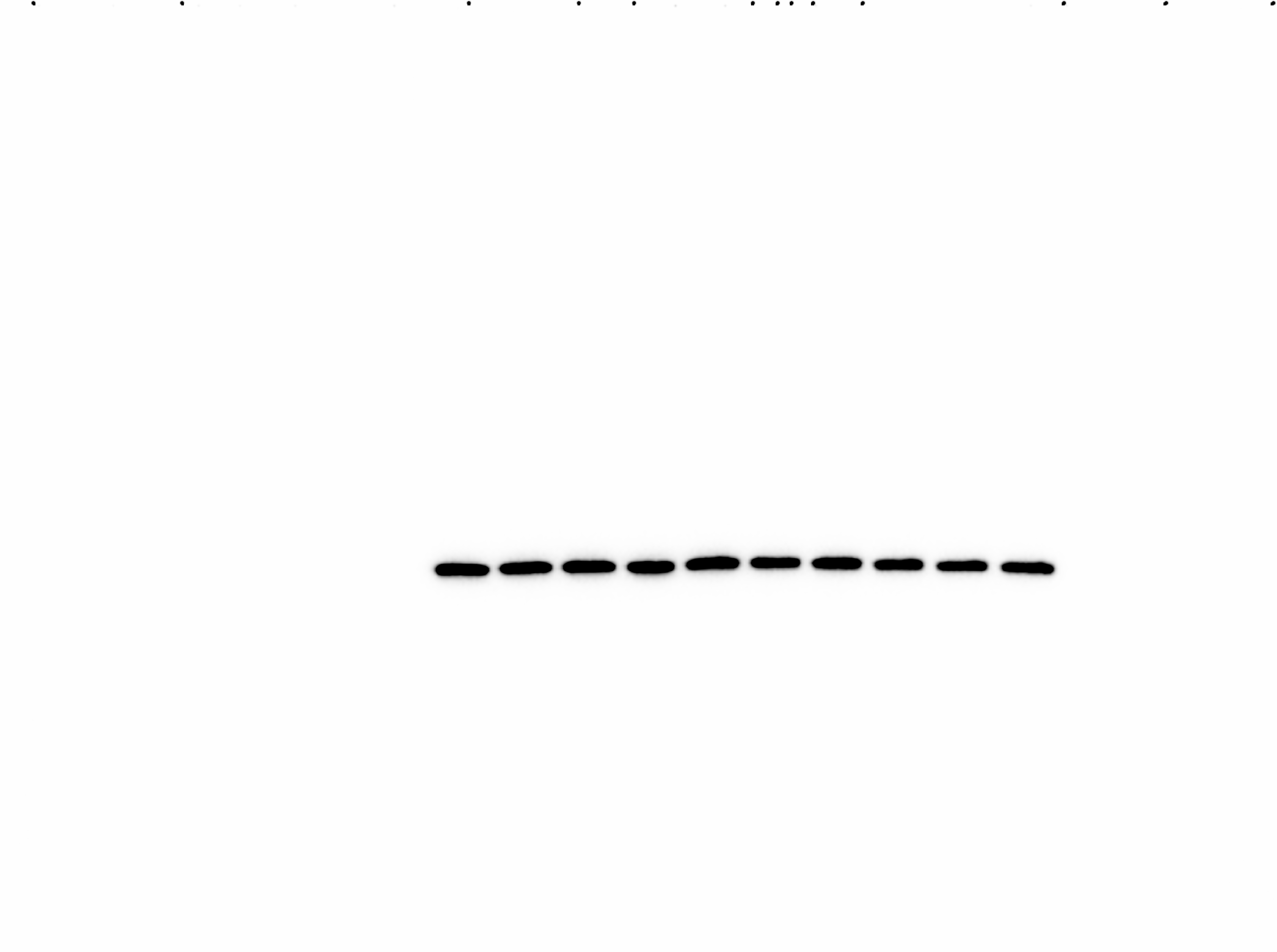

Supplement: Figure 1—figure supplement 1—source data 2. [file elife-68843-fig1-figsupp1-data2.zip › Figure 1-figure supplement 1G-Original WB images/Fig. S1G GAPDH.tif]

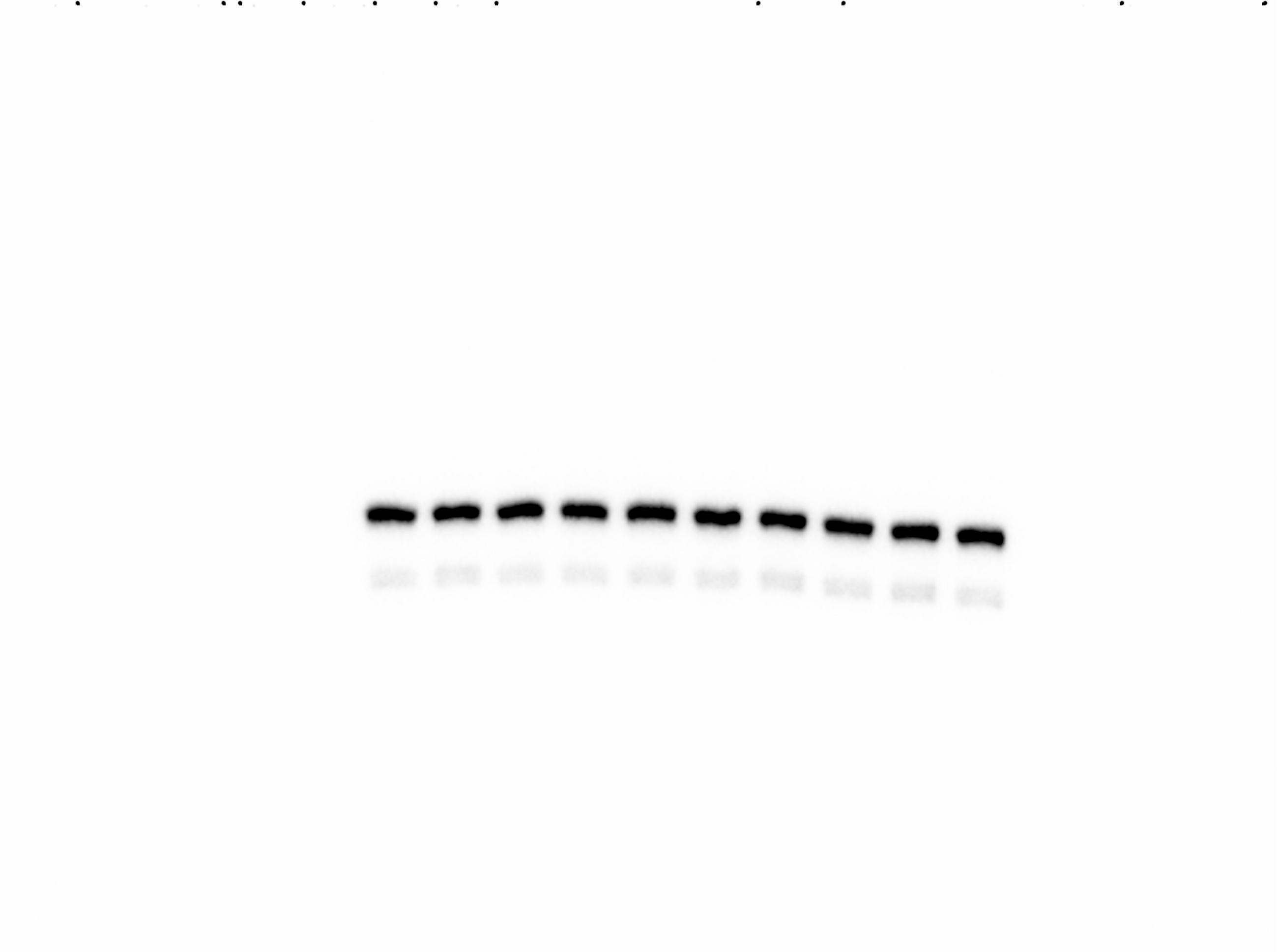

Supplement: Figure 1—figure supplement 1—source data 2. [file elife-68843-fig1-figsupp1-data2.zip › Figure 1-figure supplement 1G-Original WB images/Fig. S1G JNK.tif]

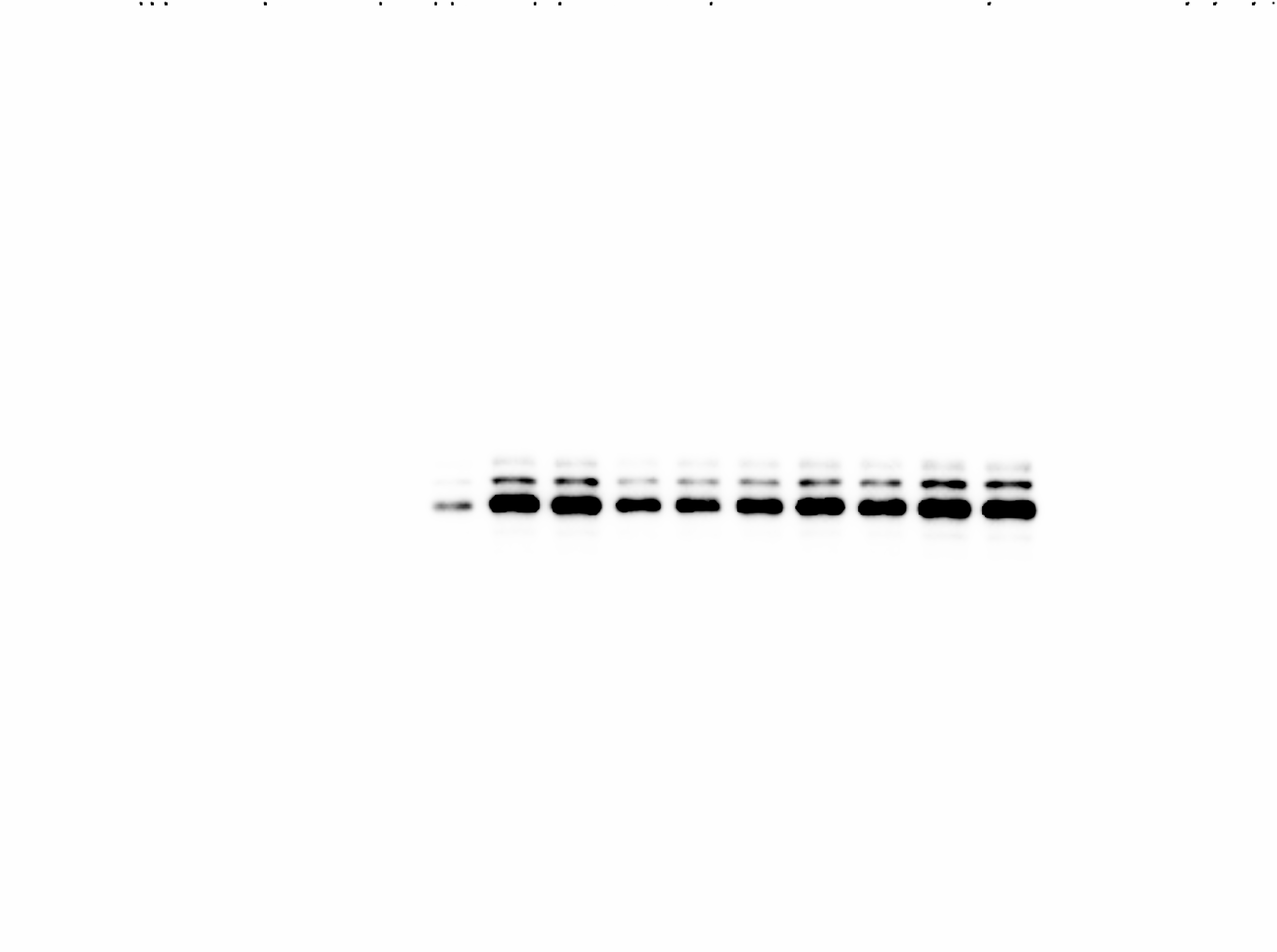

Supplement: Figure 1—figure supplement 1—source data 2. [file elife-68843-fig1-figsupp1-data2.zip › Figure 1-figure supplement 1G-Original WB images/Fig. S1G NOX4.tif]

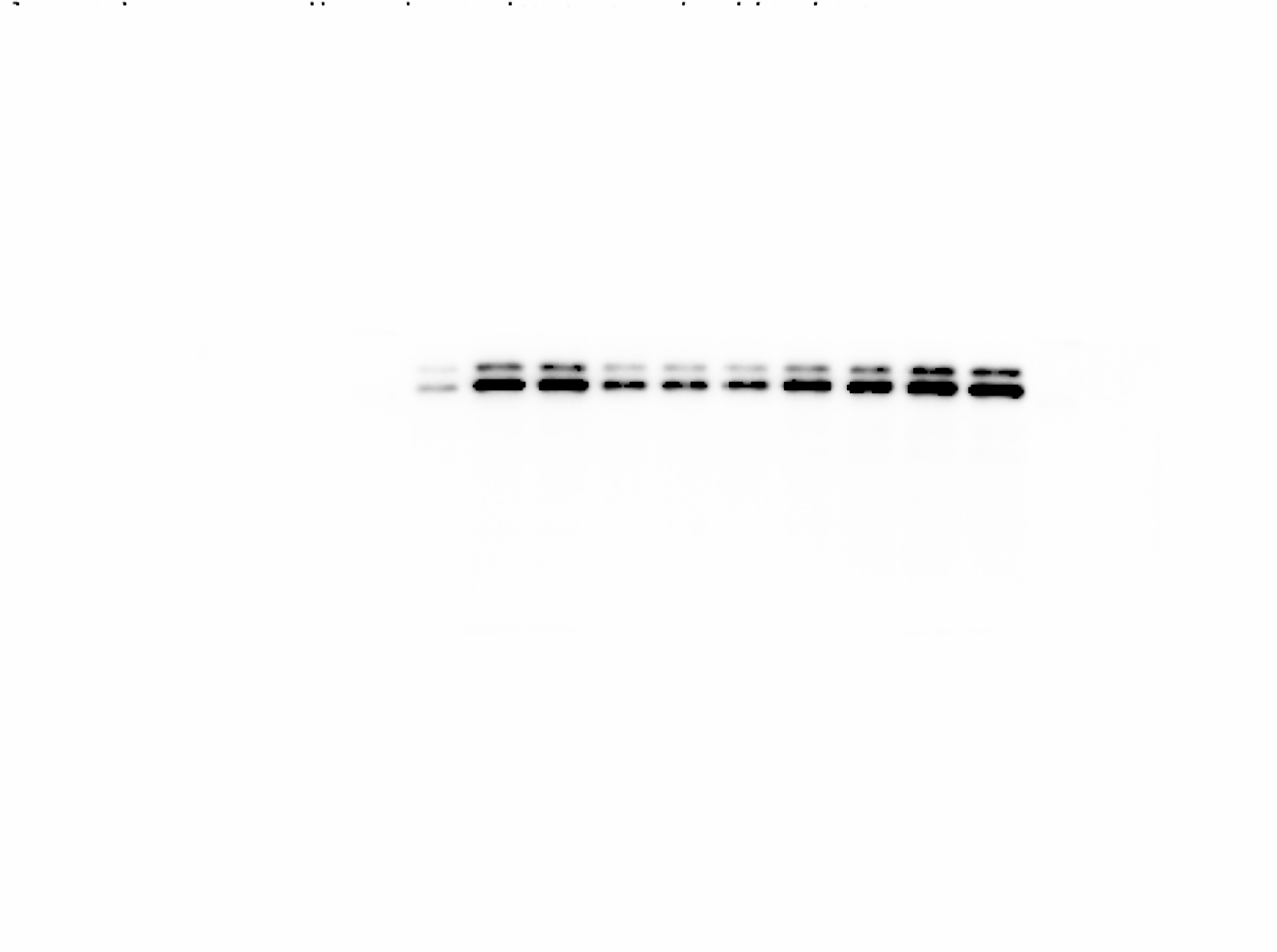

Supplement: Figure 1—figure supplement 1—source data 2. [file elife-68843-fig1-figsupp1-data2.zip › Figure 1-figure supplement 1G-Original WB images/Fig. S1G p-ERK.tif]

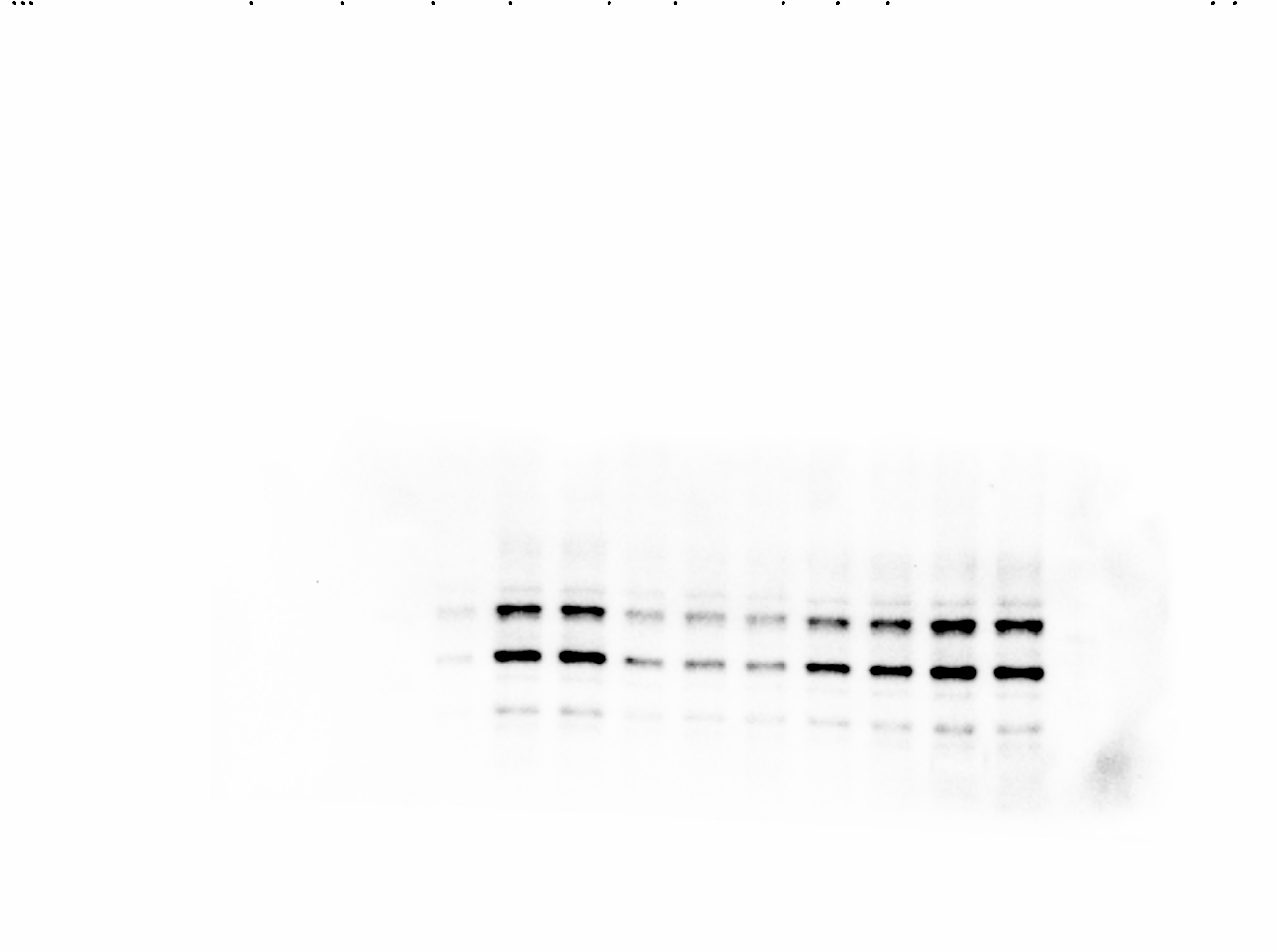

Supplement: Figure 1—figure supplement 1—source data 2. [file elife-68843-fig1-figsupp1-data2.zip › Figure 1-figure supplement 1G-Original WB images/Fig. S1G p-JNK.tif]

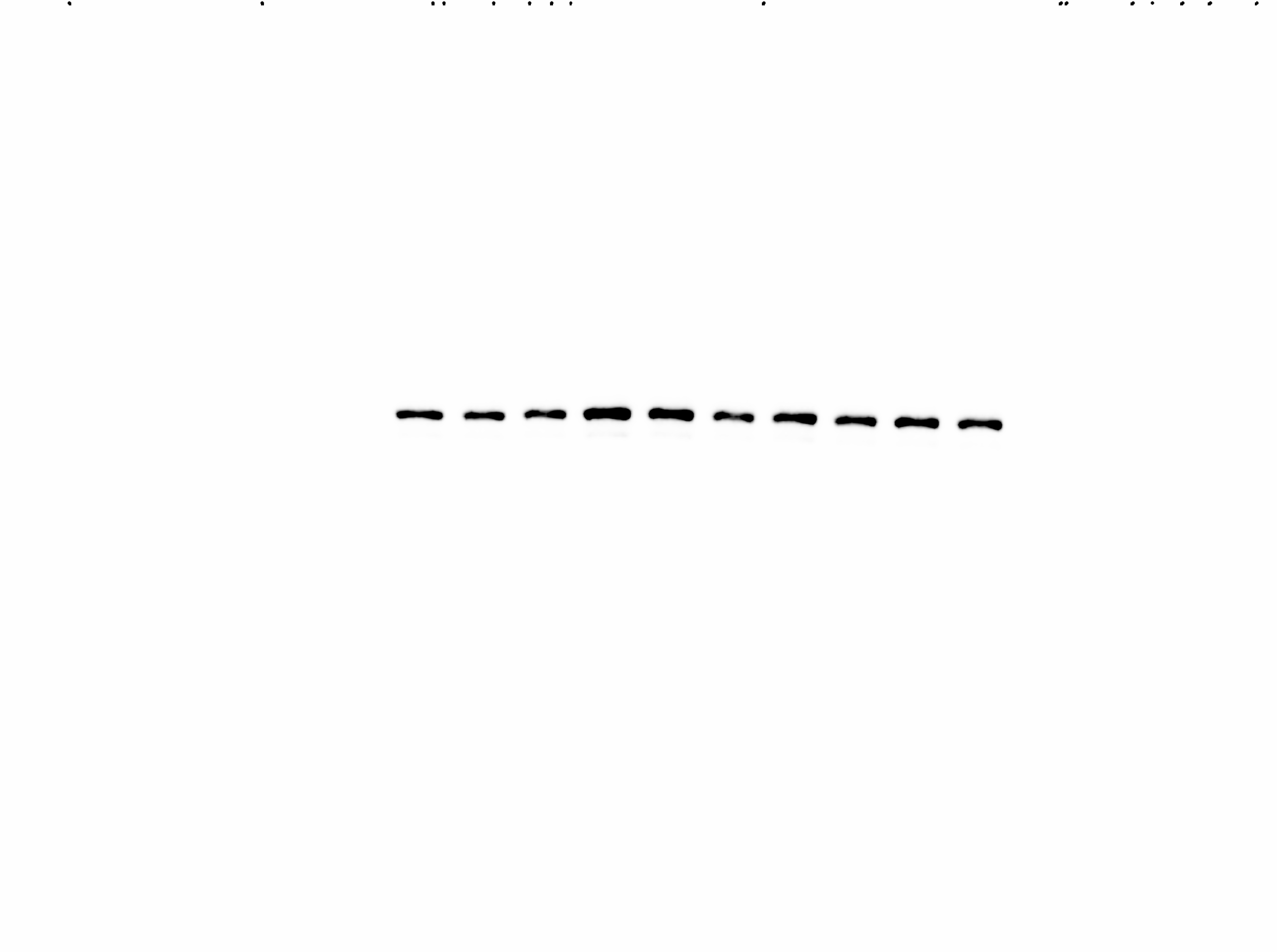

Supplement: Figure 1—figure supplement 1—source data 2. [file elife-68843-fig1-figsupp1-data2.zip › Figure 1-figure supplement 1G-Original WB images/Fig. S1G p-STAT3.tif]

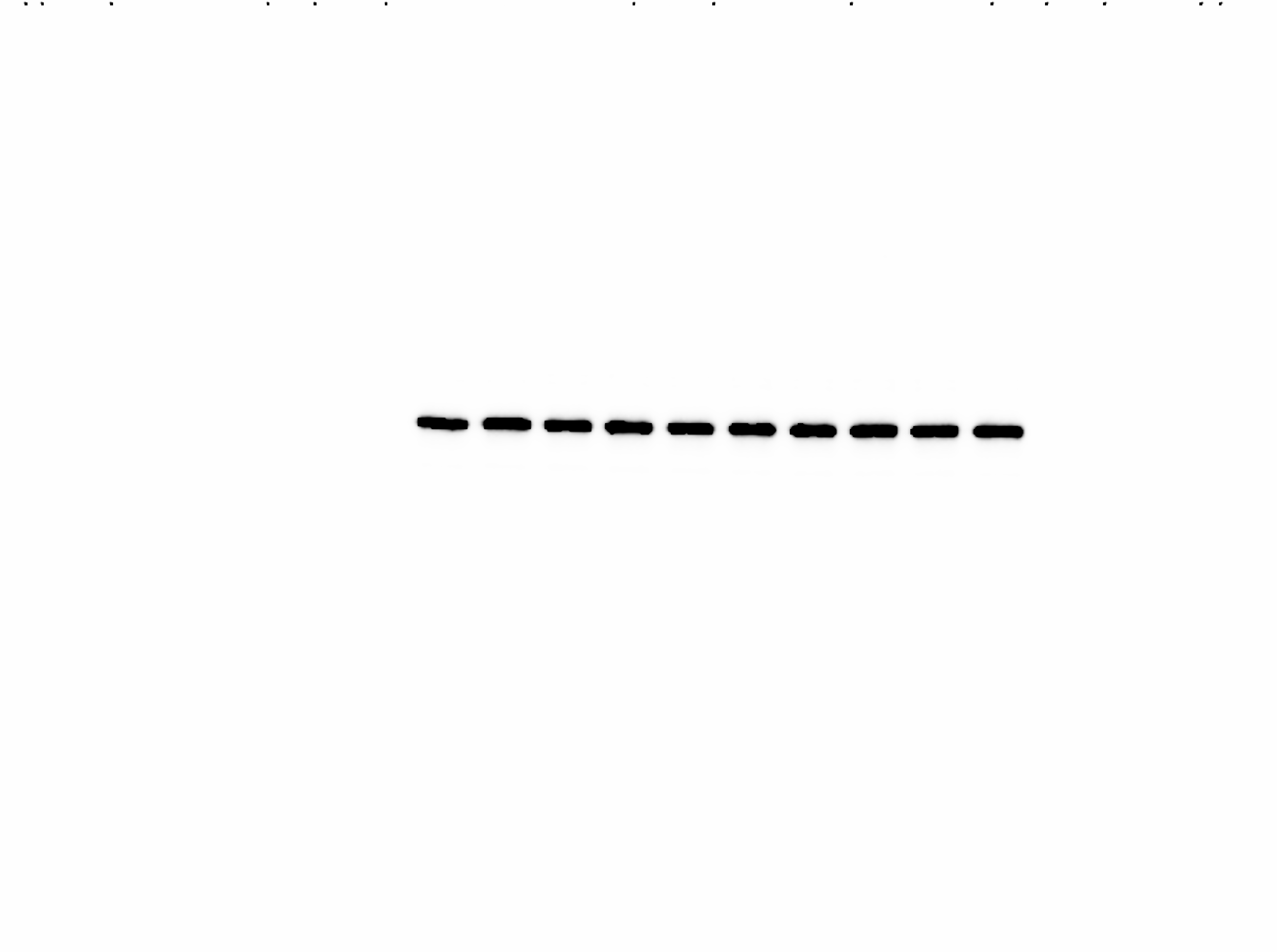

Supplement: Figure 1—figure supplement 1—source data 2. [file elife-68843-fig1-figsupp1-data2.zip › Figure 1-figure supplement 1G-Original WB images/Fig. S1G STAT3.tif]

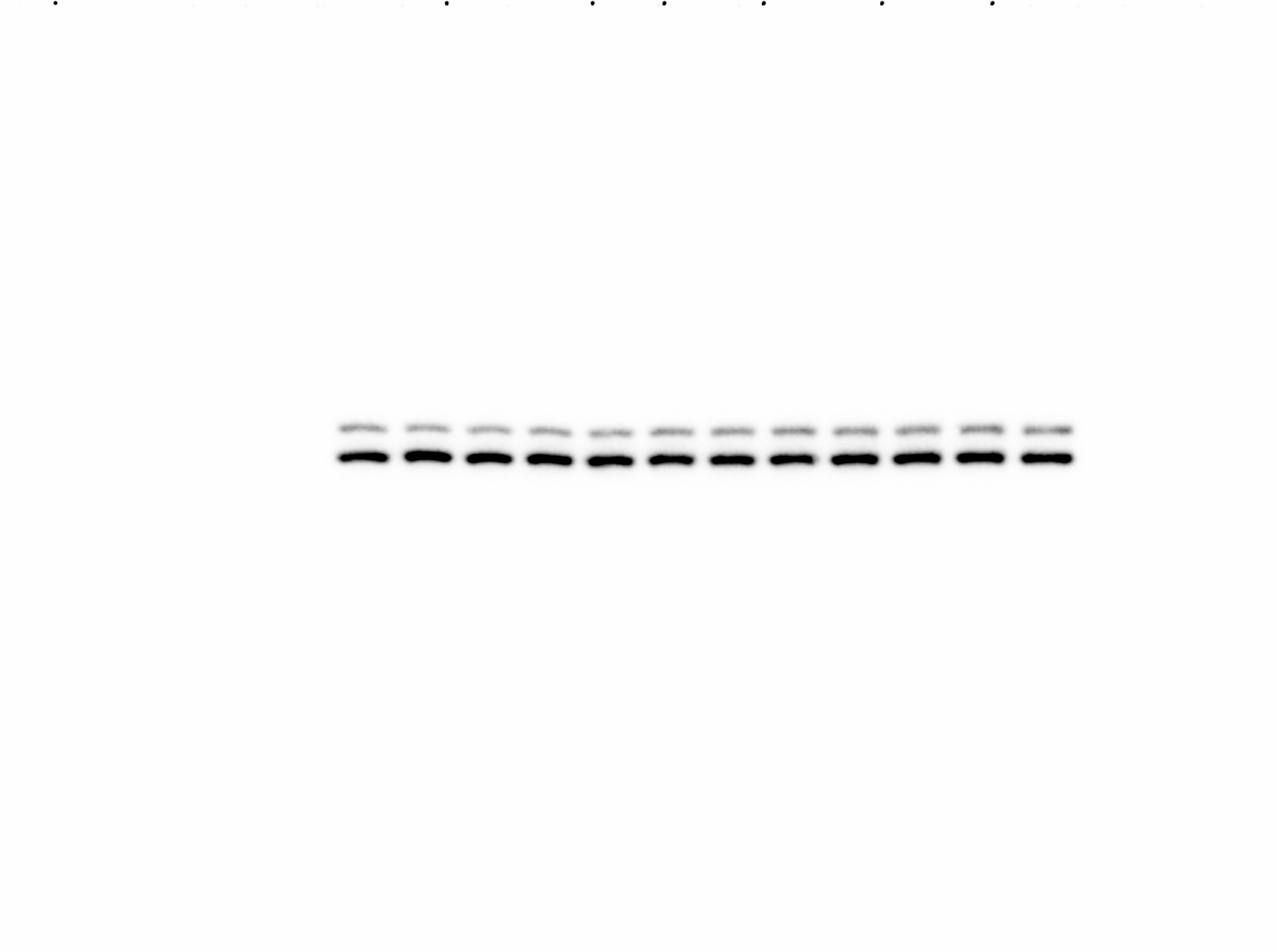

Supplement: Figure 2—source data 2. [file elife-68843-fig2-data2.zip › Figure 2E-Original WB images/Fig.2E ERK.tif]

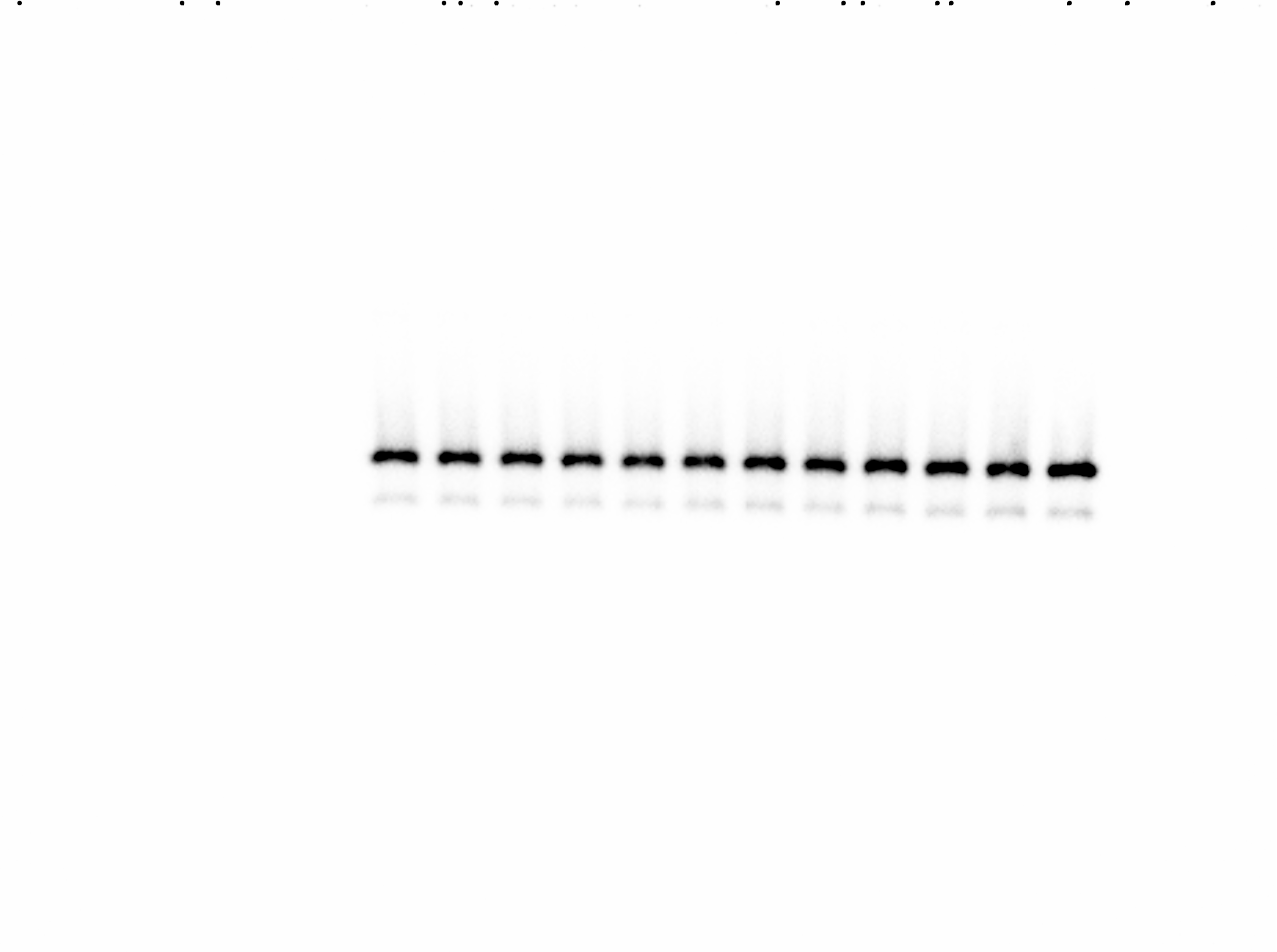

Supplement: Figure 2—source data 2. [file elife-68843-fig2-data2.zip › Figure 2E-Original WB images/Fig.2E JNK.tif]

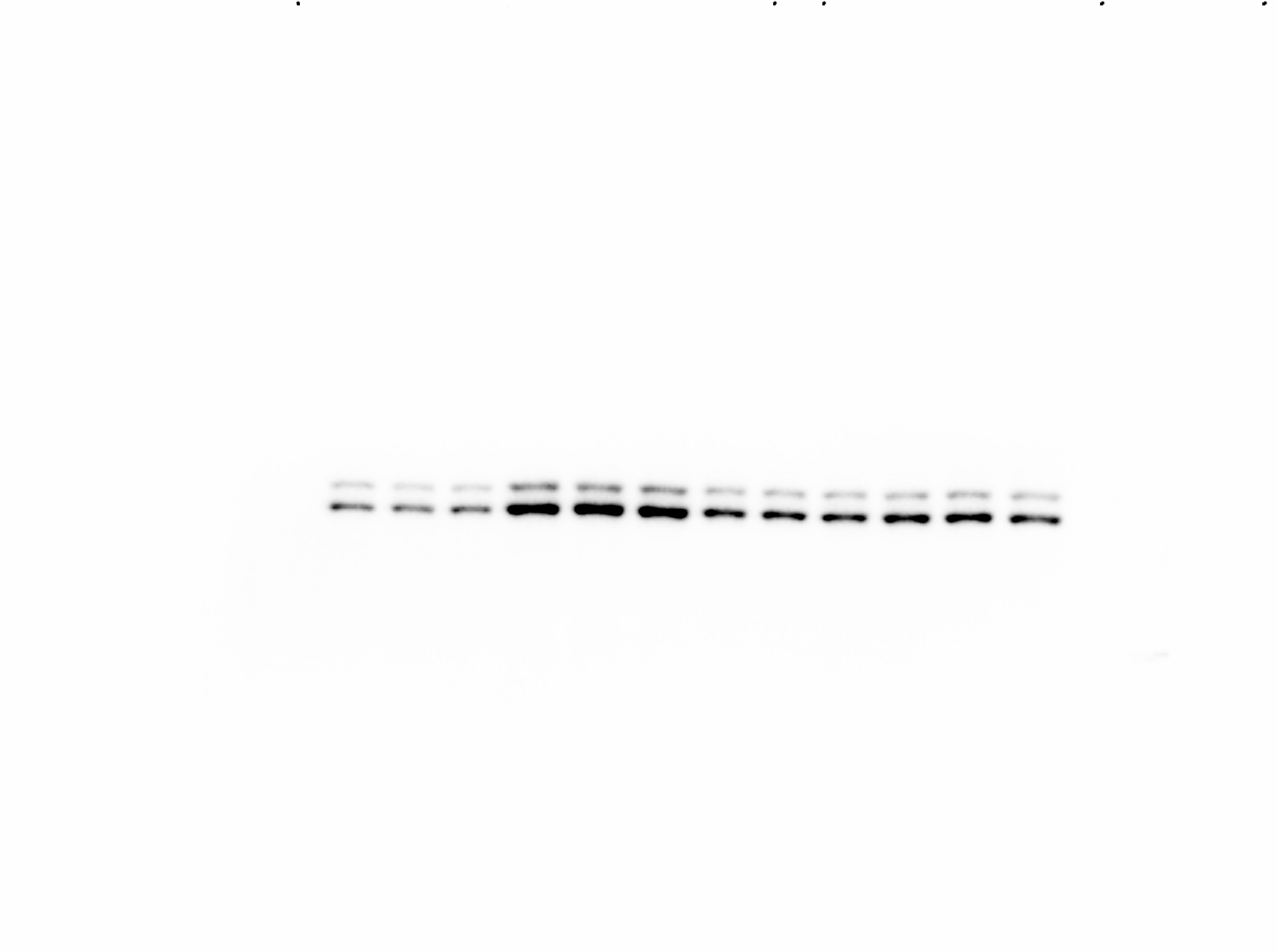

Supplement: Figure 2—source data 2. [file elife-68843-fig2-data2.zip › Figure 2E-Original WB images/Fig.2E p-ERK.tif]

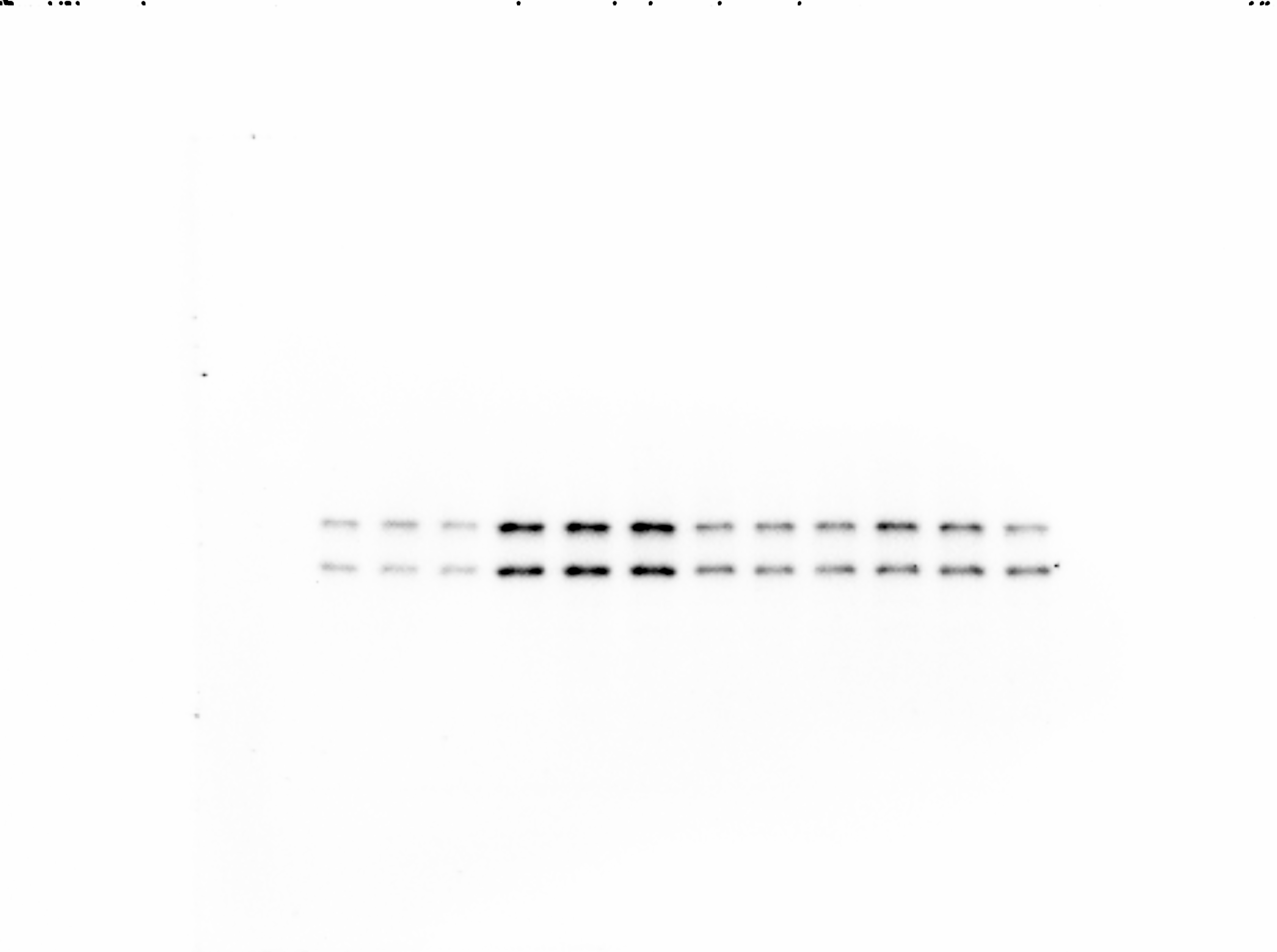

Supplement: Figure 2—source data 2. [file elife-68843-fig2-data2.zip › Figure 2E-Original WB images/Fig.2E p-JNK.tif]

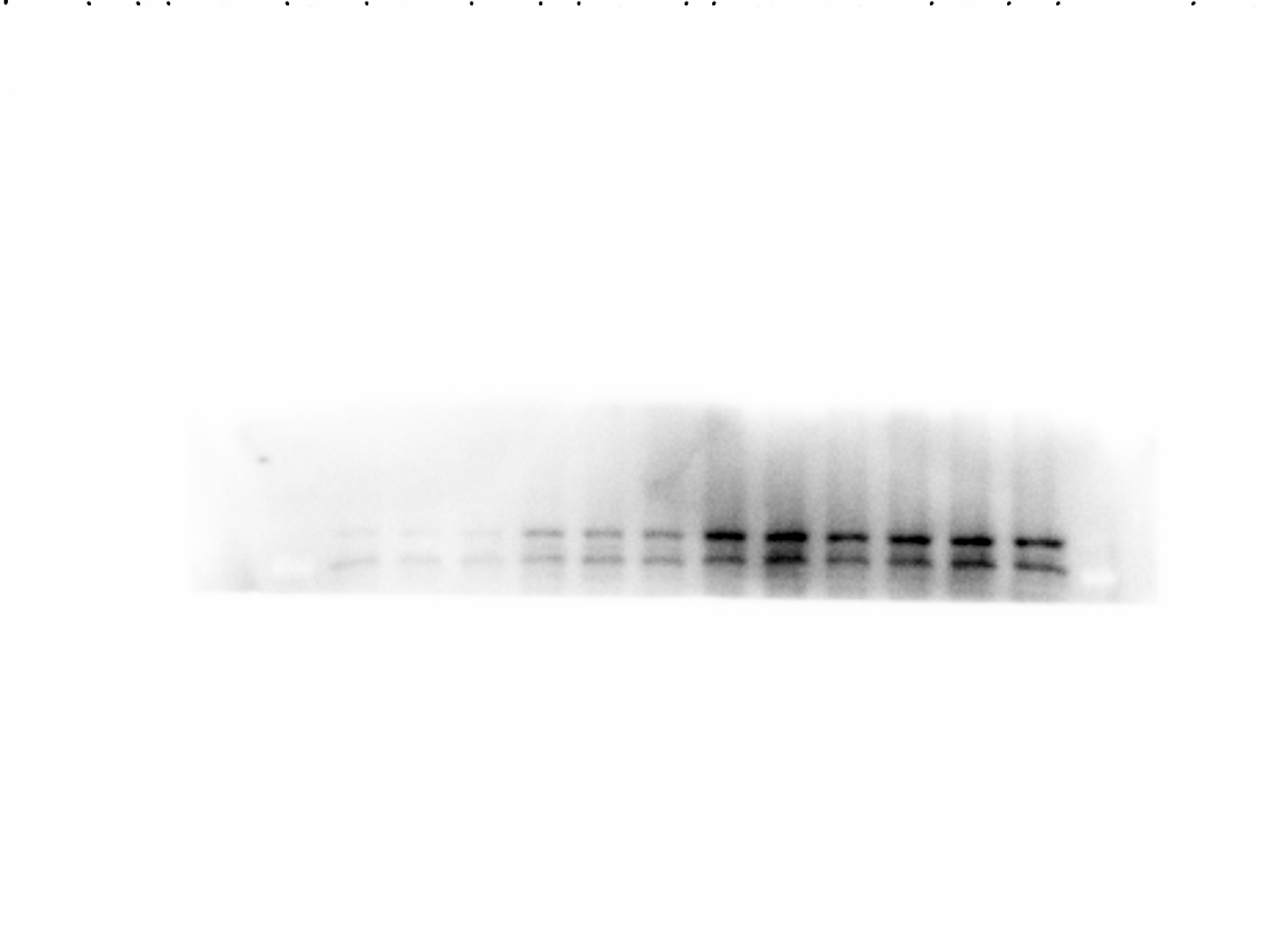

Supplement: Figure 2—source data 2. [file elife-68843-fig2-data2.zip › Figure 2E-Original WB images/Fig.2E p-STAT3.tif]

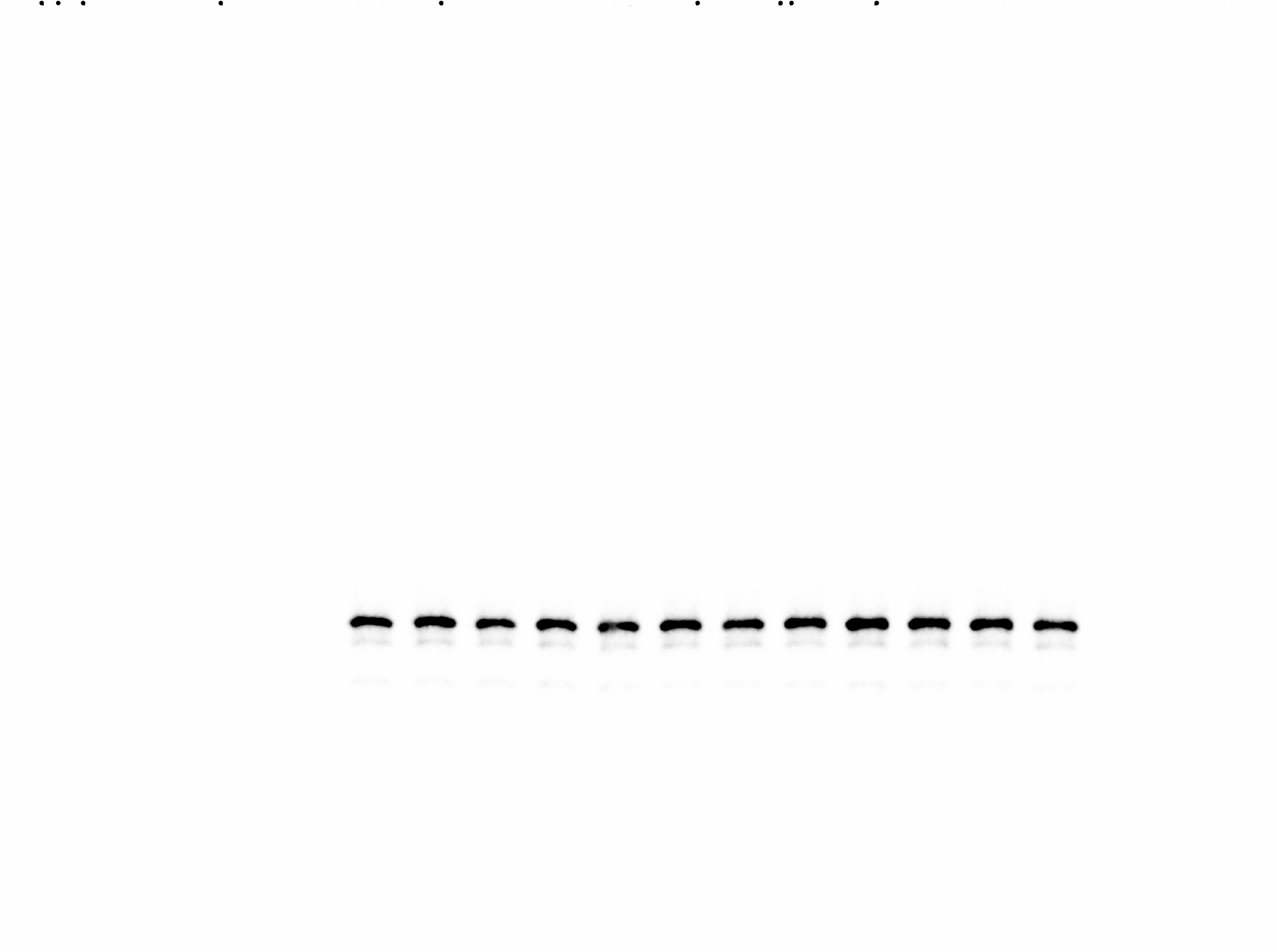

Supplement: Figure 2—source data 2. [file elife-68843-fig2-data2.zip › Figure 2E-Original WB images/Fig.2E STAT3.tif]

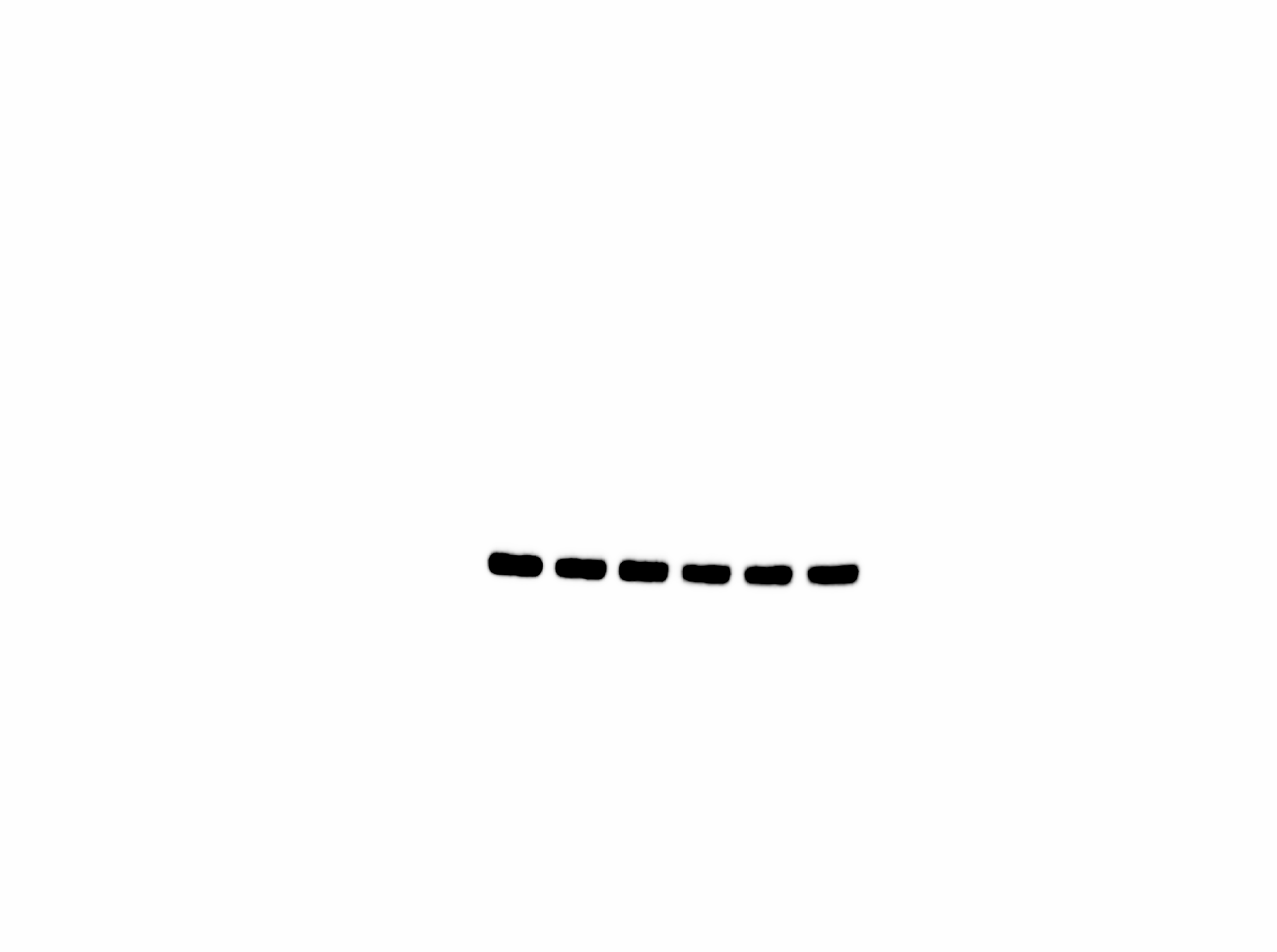

Supplement: Figure 2—source data 2. [file elife-68843-fig2-data2.zip › Figure 2H-Original WB images/Fig.2H GAPDH.tif]

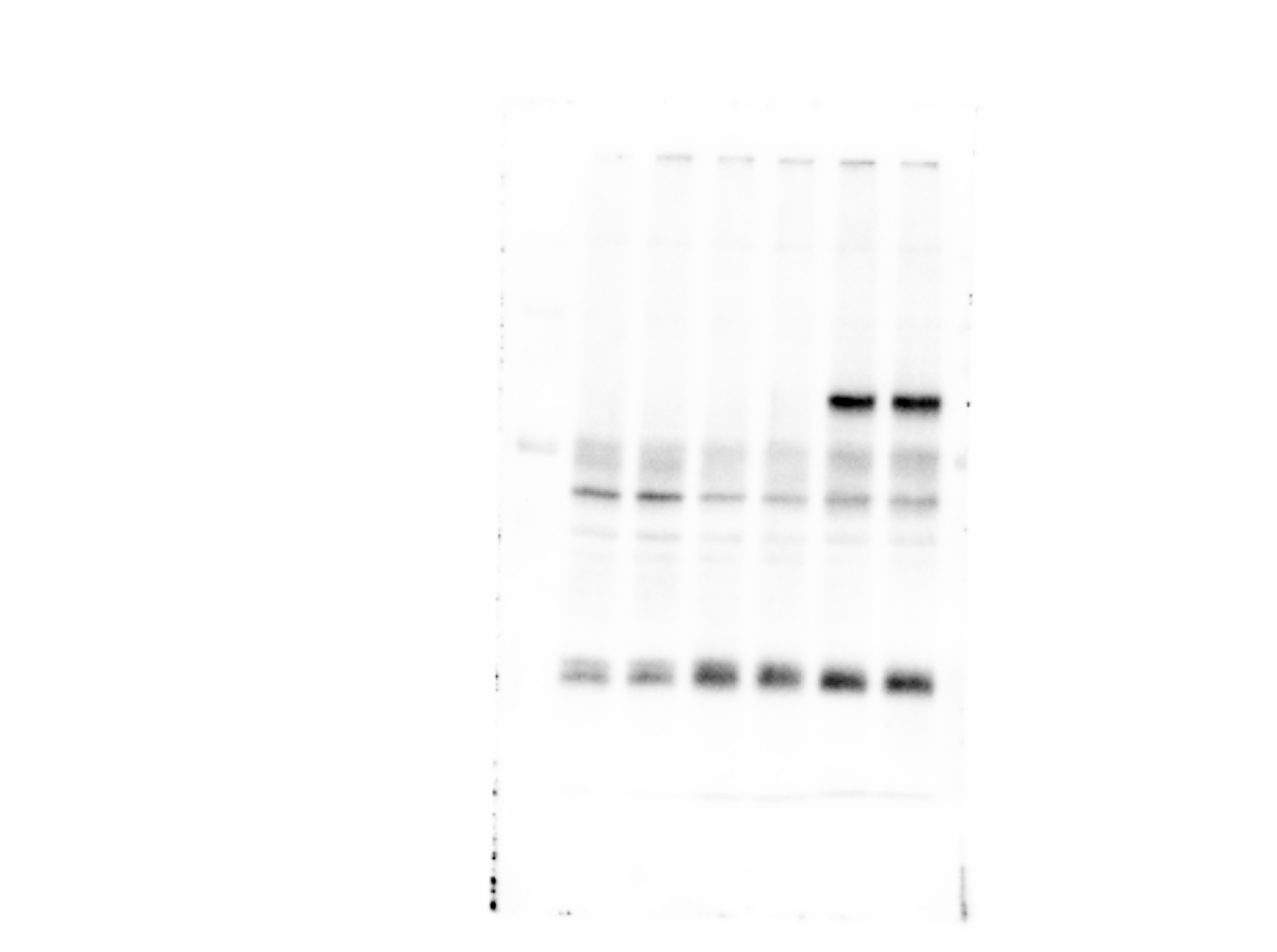

Supplement: Figure 2—source data 2. [file elife-68843-fig2-data2.zip › Figure 2H-Original WB images/Fig.2H HyperIL6.tif]

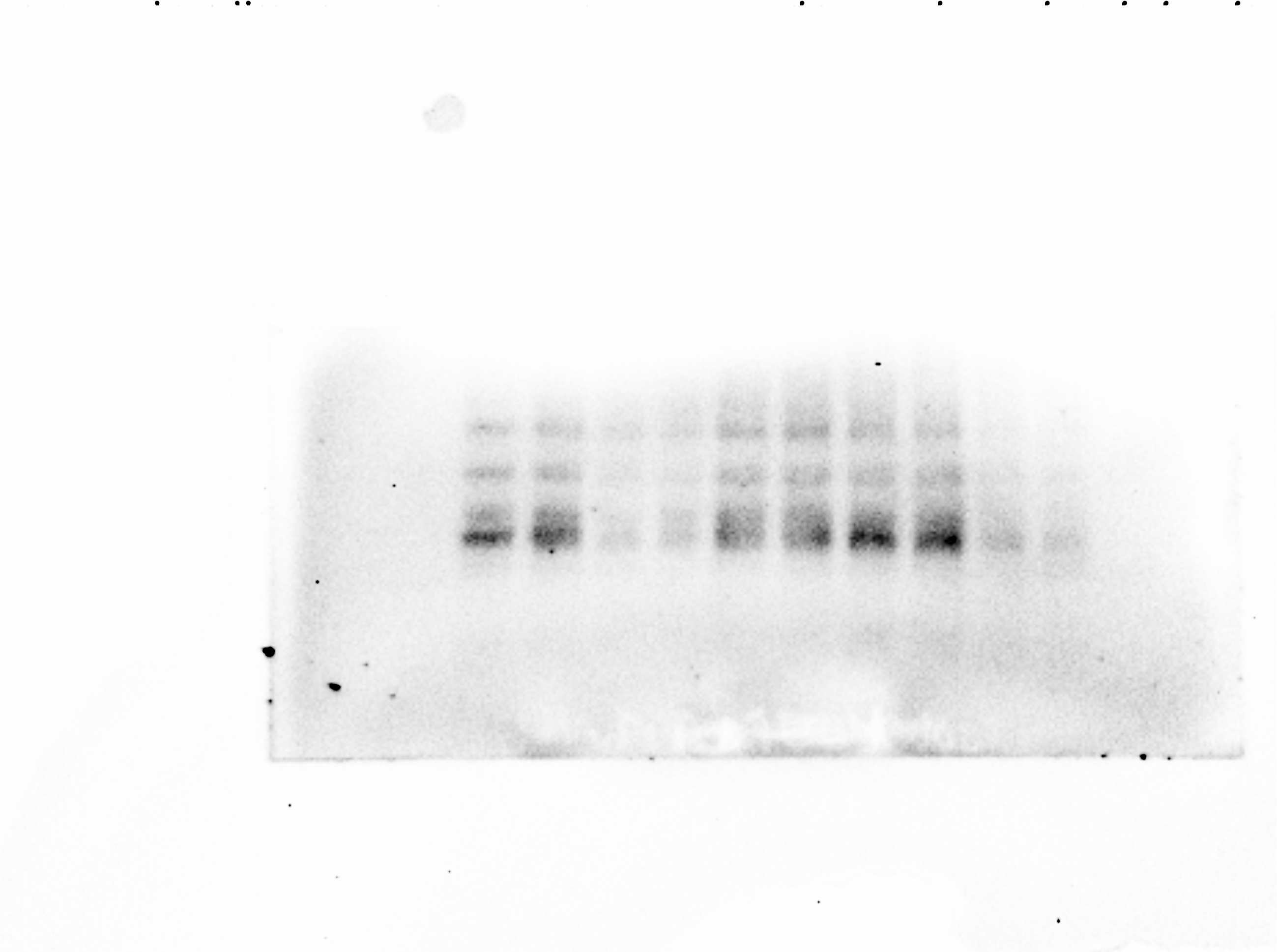

Supplement: Figure 2—source data 2. [file elife-68843-fig2-data2.zip › Figure 2K-Original WB images/Fig.2K Cyclin D1.tif]

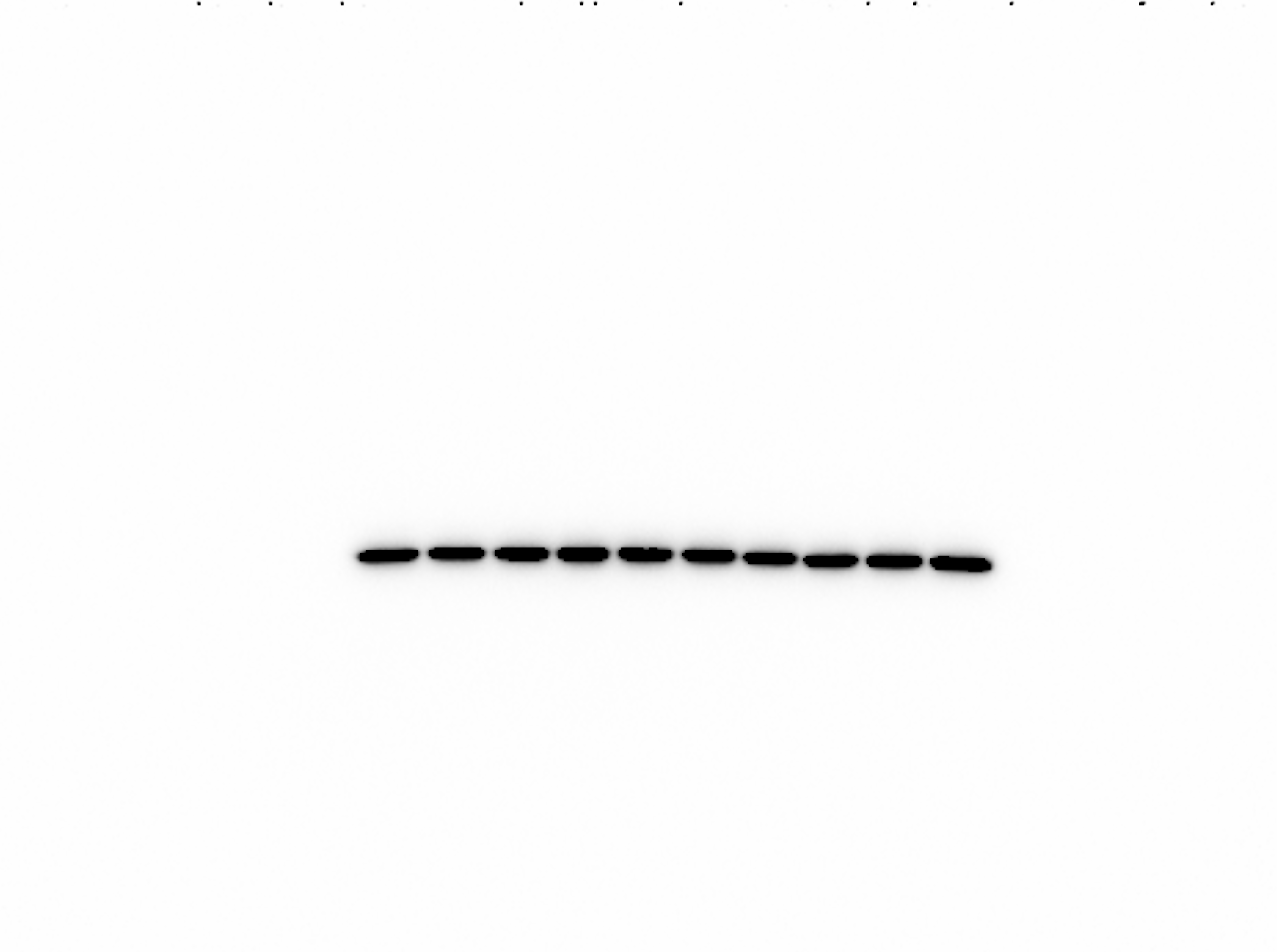

Supplement: Figure 2—source data 2. [file elife-68843-fig2-data2.zip › Figure 2K-Original WB images/Fig.2K GAPDH.tif]

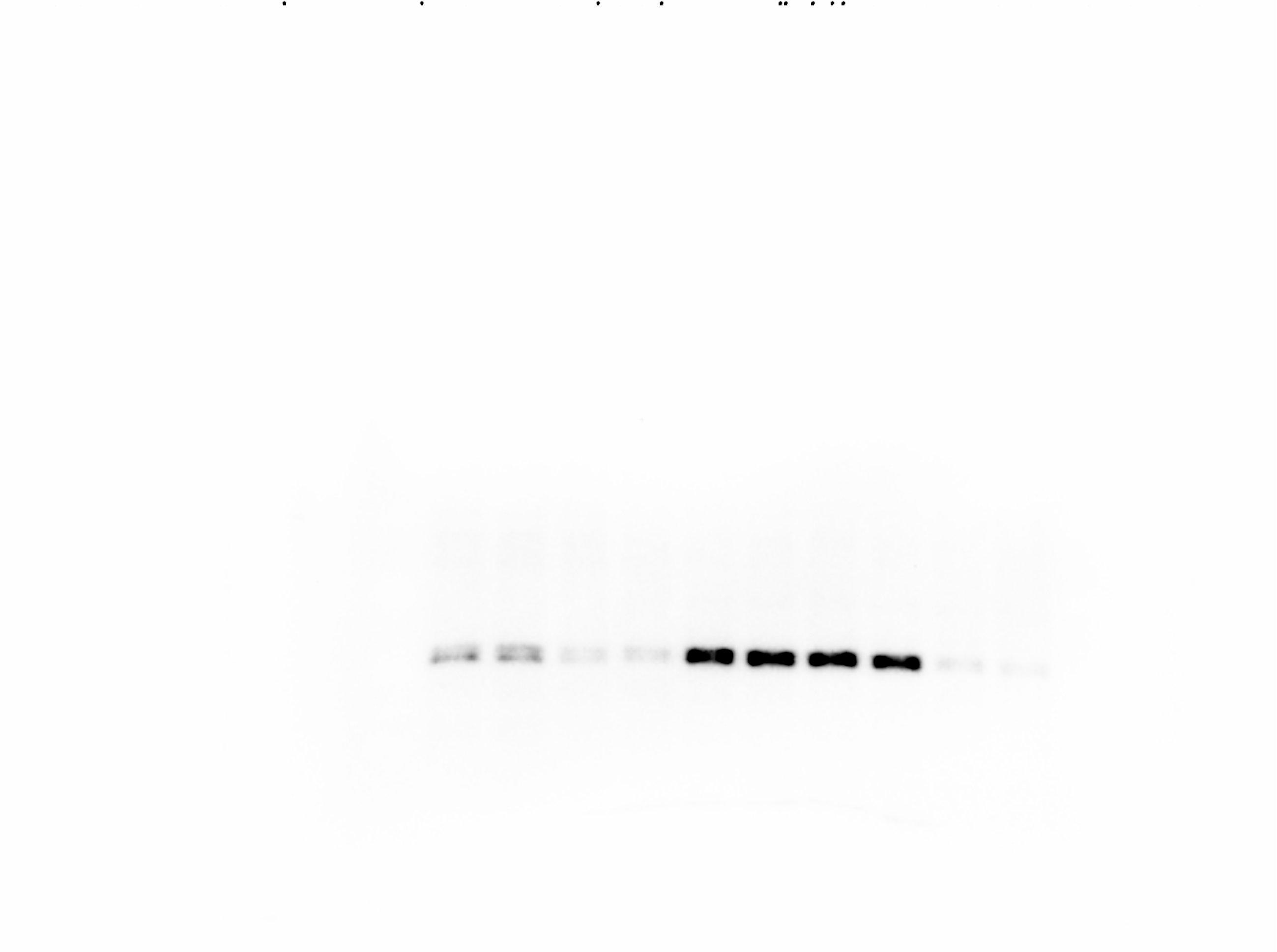

Supplement: Figure 2—source data 2. [file elife-68843-fig2-data2.zip › Figure 2K-Original WB images/Fig.2K PCNA.tif]

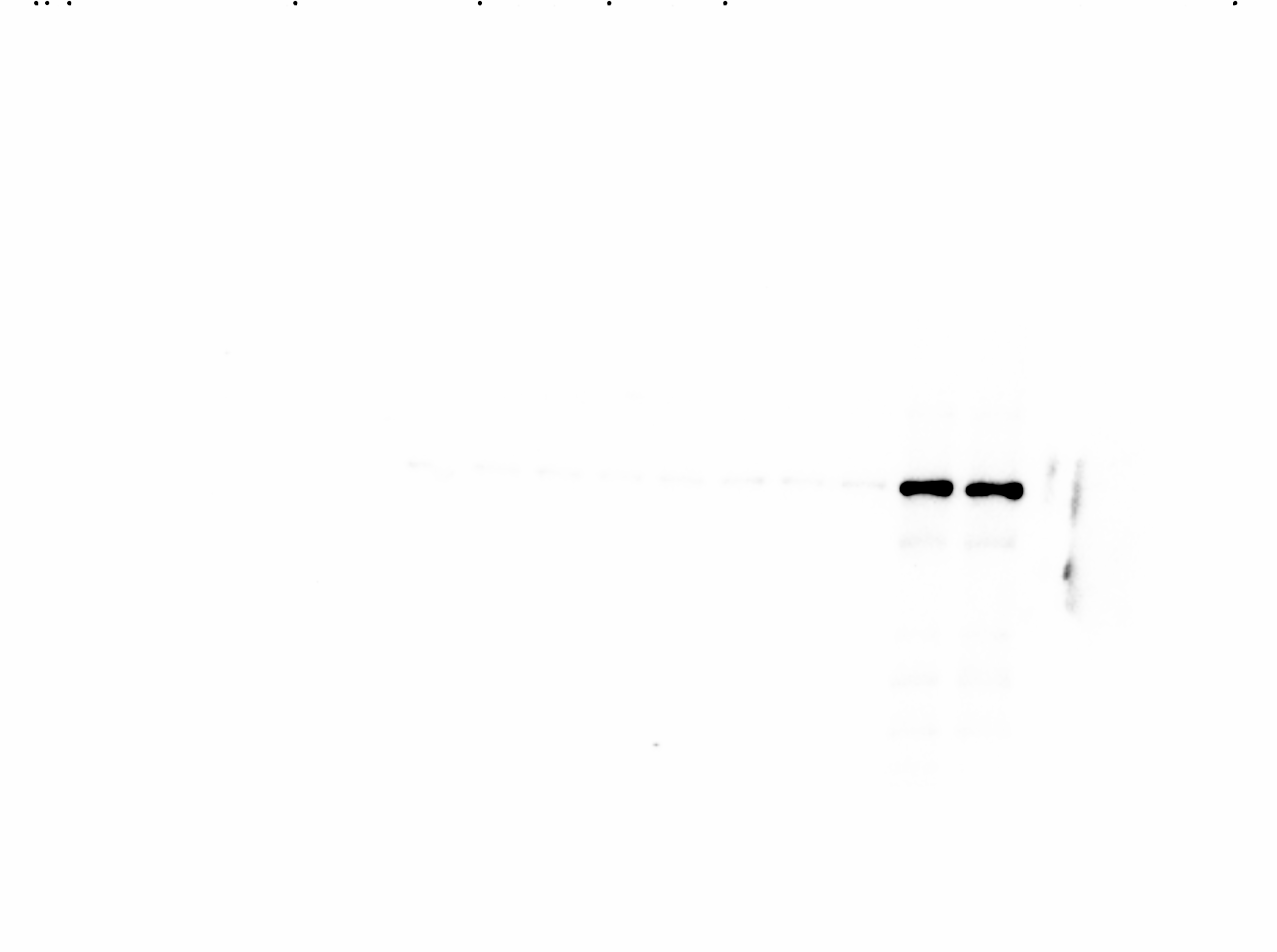

Supplement: Figure 2—source data 2. [file elife-68843-fig2-data2.zip › Figure 2K-Original WB images/Fig.2K sIL6ST.tif]

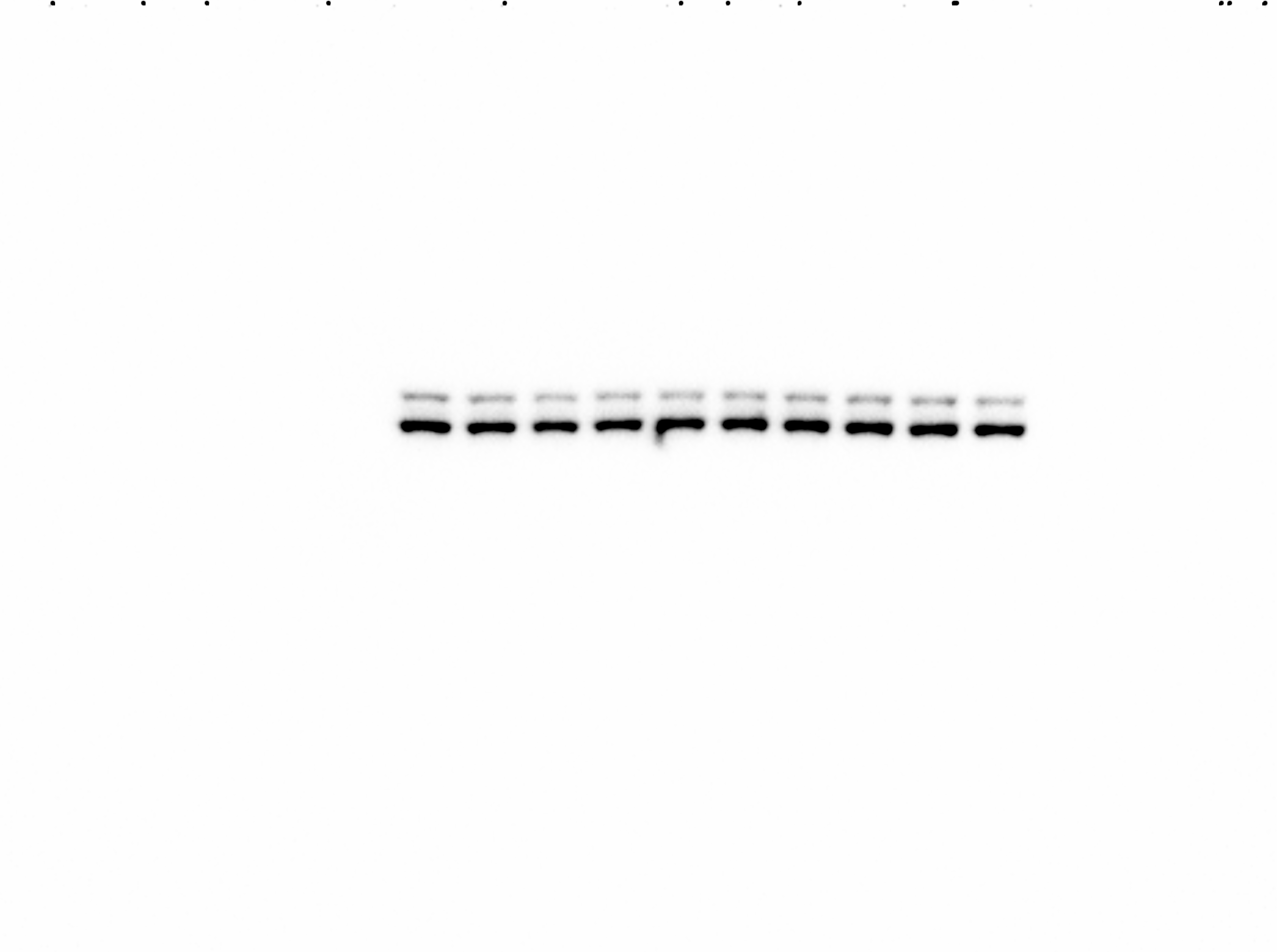

Supplement: Figure 2—source data 2. [file elife-68843-fig2-data2.zip › Figure 2L-Original WB images/Fig. 2L ERK.tif]

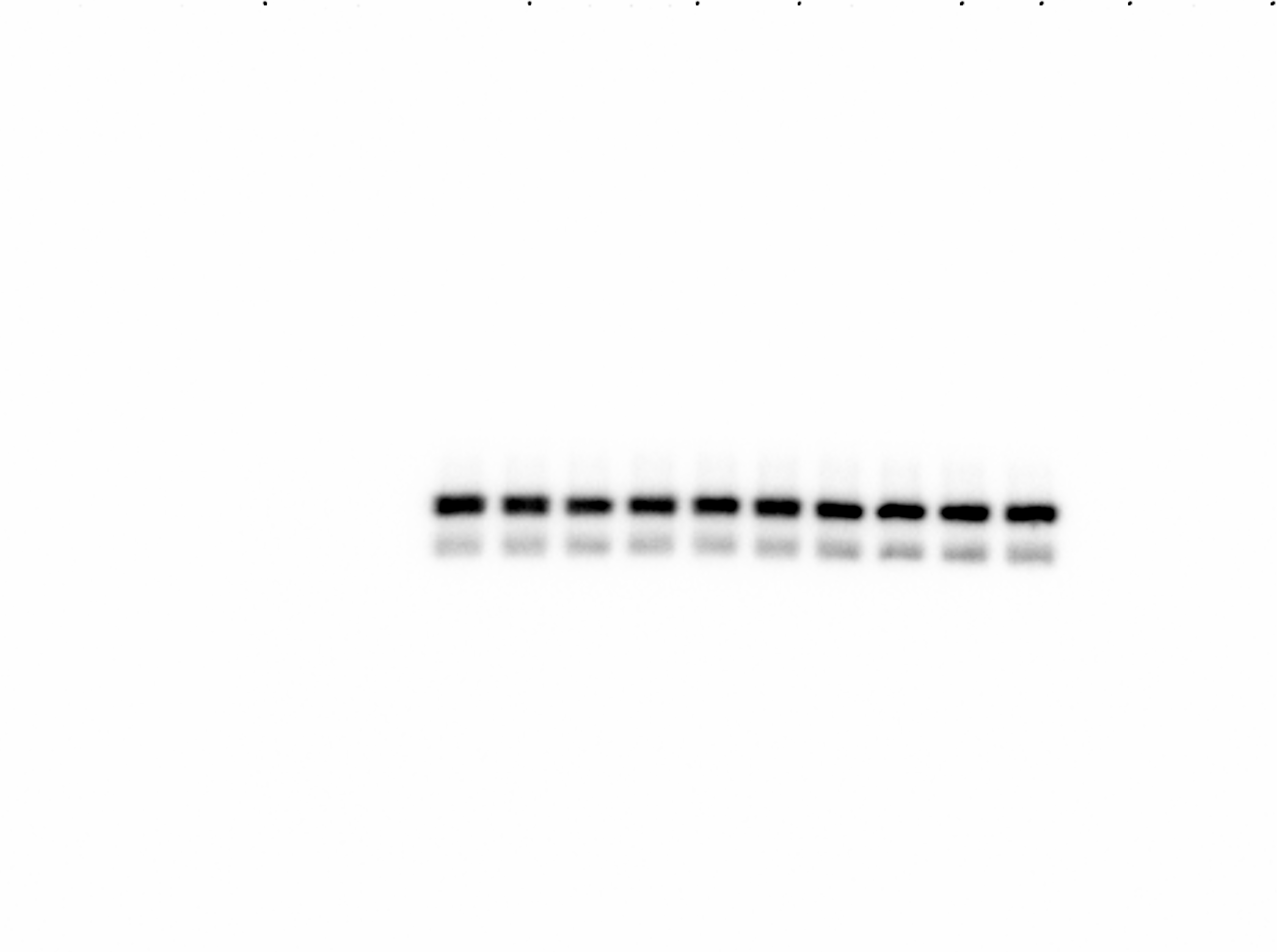

Supplement: Figure 2—source data 2. [file elife-68843-fig2-data2.zip › Figure 2L-Original WB images/Fig. 2L JNK.tif]

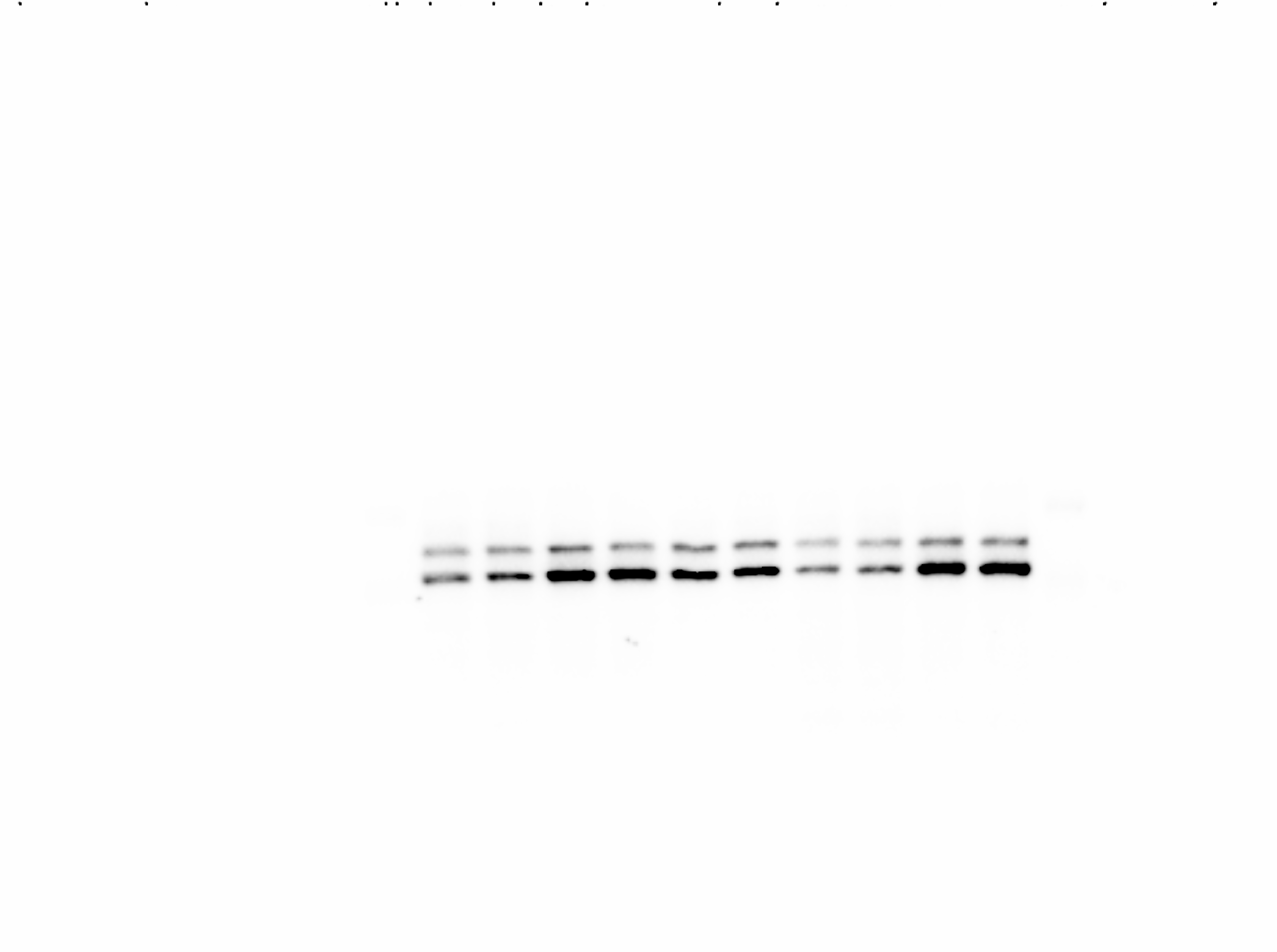

Supplement: Figure 2—source data 2. [file elife-68843-fig2-data2.zip › Figure 2L-Original WB images/Fig. 2L p-ERK.tif]

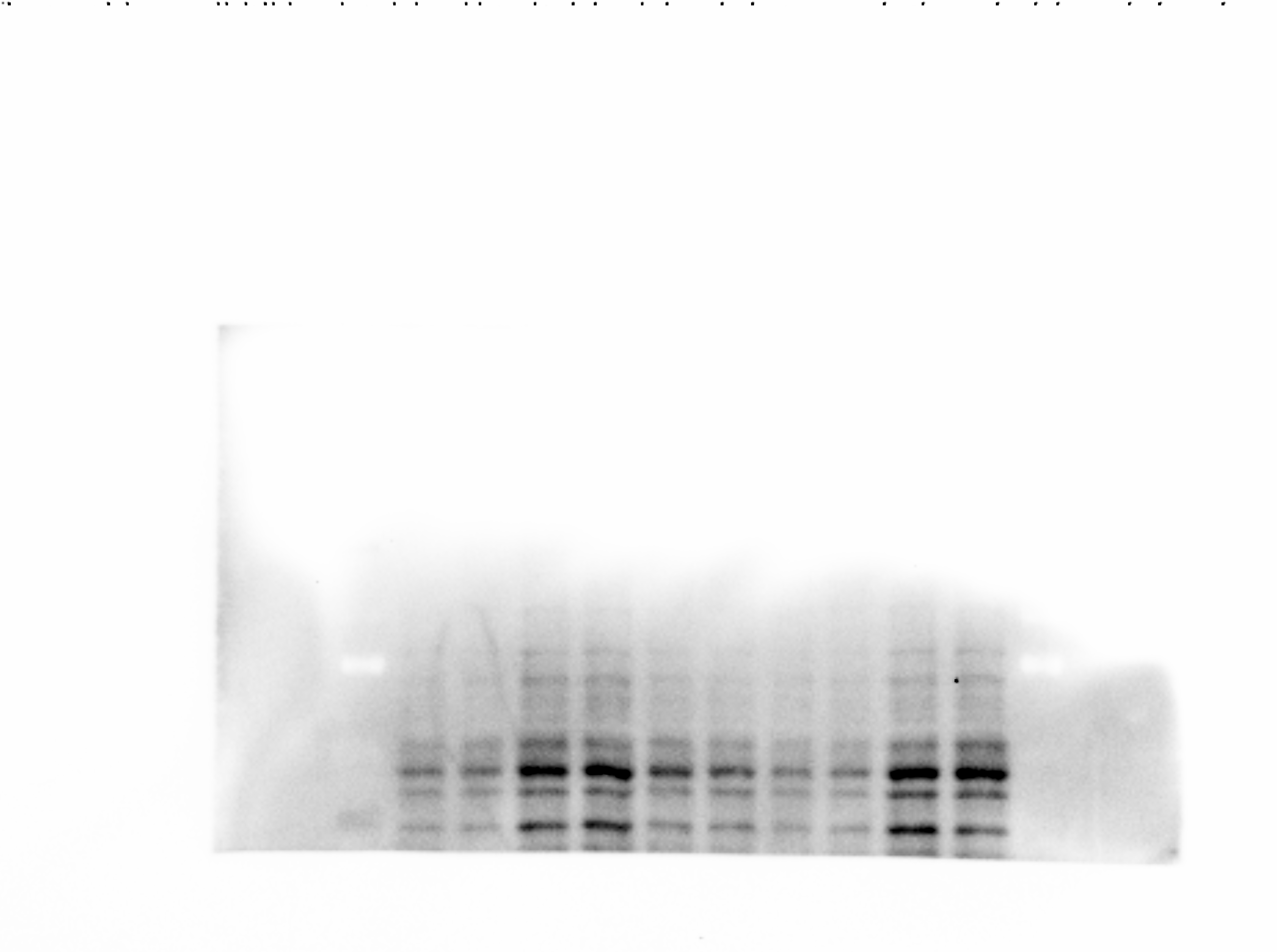

Supplement: Figure 2—source data 2. [file elife-68843-fig2-data2.zip › Figure 2L-Original WB images/Fig. 2L p-JNK.tif]

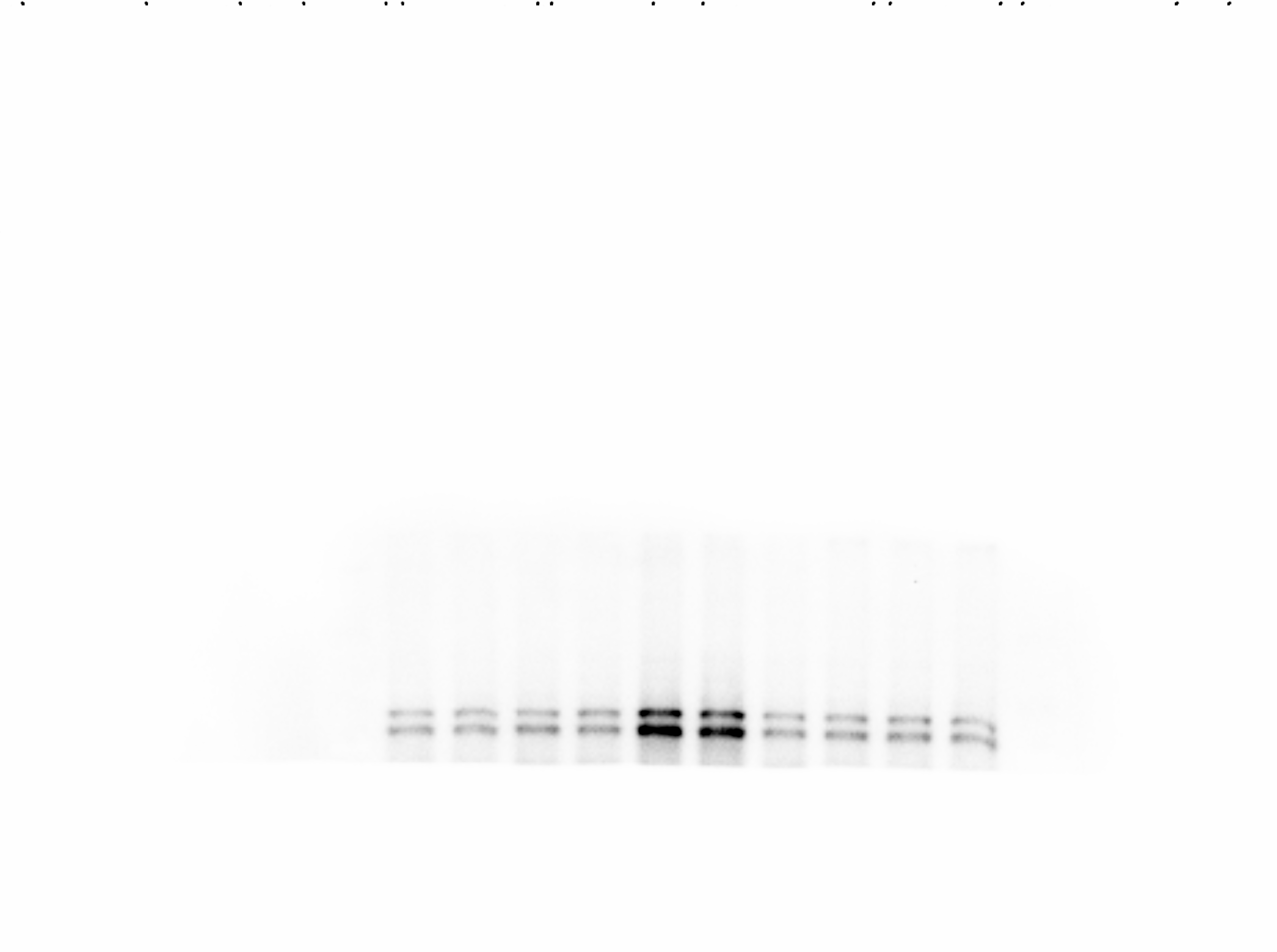

Supplement: Figure 2—source data 2. [file elife-68843-fig2-data2.zip › Figure 2L-Original WB images/Fig. 2L p-STAT3.tif]

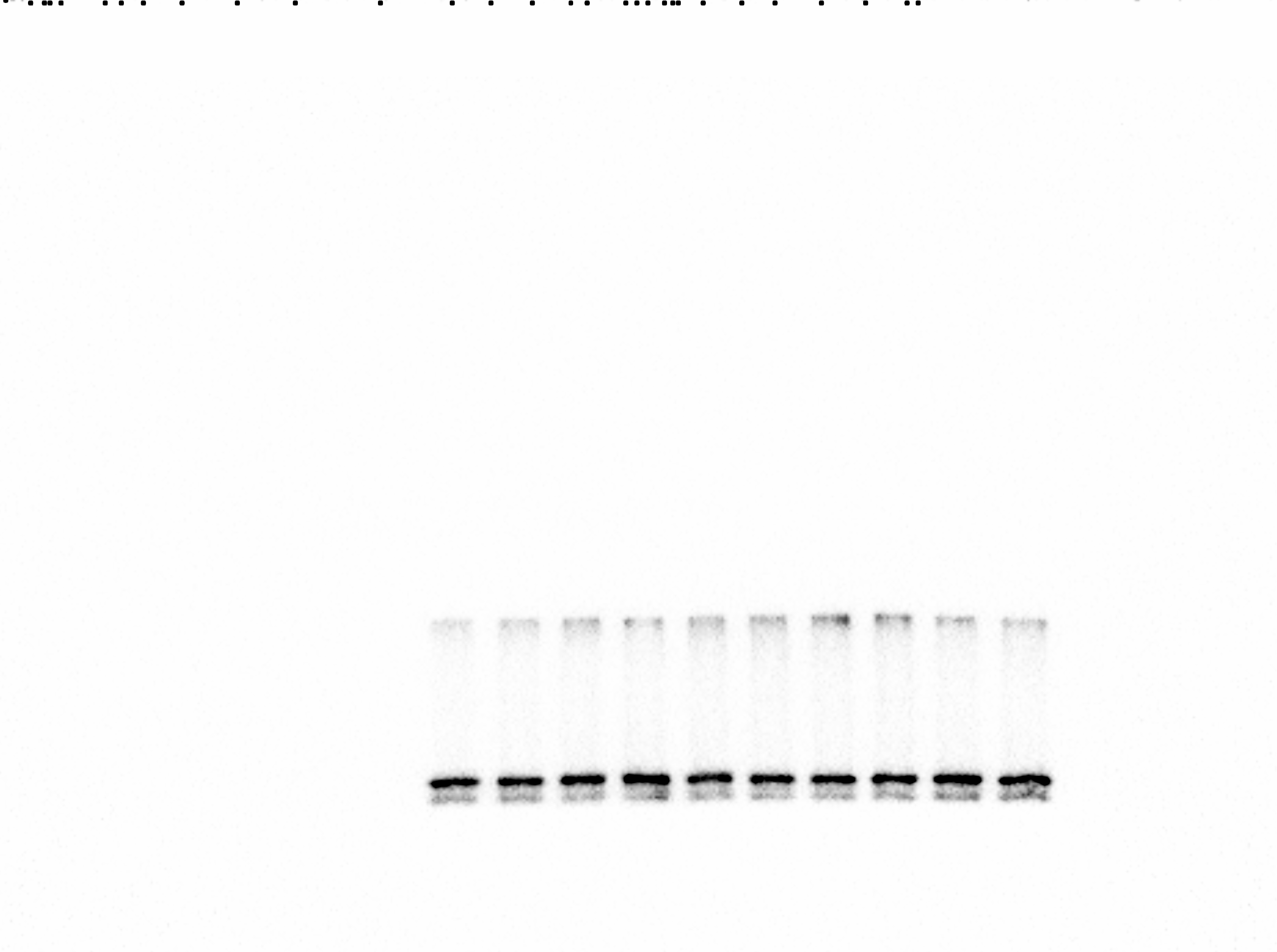

Supplement: Figure 2—source data 2. [file elife-68843-fig2-data2.zip › Figure 2L-Original WB images/Fig. 2L STAT3.tif]

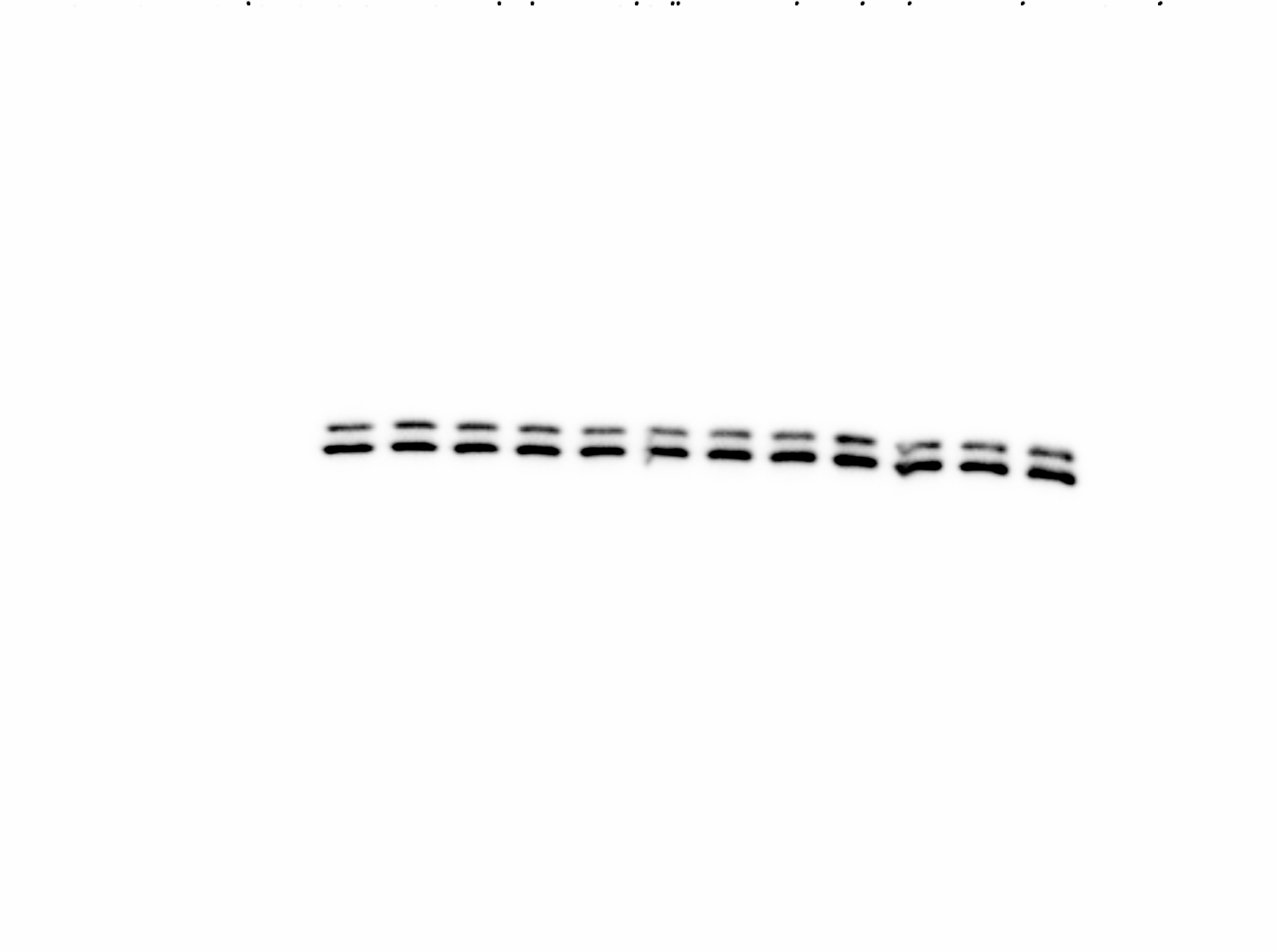

Supplement: Figure 3—source data 2. [file elife-68843-fig3-data2.zip › Figure 3D-Original WB images/Fig.3D ERK.tif]

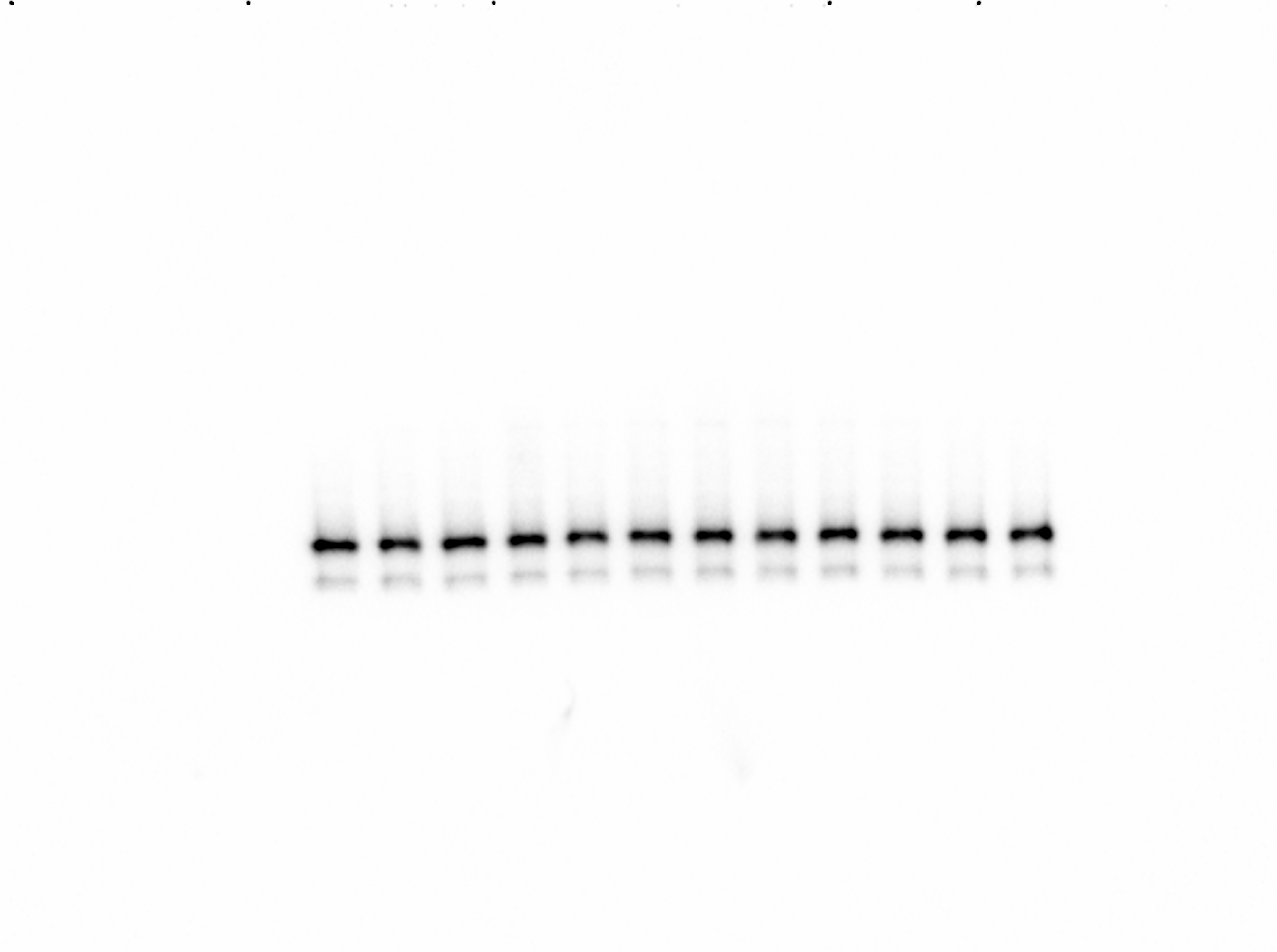

Supplement: Figure 3—source data 2. [file elife-68843-fig3-data2.zip › Figure 3D-Original WB images/Fig.3D JNK.tif]

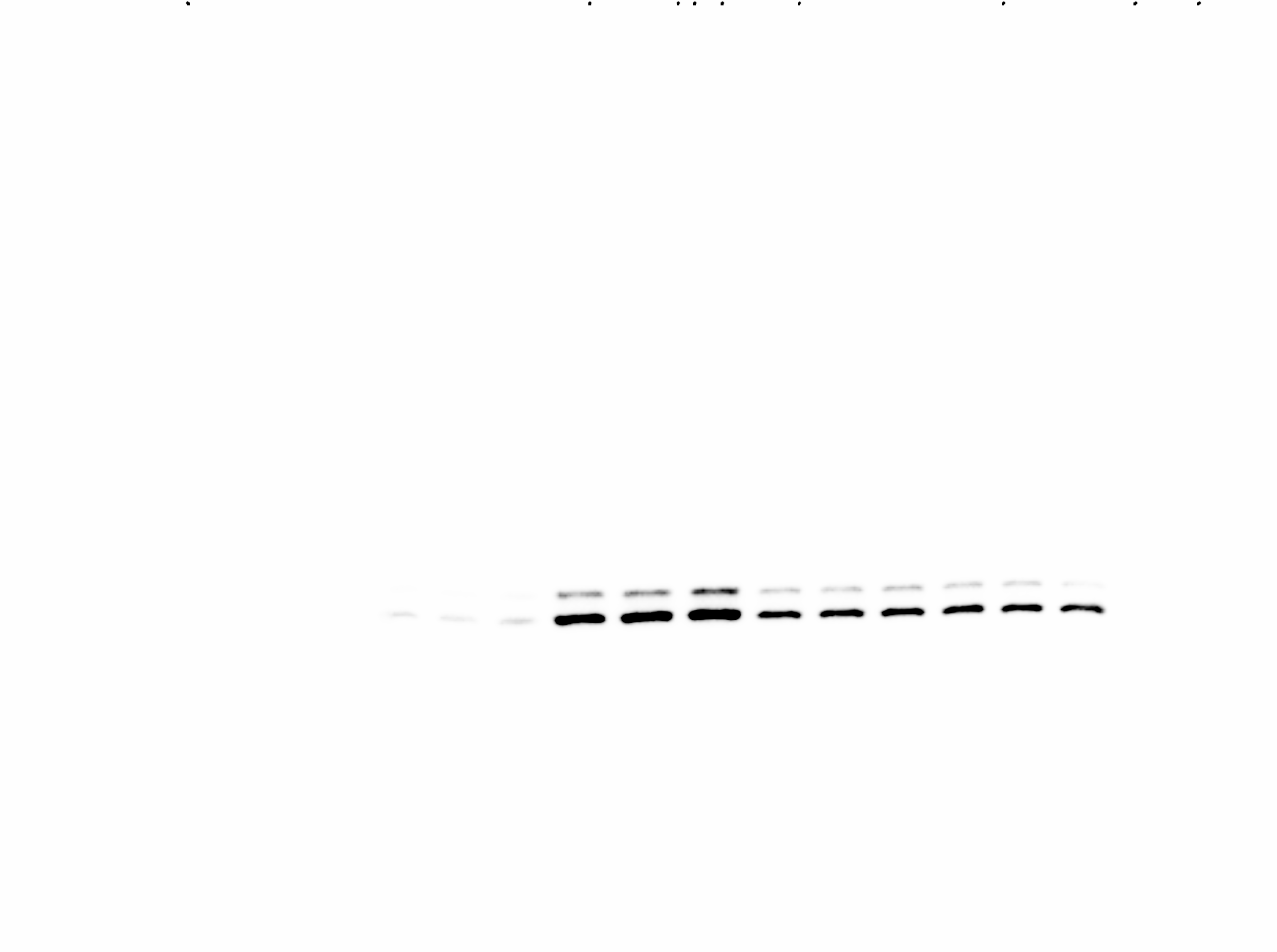

Supplement: Figure 3—source data 2. [file elife-68843-fig3-data2.zip › Figure 3D-Original WB images/Fig.3D p-ERK.tif]

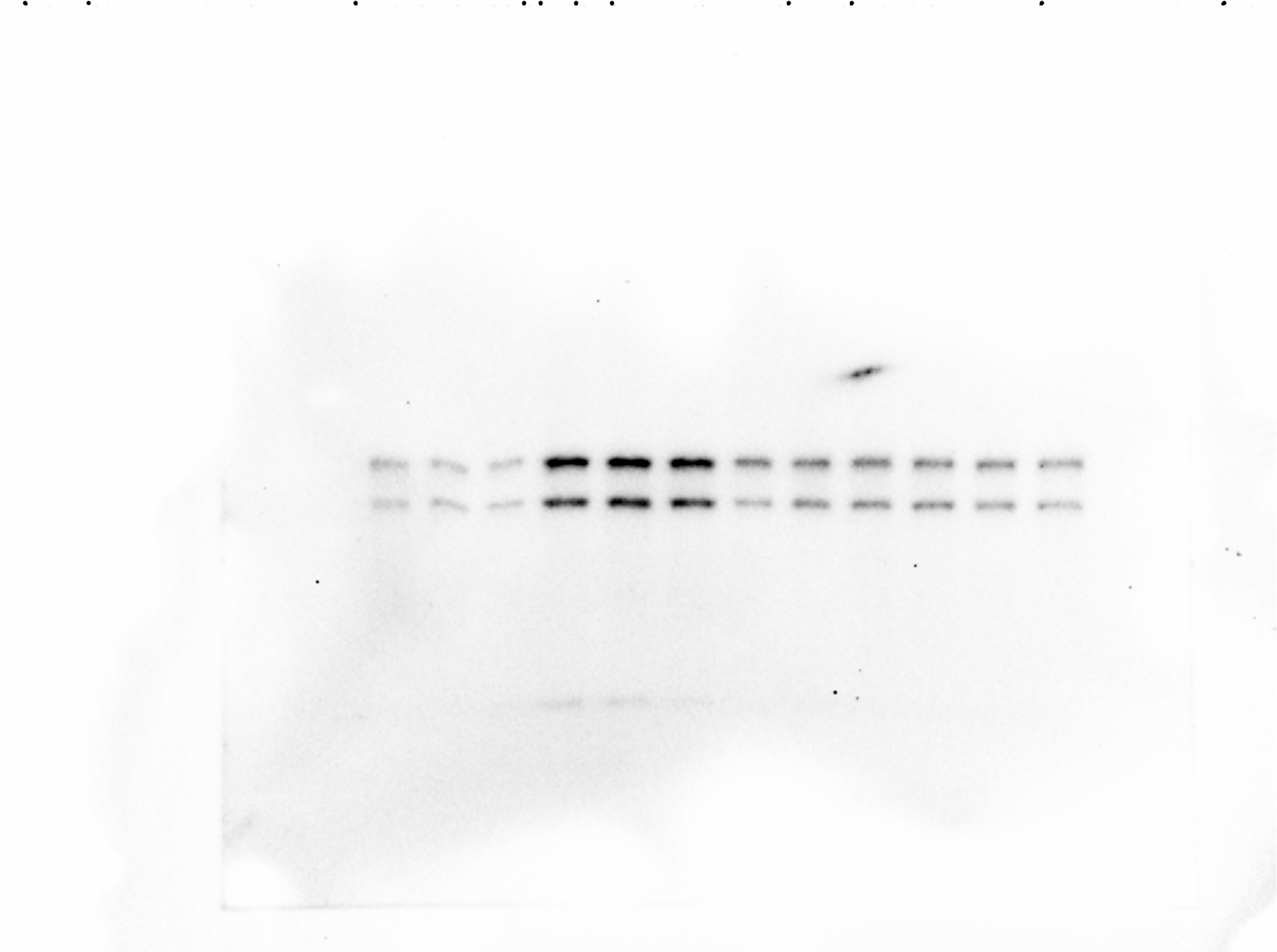

Supplement: Figure 3—source data 2. [file elife-68843-fig3-data2.zip › Figure 3D-Original WB images/Fig.3D p-JNK.tif]

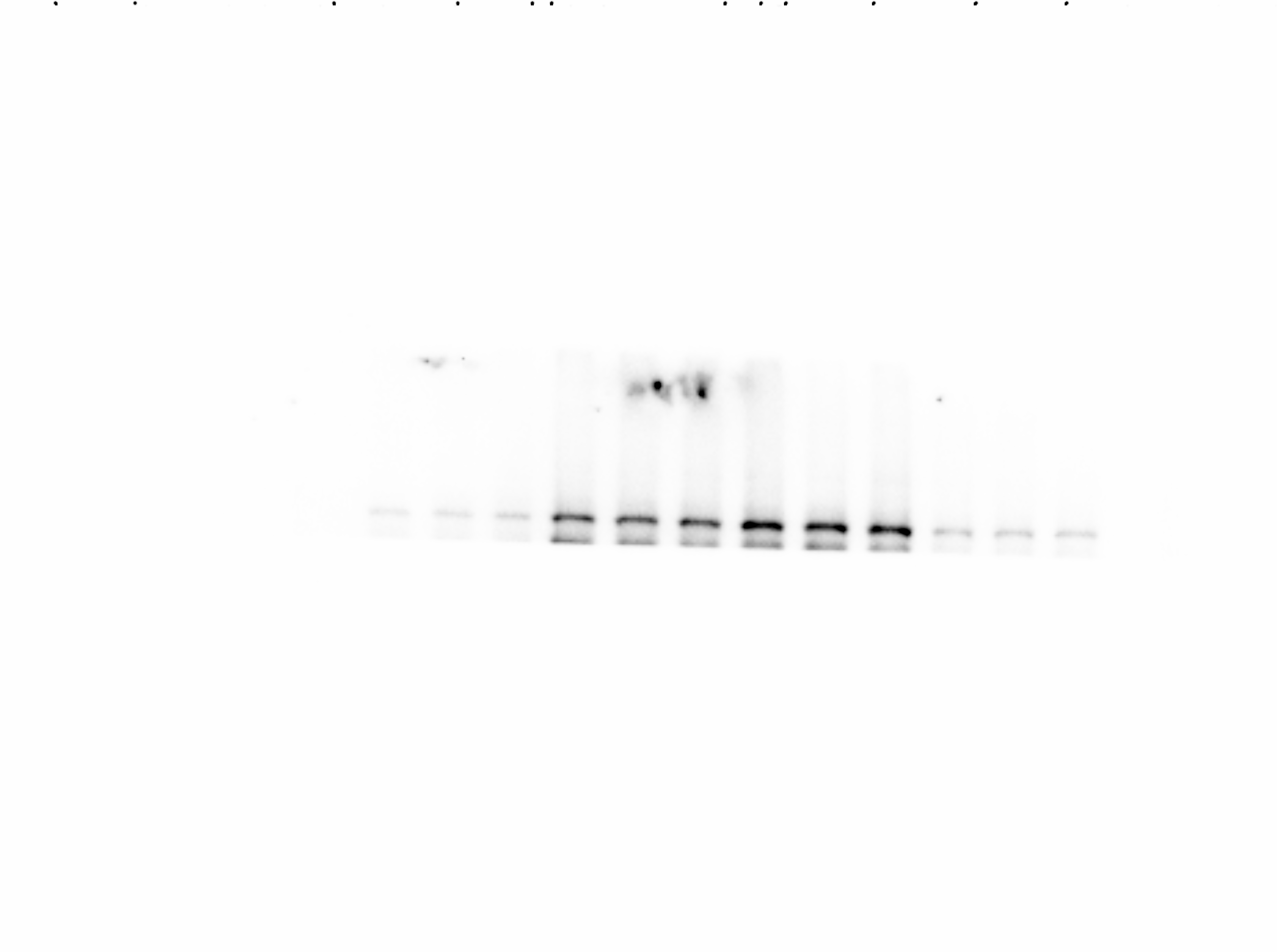

Supplement: Figure 3—source data 2. [file elife-68843-fig3-data2.zip › Figure 3D-Original WB images/Fig.3D p-STAT3.tif]

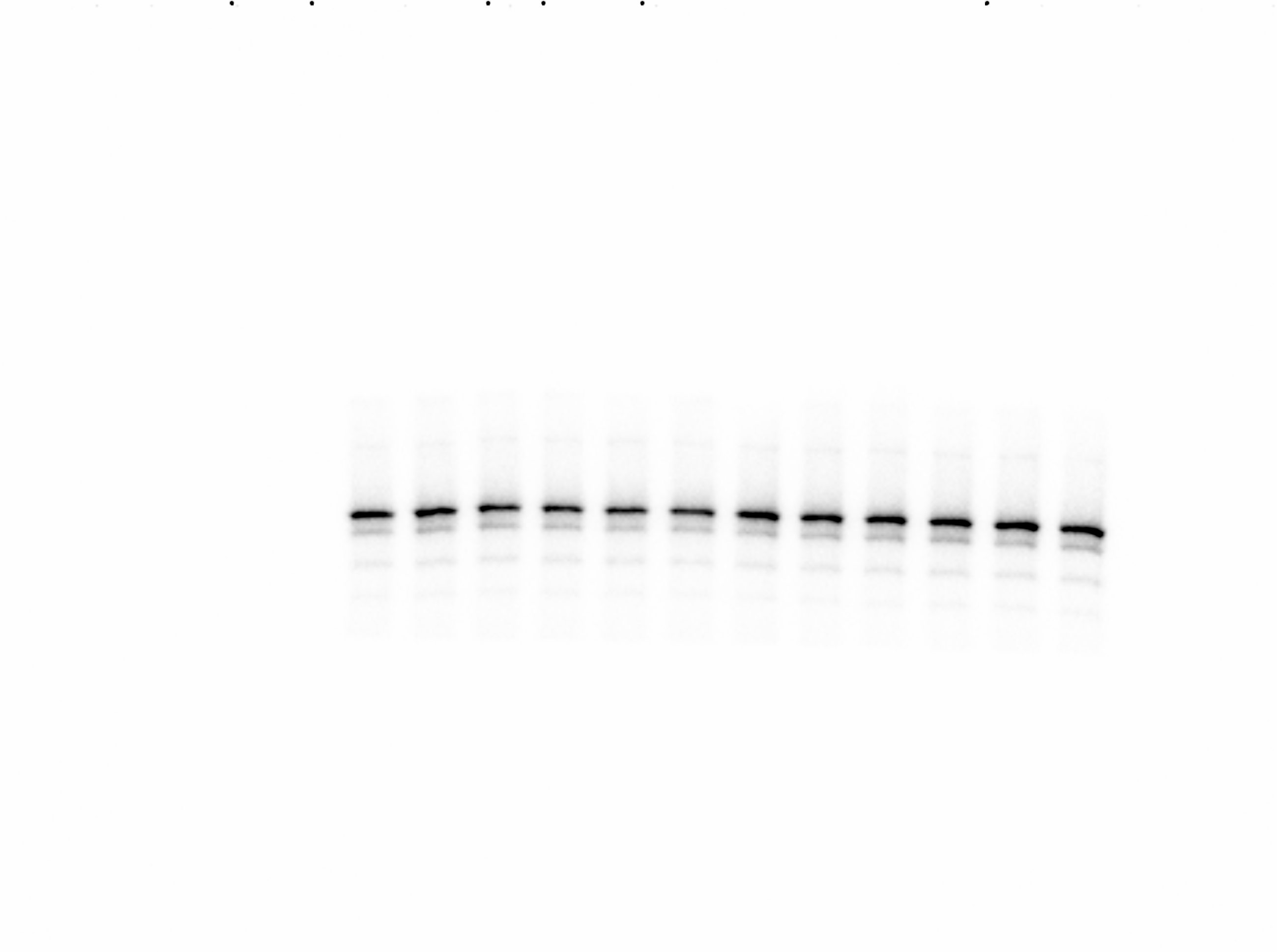

Supplement: Figure 3—source data 2. [file elife-68843-fig3-data2.zip › Figure 3D-Original WB images/Fig.3D STAT3.tif]

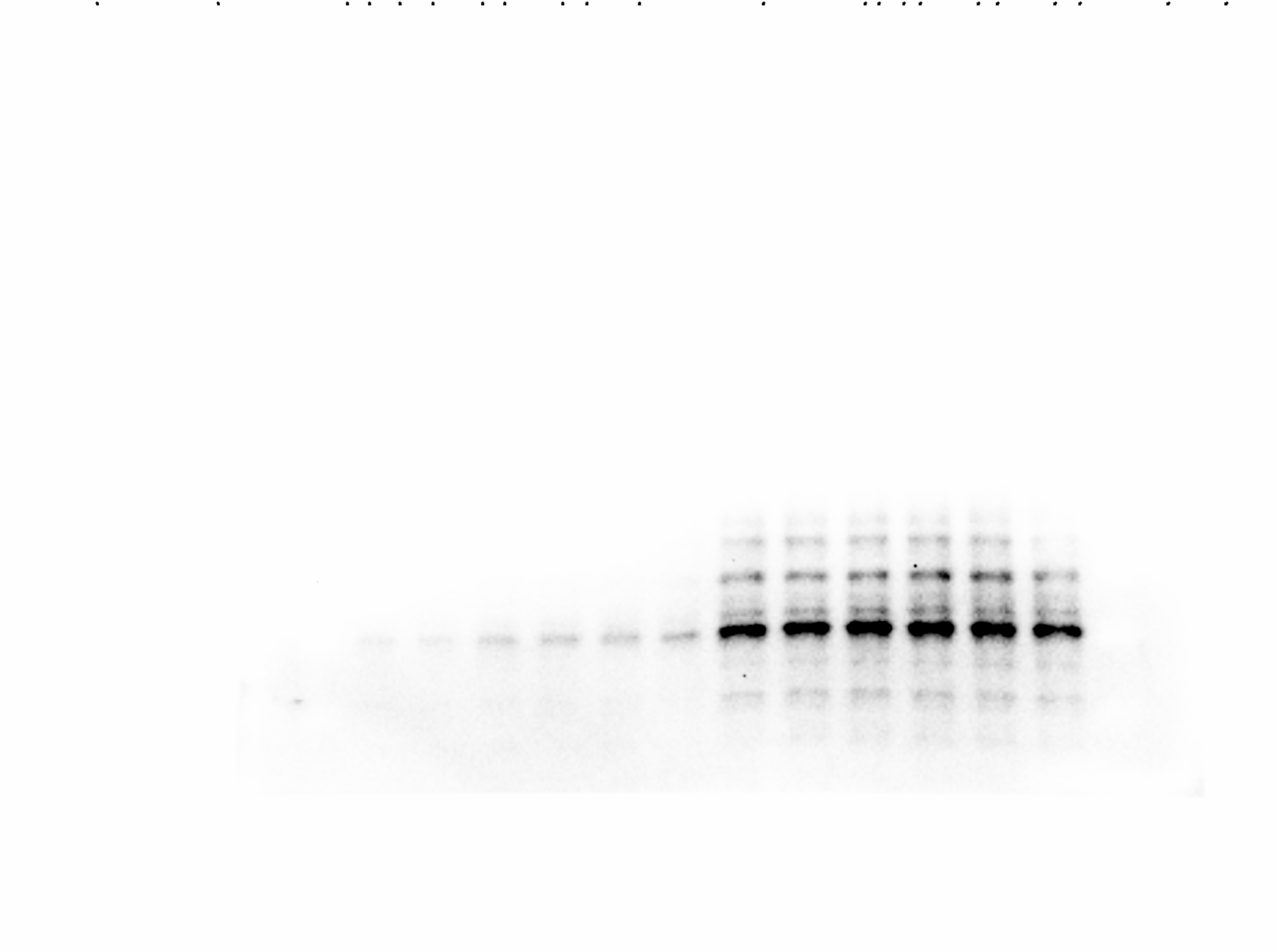

Supplement: Figure 3—source data 2. [file elife-68843-fig3-data2.zip › Figure 3I-Original WB images/Fig.3I CyclinD1.tif]

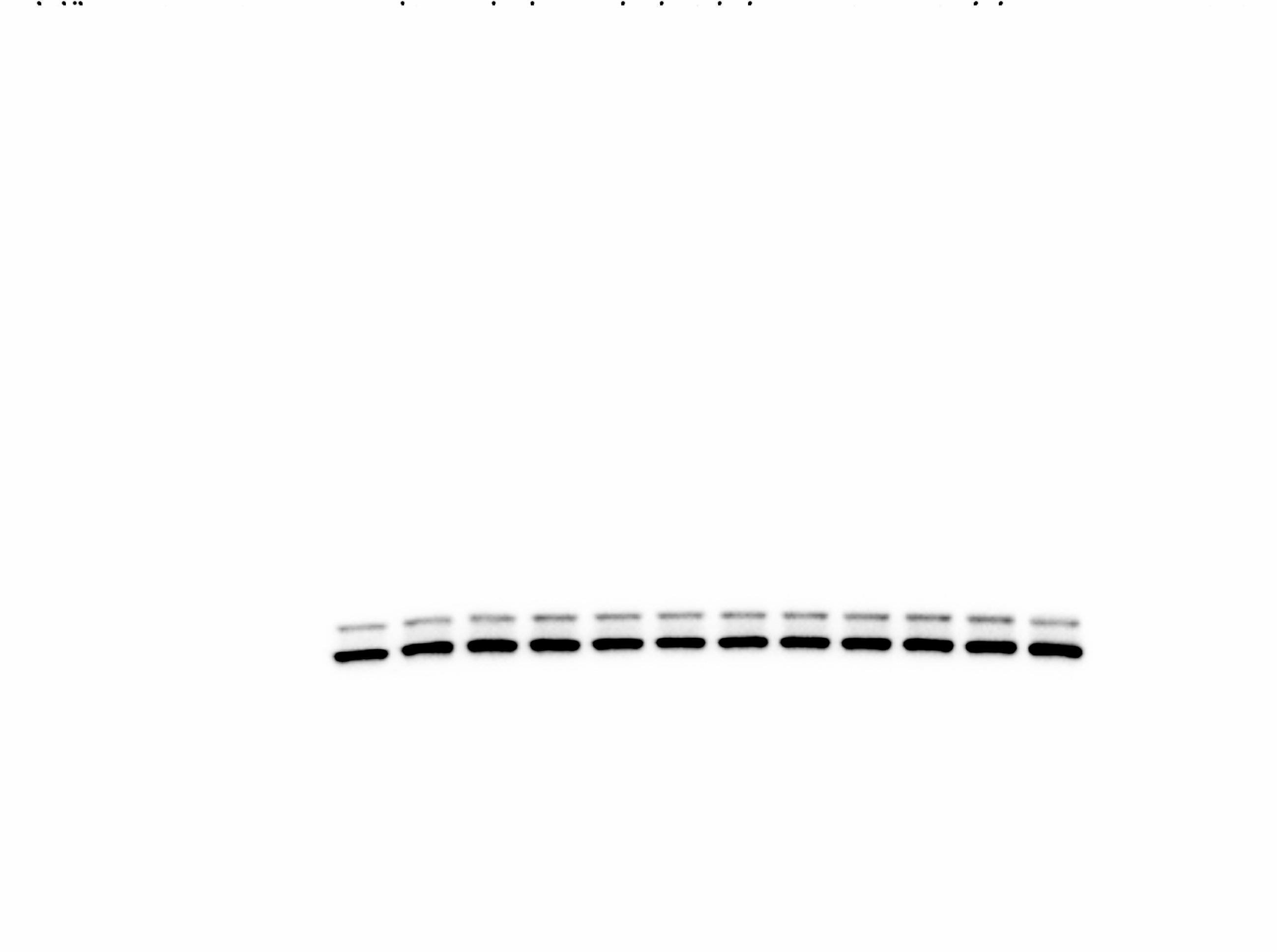

Supplement: Figure 3—source data 2. [file elife-68843-fig3-data2.zip › Figure 3I-Original WB images/Fig.3I ERK.tif]

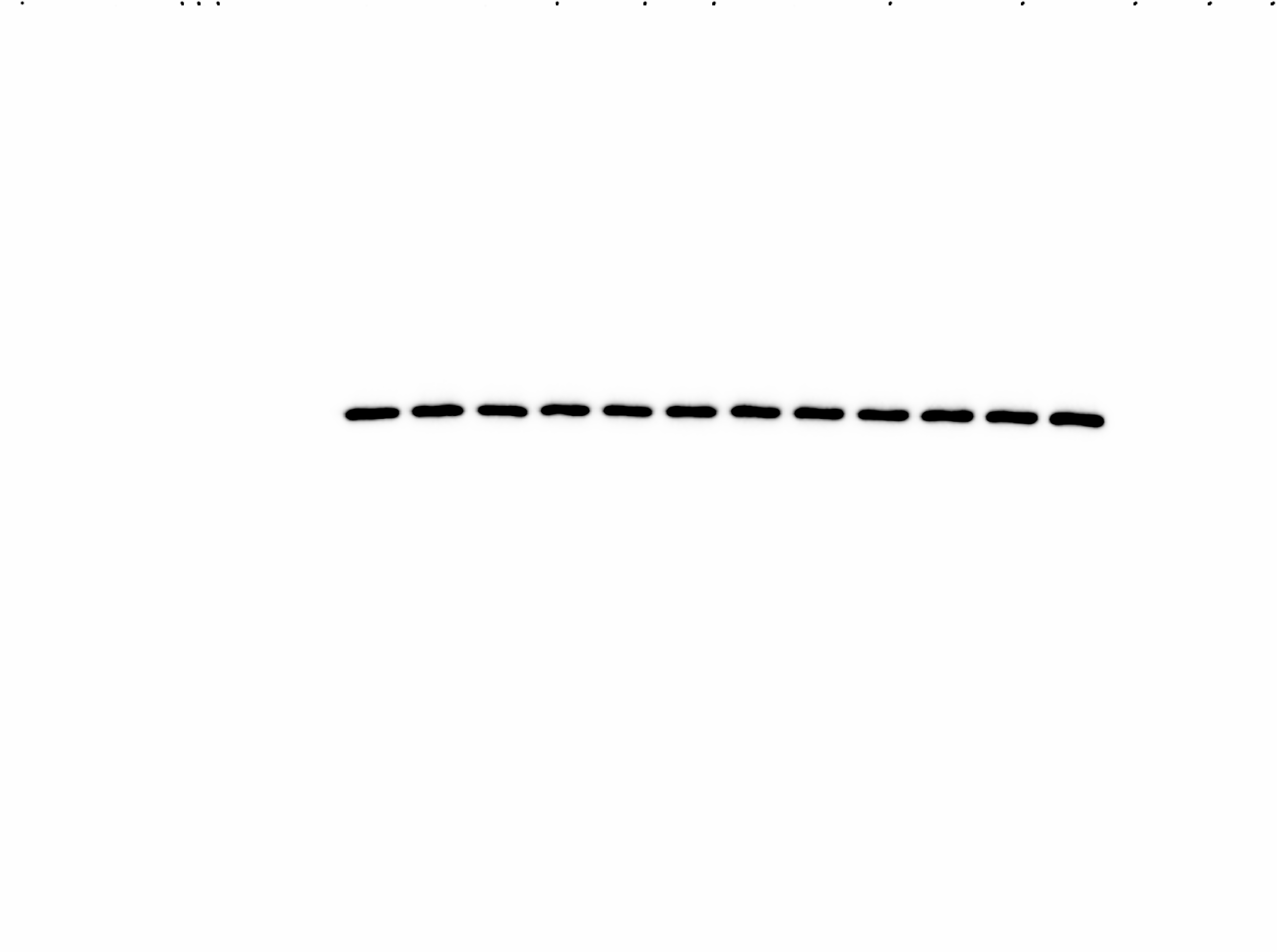

Supplement: Figure 3—source data 2. [file elife-68843-fig3-data2.zip › Figure 3I-Original WB images/Fig.3I GAPDH.tif]

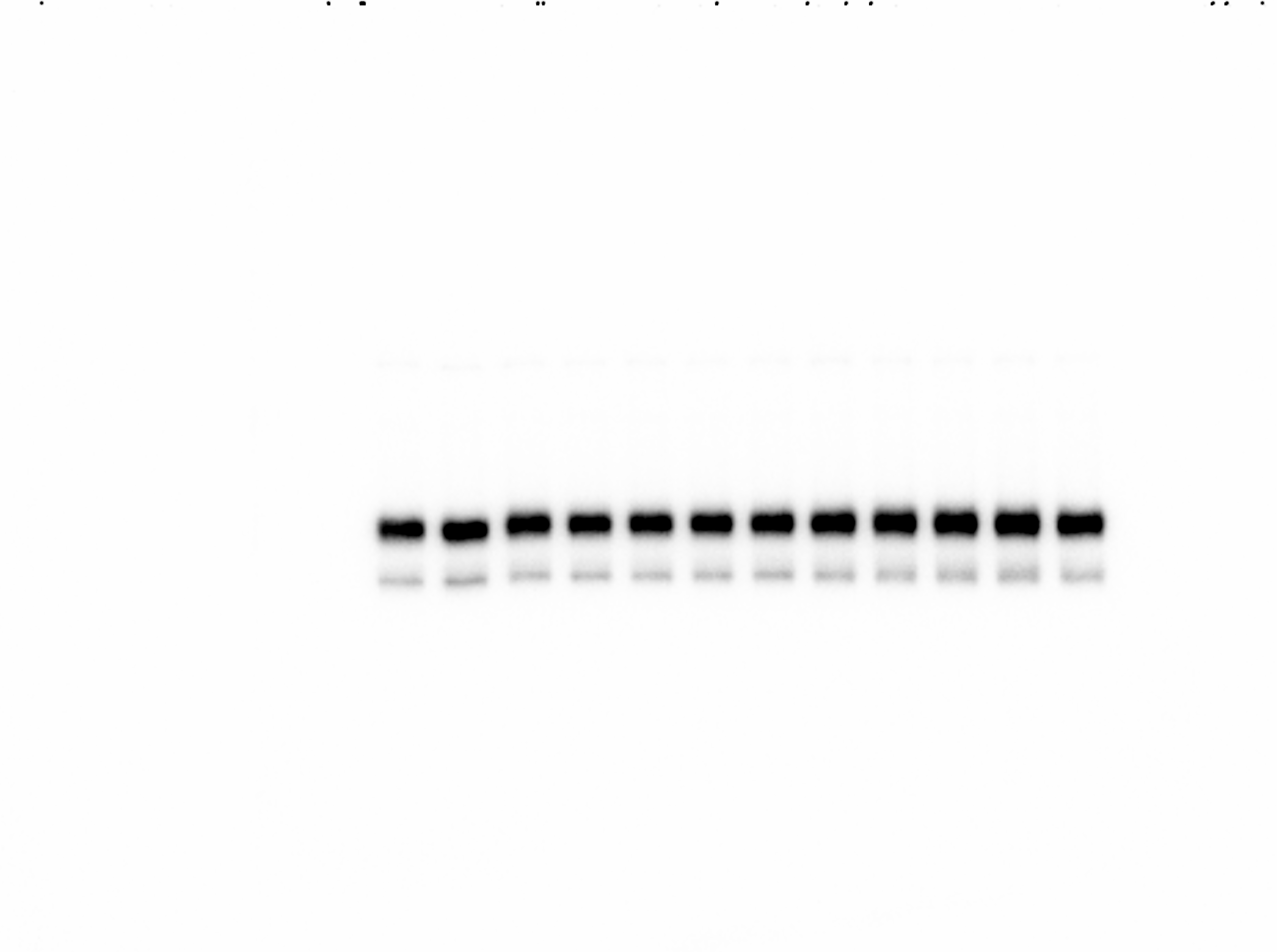

Supplement: Figure 3—source data 2. [file elife-68843-fig3-data2.zip › Figure 3I-Original WB images/Fig.3I JNK.tif]

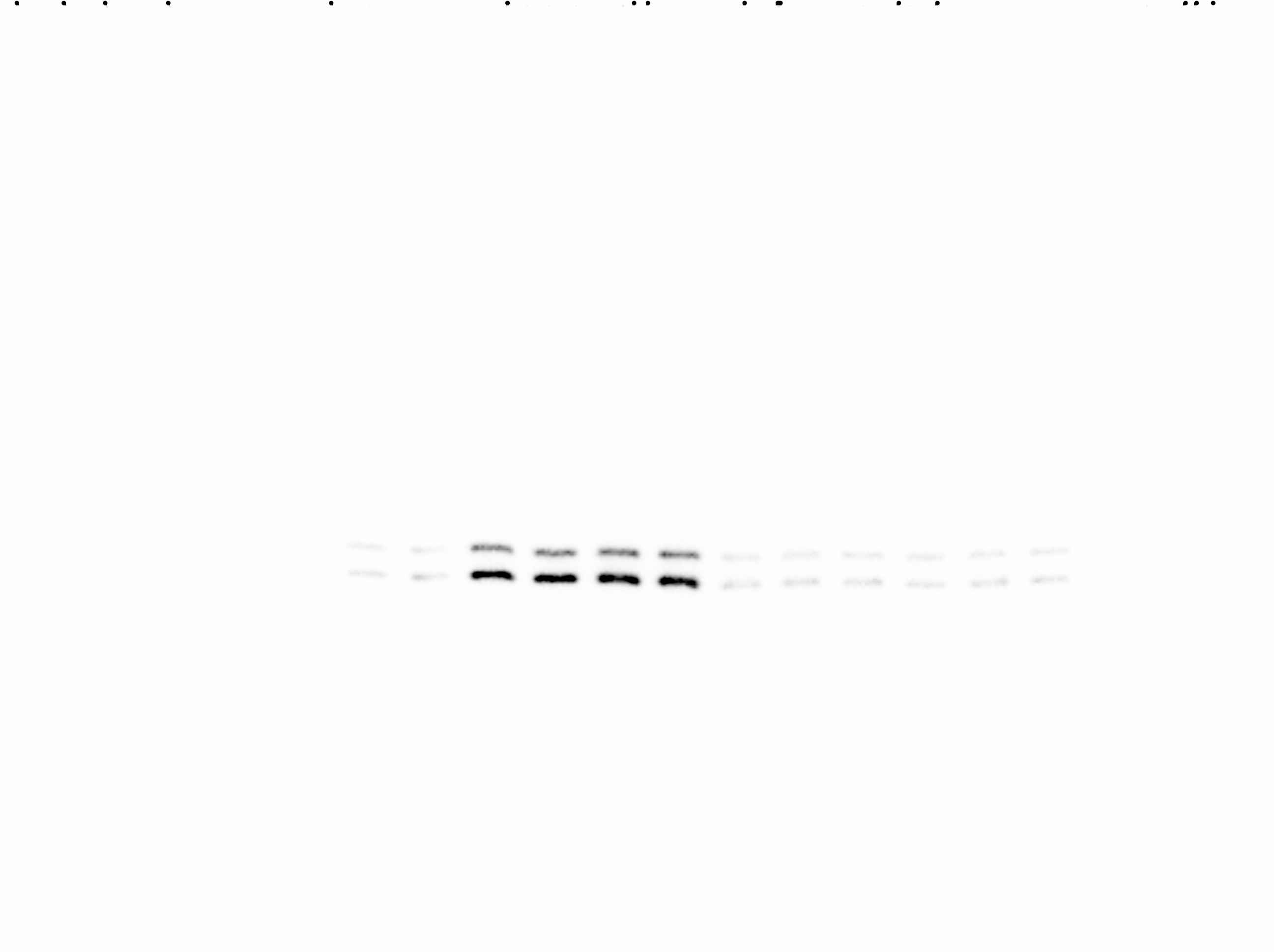

Supplement: Figure 3—source data 2. [file elife-68843-fig3-data2.zip › Figure 3I-Original WB images/Fig.3I p-ERK.tif]

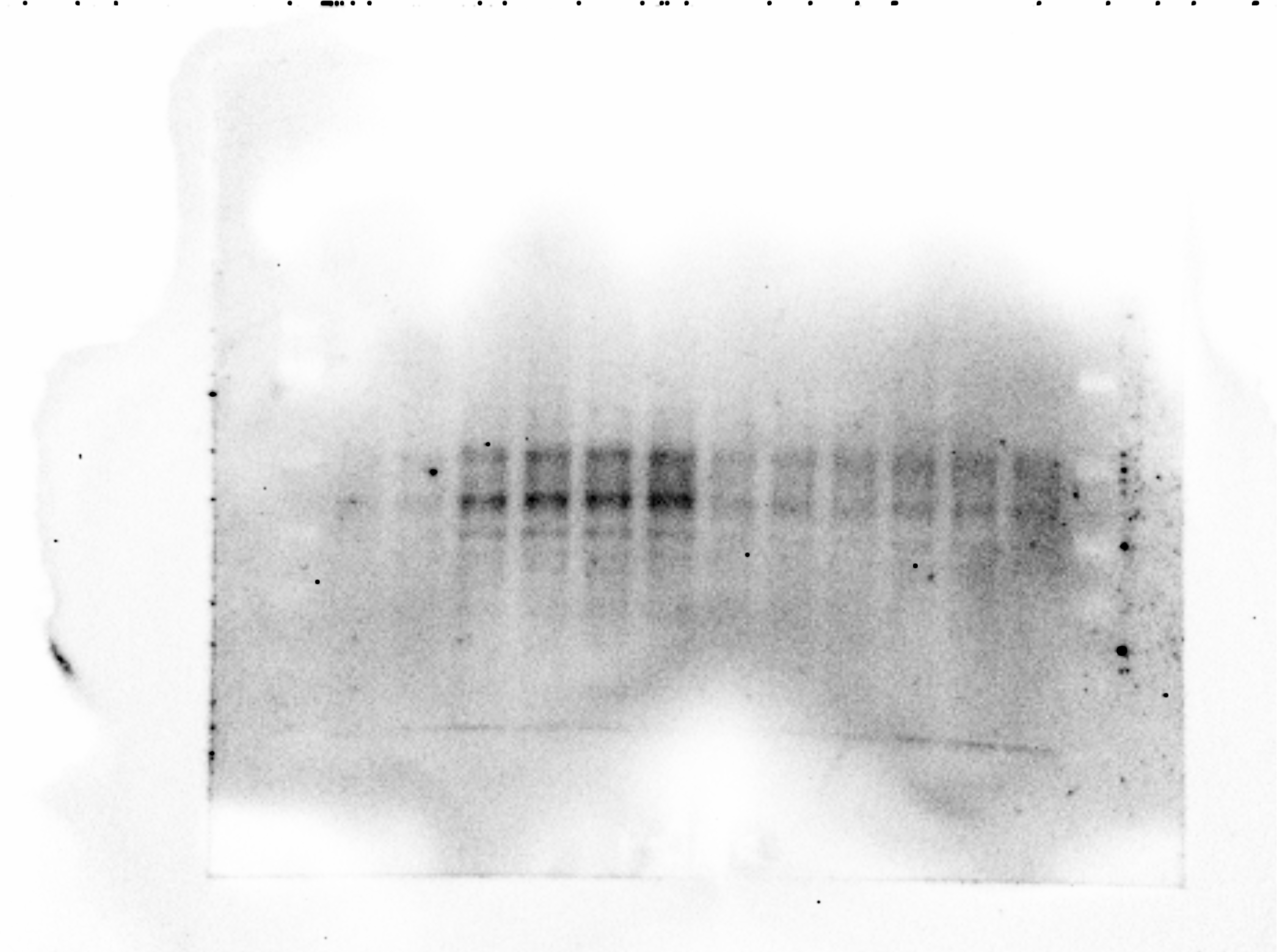

Supplement: Figure 3—source data 2. [file elife-68843-fig3-data2.zip › Figure 3I-Original WB images/Fig.3I p-JNK.tif]

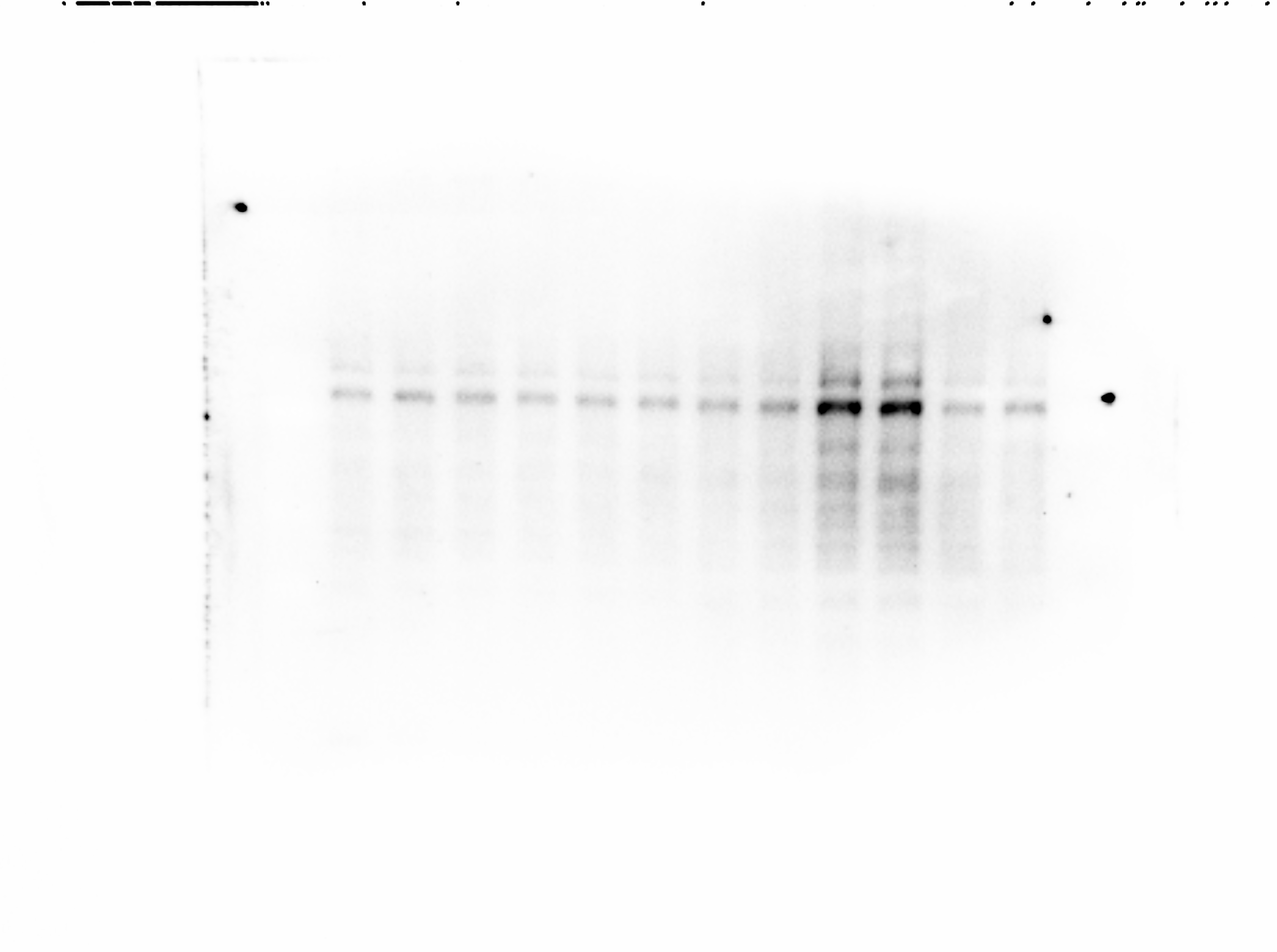

Supplement: Figure 3—source data 2. [file elife-68843-fig3-data2.zip › Figure 3I-Original WB images/Fig.3I p-STAT3.tif]

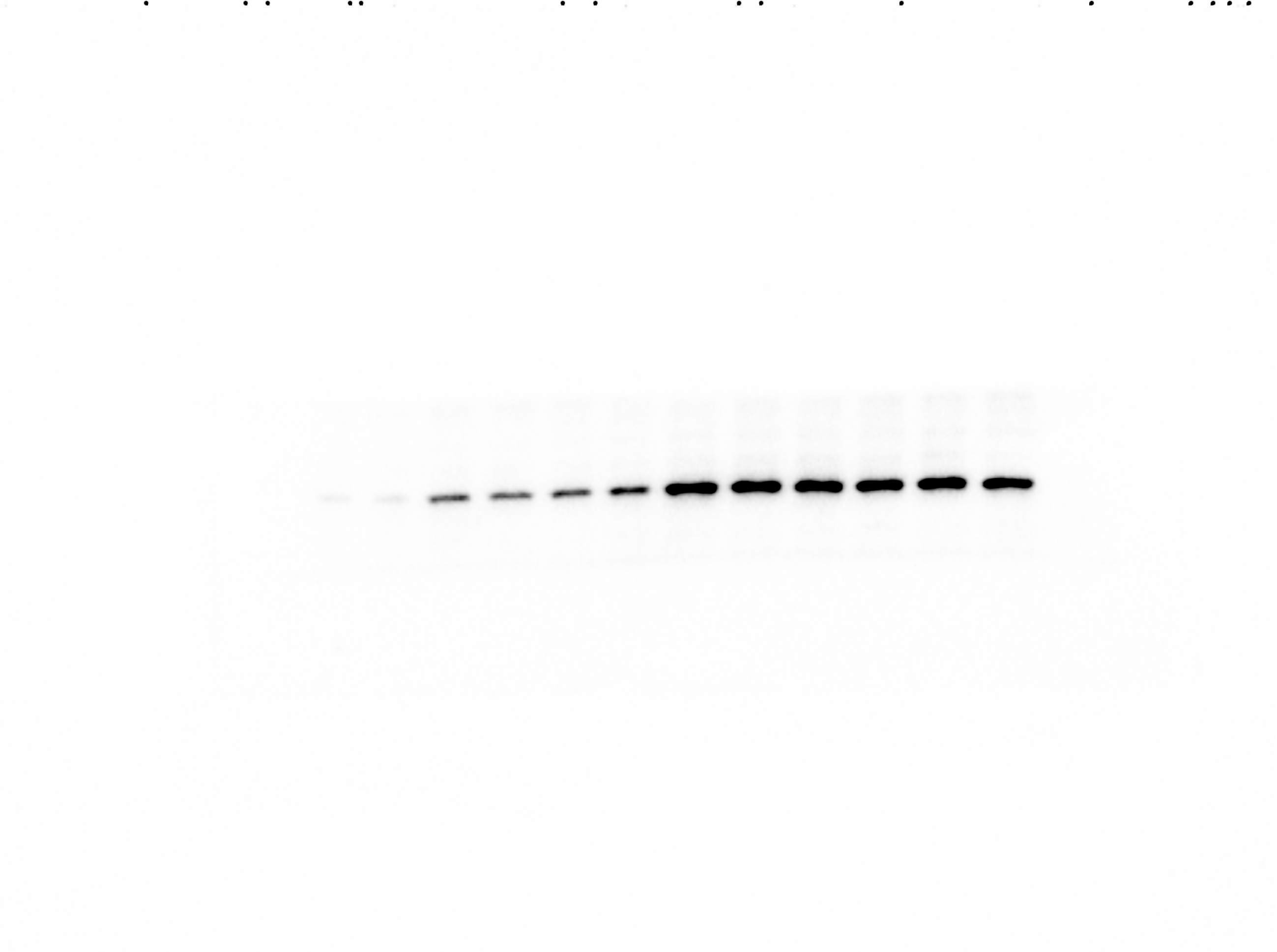

Supplement: Figure 3—source data 2. [file elife-68843-fig3-data2.zip › Figure 3I-Original WB images/Fig.3I PCNA.tif]

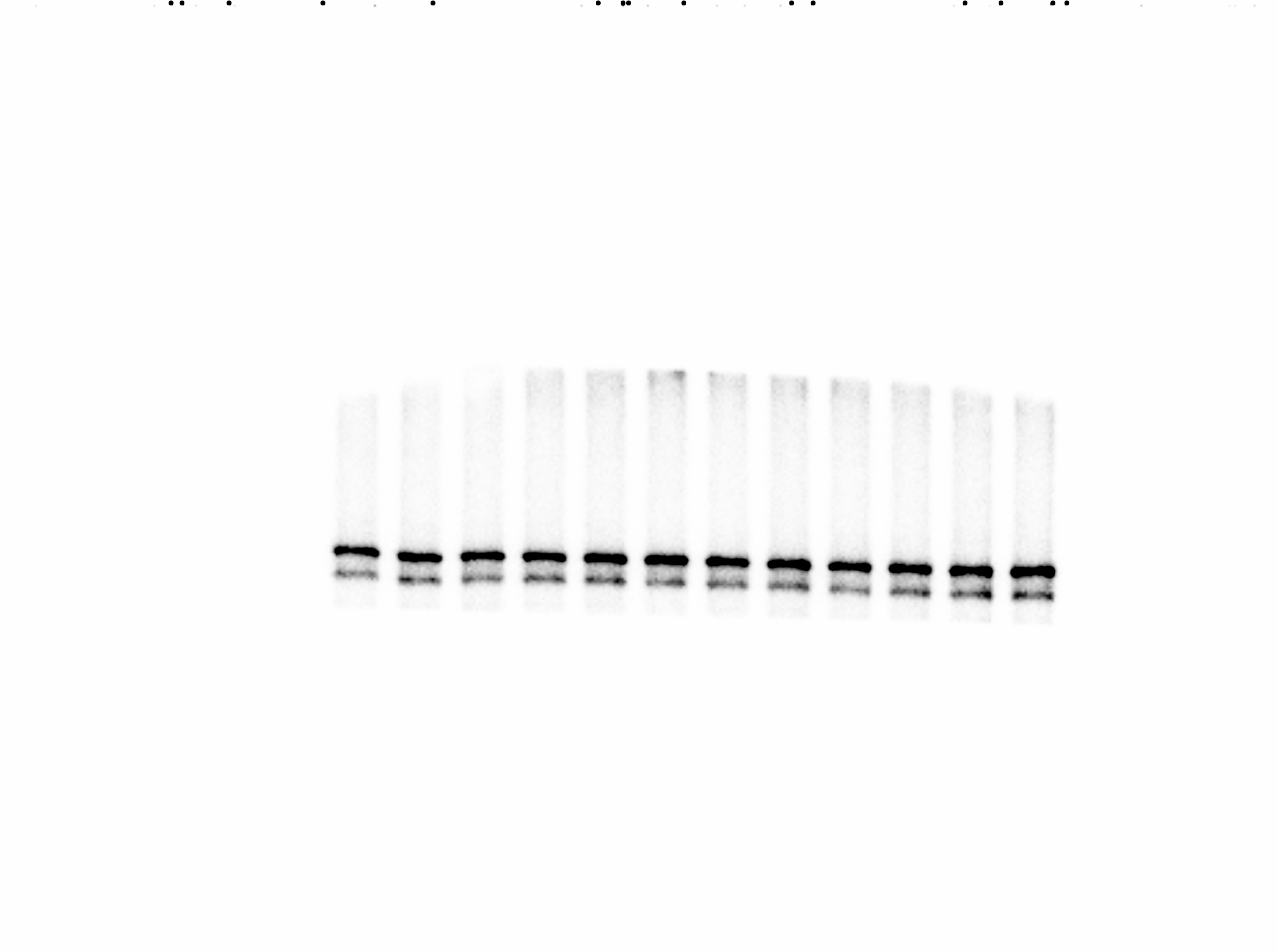

Supplement: Figure 3—source data 2. [file elife-68843-fig3-data2.zip › Figure 3I-Original WB images/Fig.3I STAT3.tif]

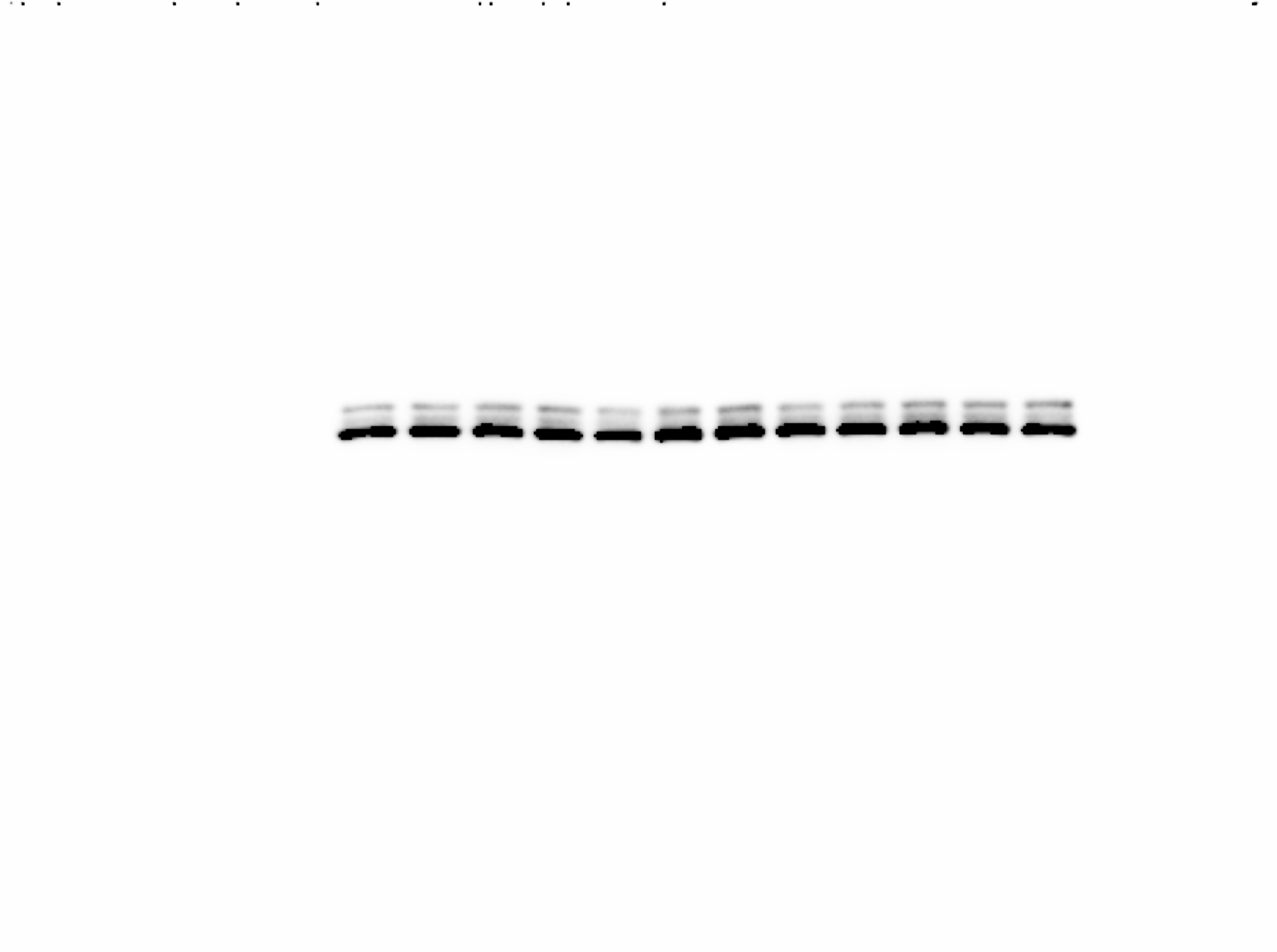

Supplement: Figure 3—source data 2. [file elife-68843-fig3-data2.zip › Figure 3N-Original WB images/Fig.3N ERK.tif]

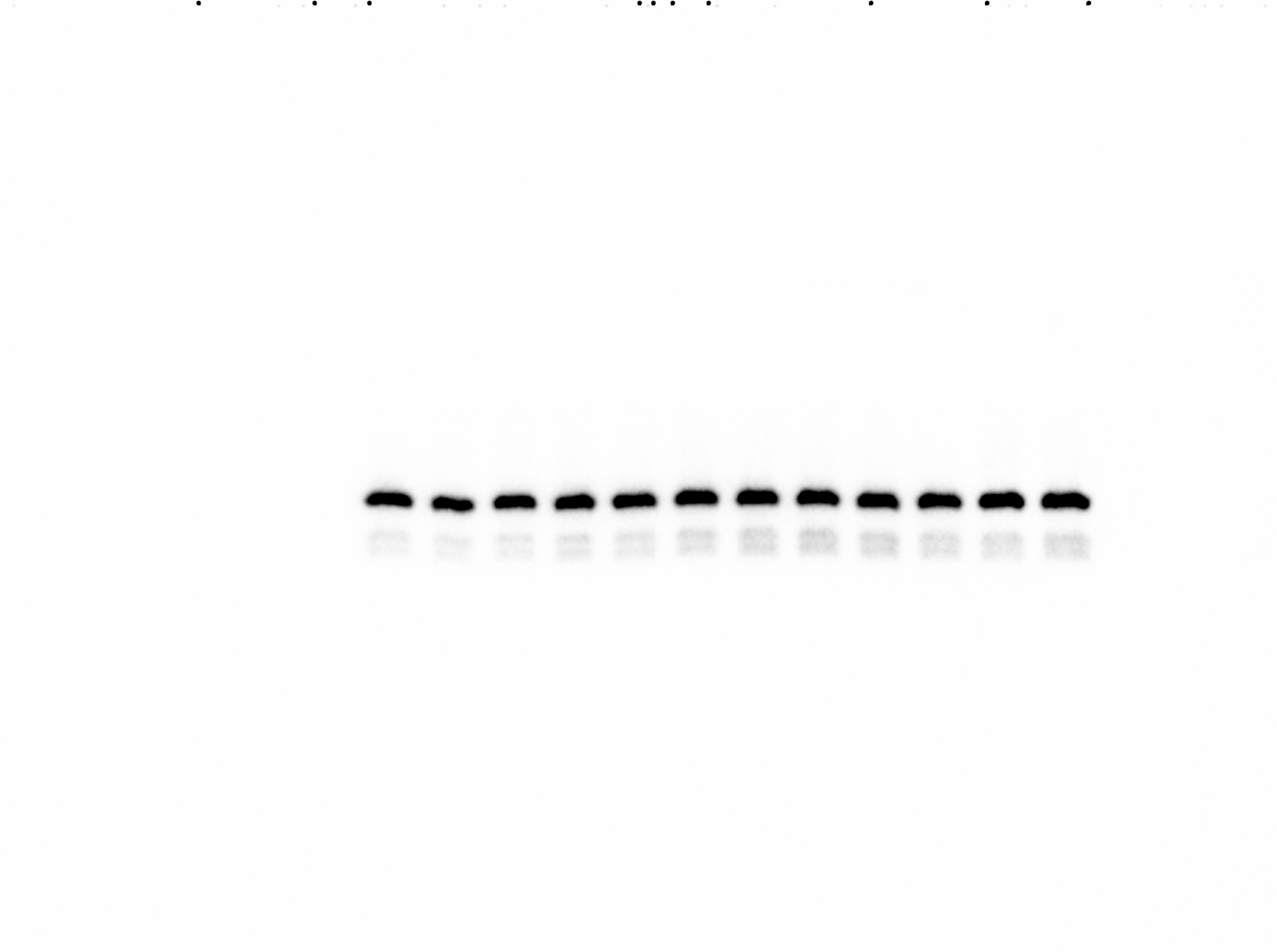

Supplement: Figure 3—source data 2. [file elife-68843-fig3-data2.zip › Figure 3N-Original WB images/Fig.3N JNK.tif]

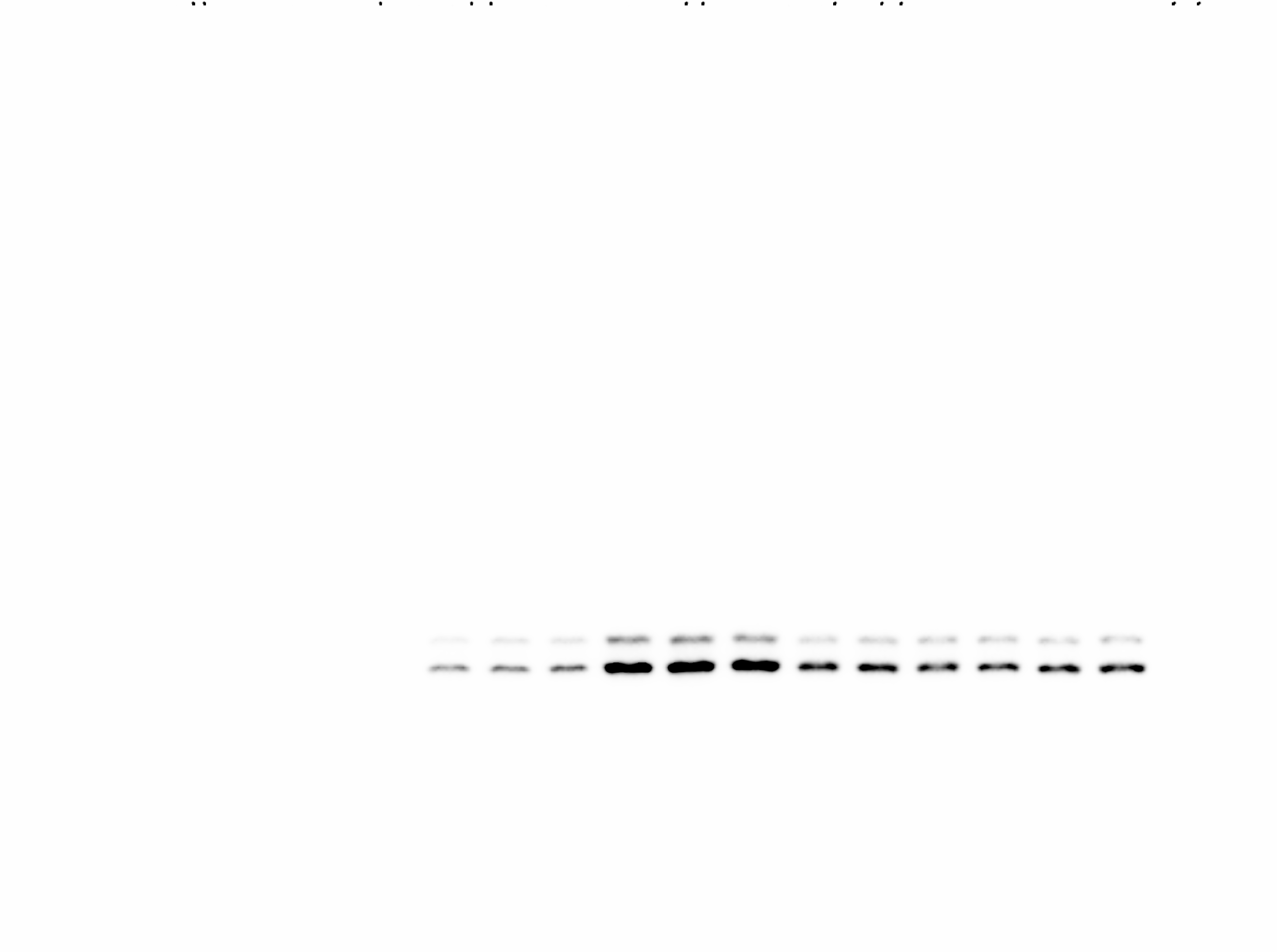

Supplement: Figure 3—source data 2. [file elife-68843-fig3-data2.zip › Figure 3N-Original WB images/Fig.3N p-ERK.tif]

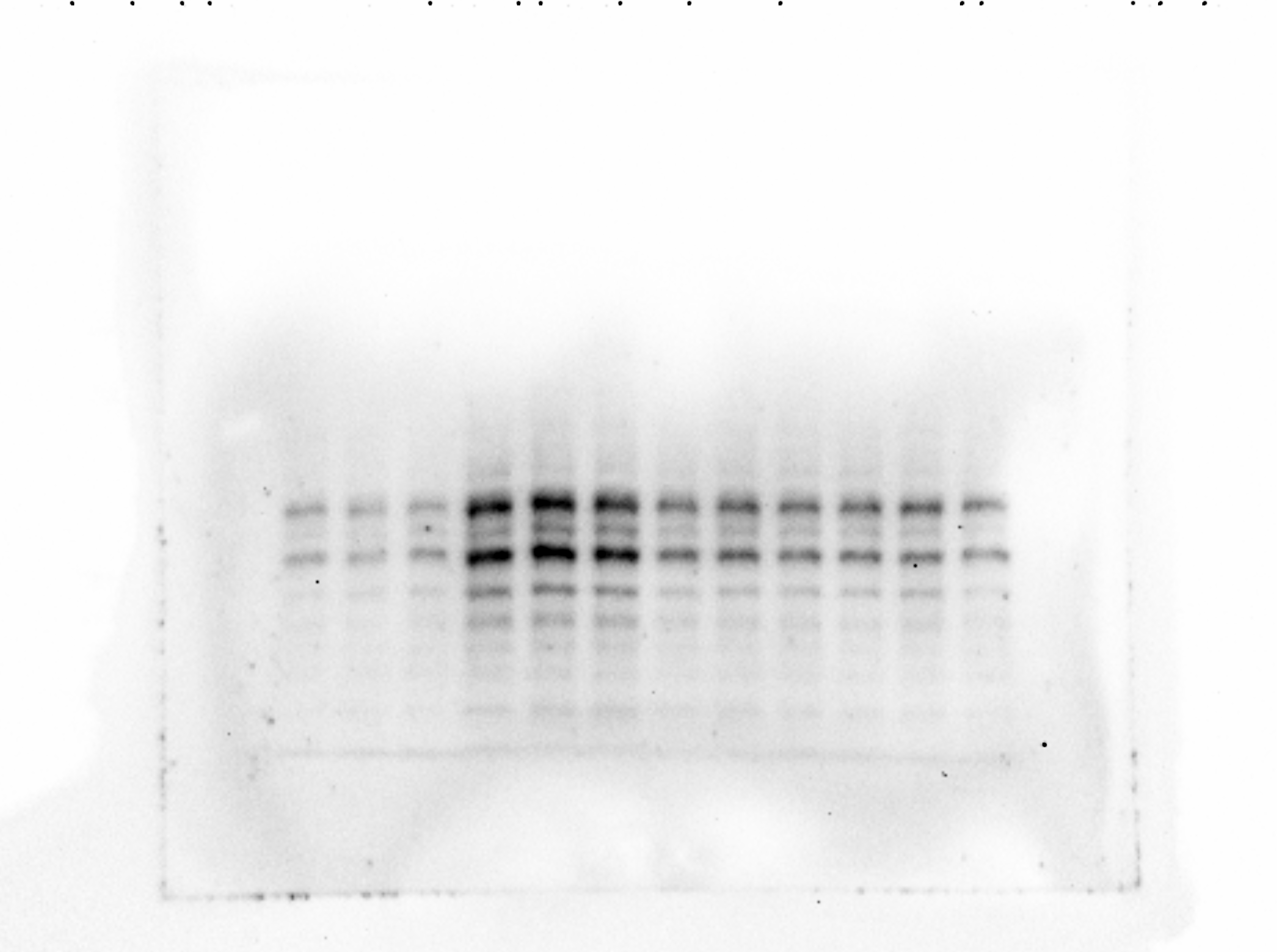

Supplement: Figure 3—source data 2. [file elife-68843-fig3-data2.zip › Figure 3N-Original WB images/Fig.3N p-JNK.tif]

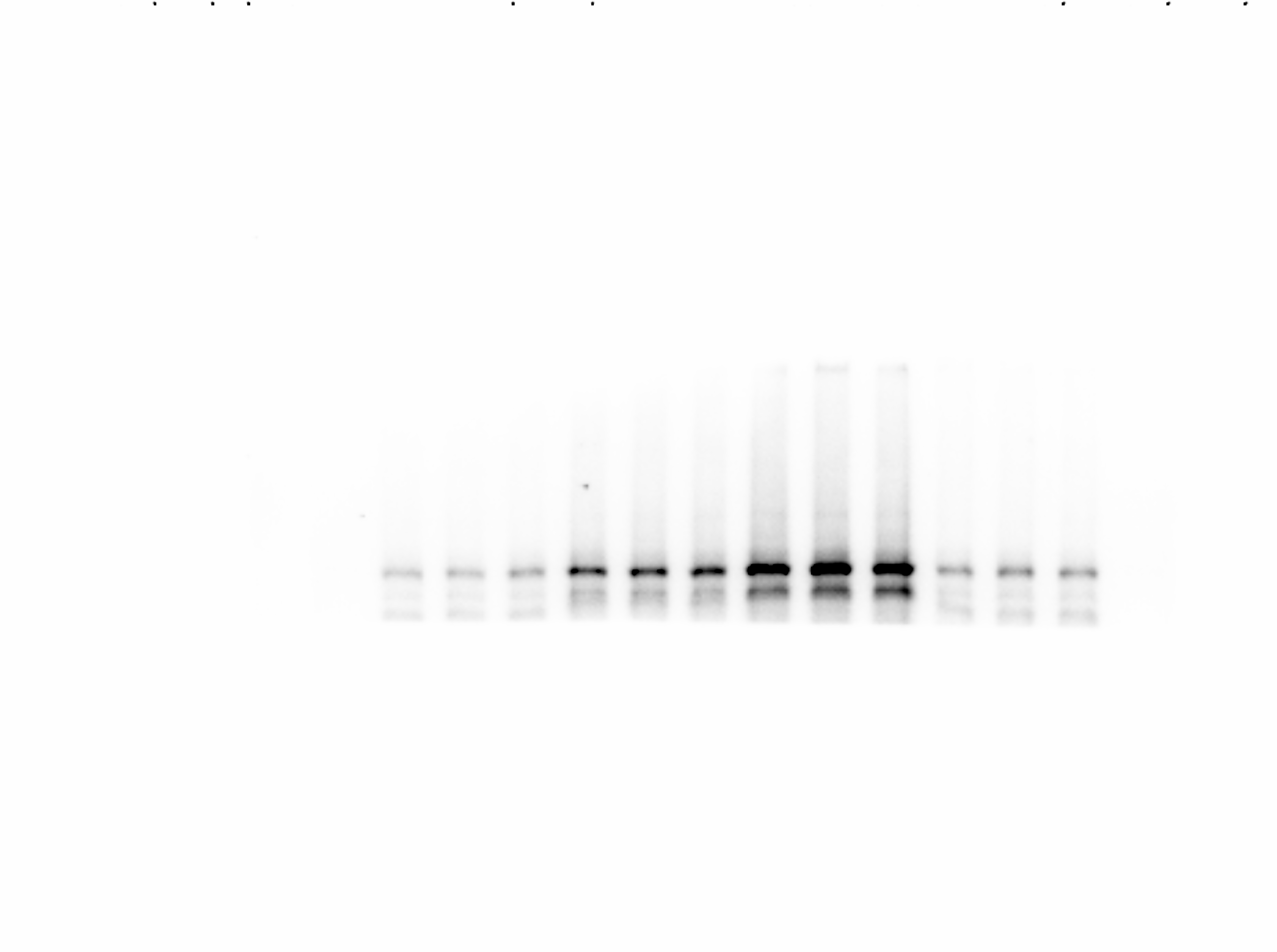

Supplement: Figure 3—source data 2. [file elife-68843-fig3-data2.zip › Figure 3N-Original WB images/Fig.3N p-STAT3.tif]

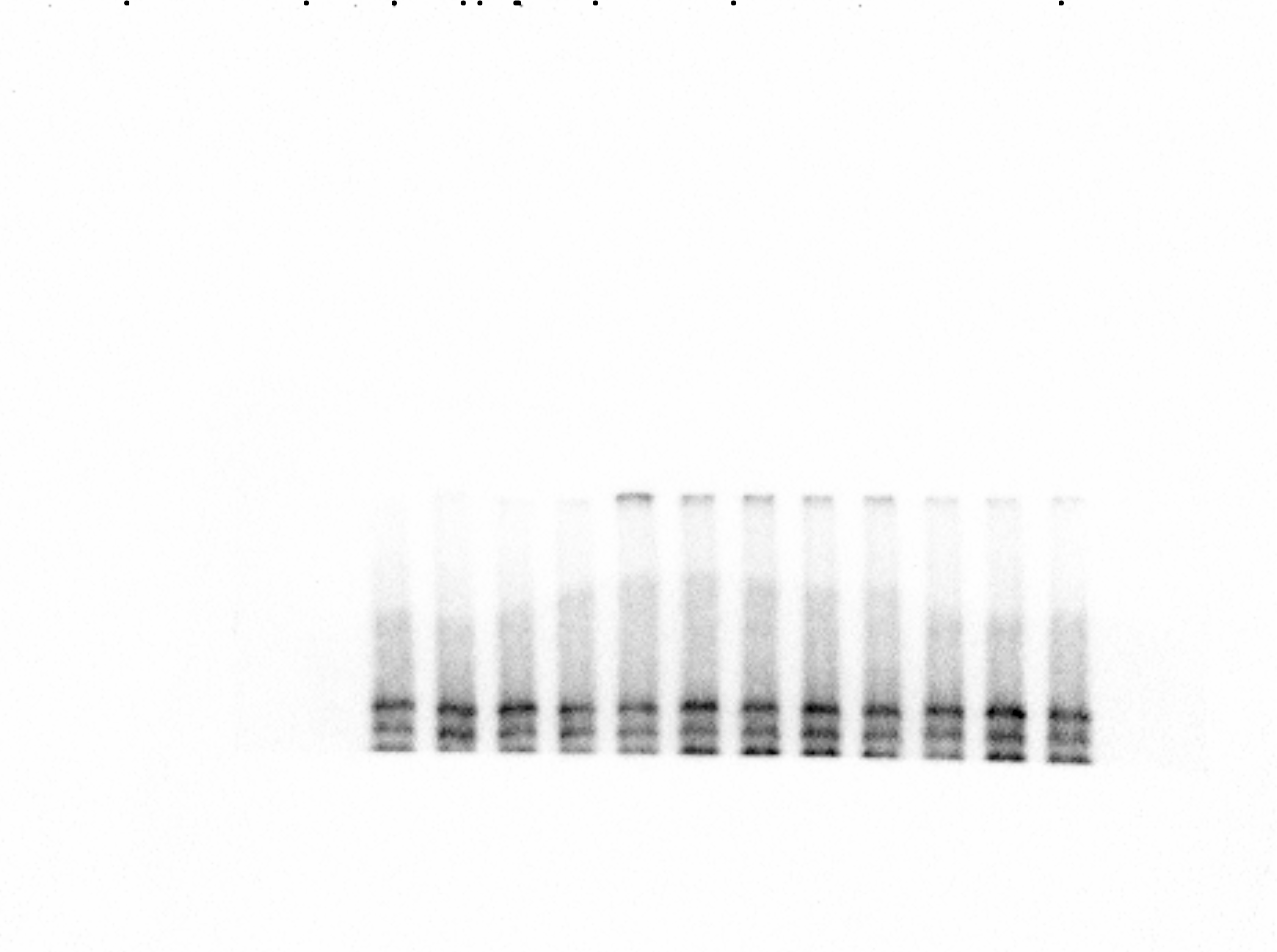

Supplement: Figure 3—source data 2. [file elife-68843-fig3-data2.zip › Figure 3N-Original WB images/Fig.3N STAT3.tif]

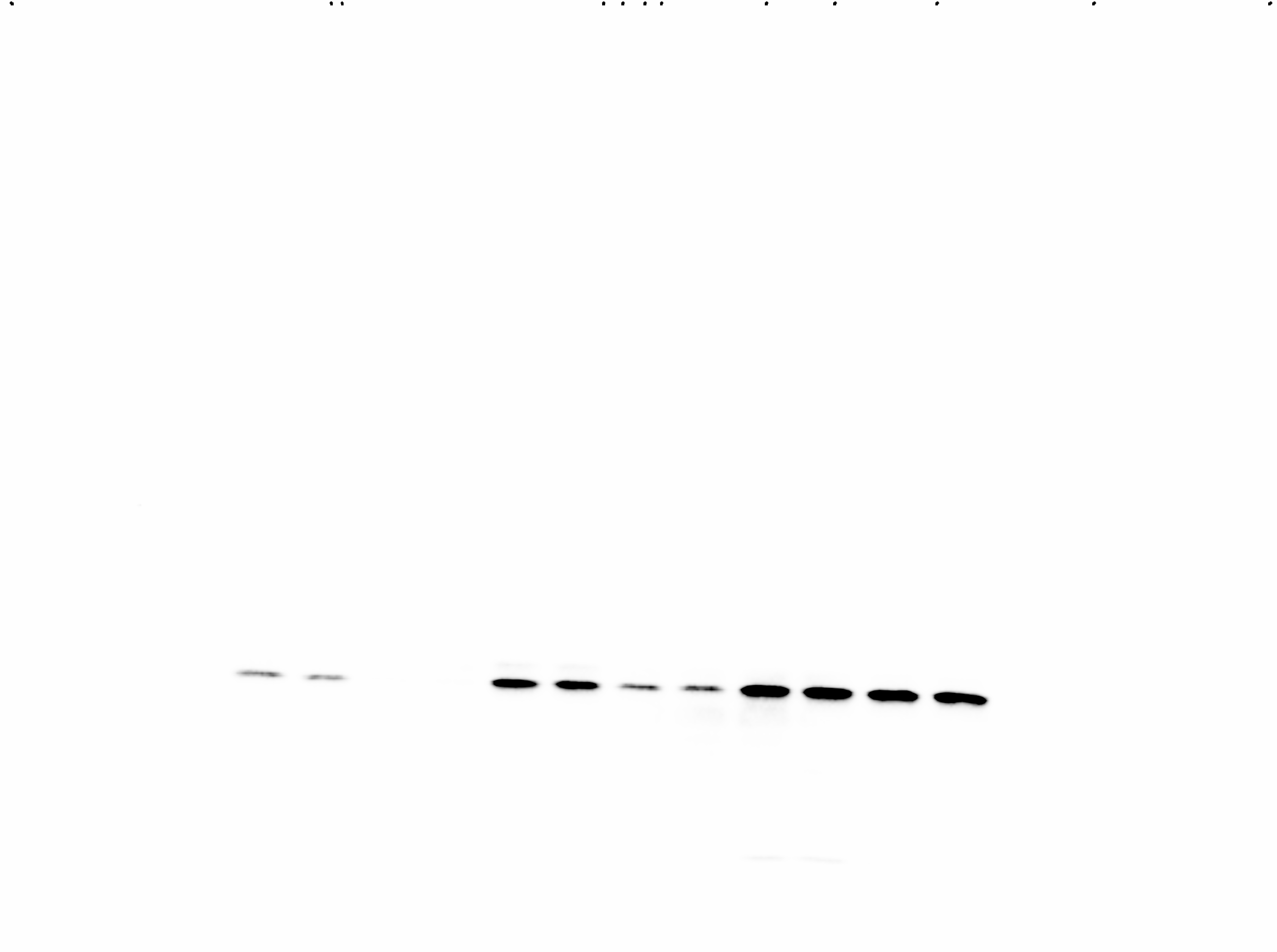

Supplement: Figure 4—source data 2. [file elife-68843-fig4-data2.zip › Figure 4B-Original WB images/Fig. 4B Cyclin D1.tif]

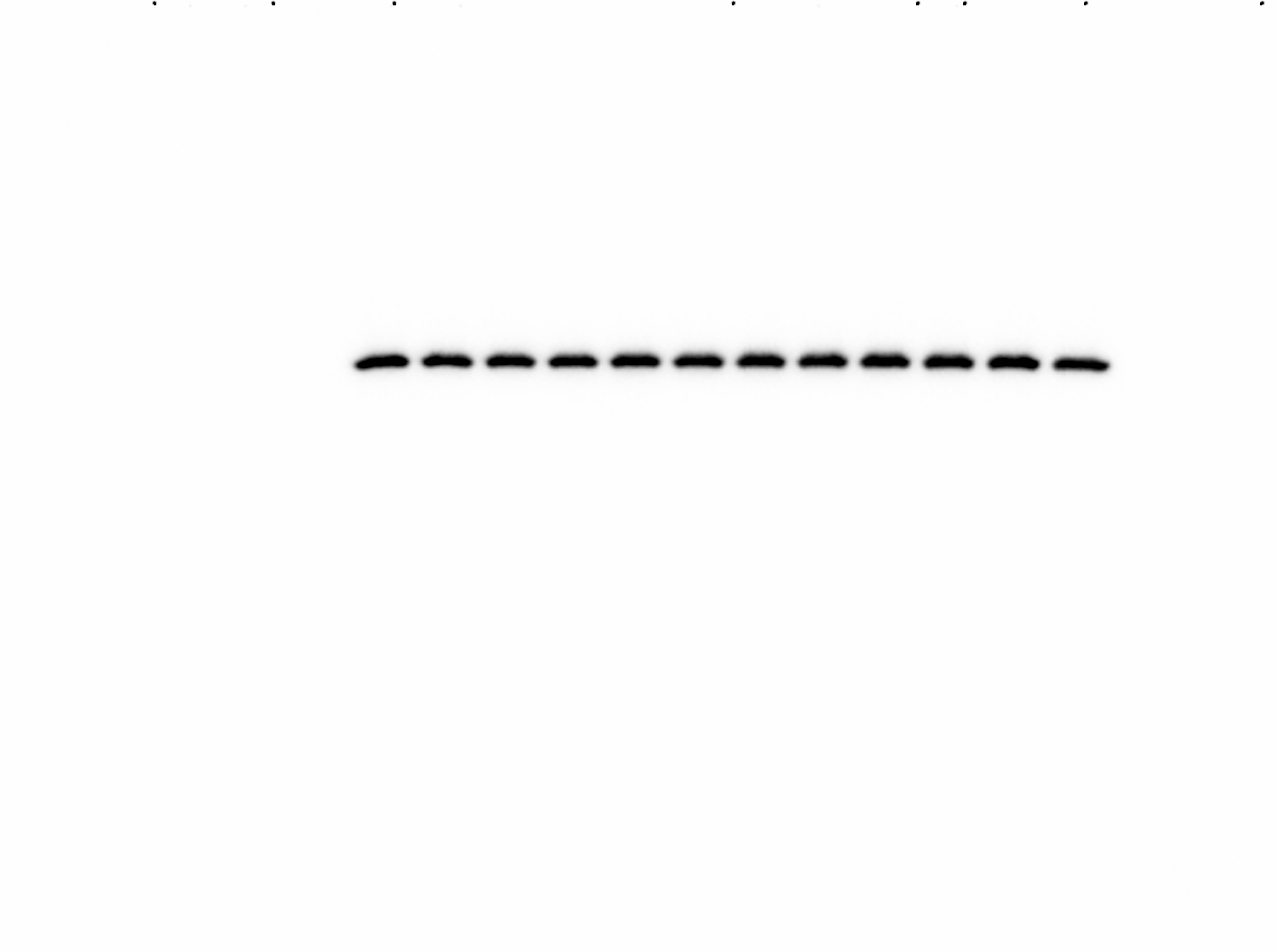

Supplement: Figure 4—source data 2. [file elife-68843-fig4-data2.zip › Figure 4B-Original WB images/Fig. 4B GAPDH.tif]

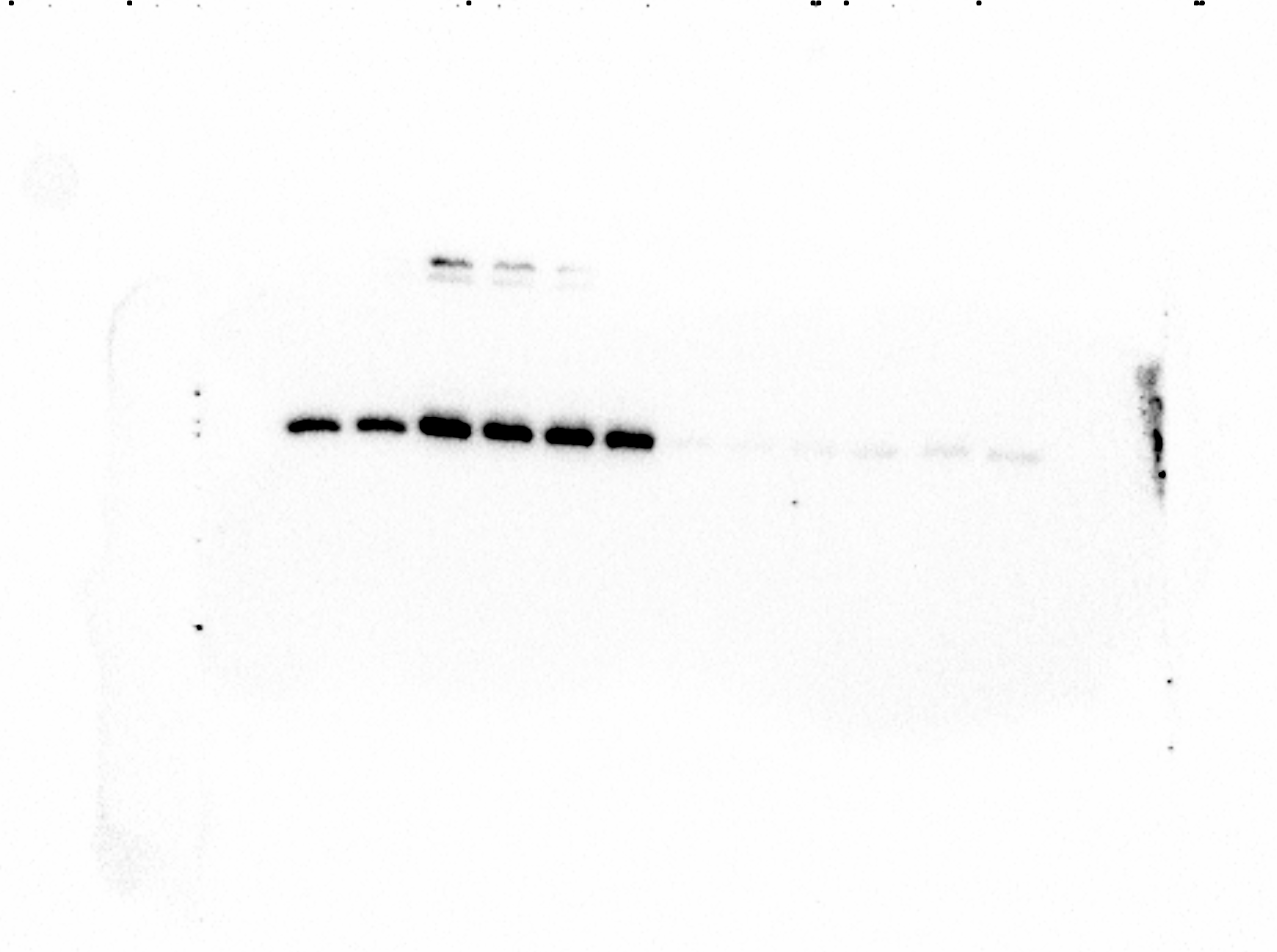

Supplement: Figure 4—source data 2. [file elife-68843-fig4-data2.zip › Figure 4B-Original WB images/Fig. 4B IL11.tif]

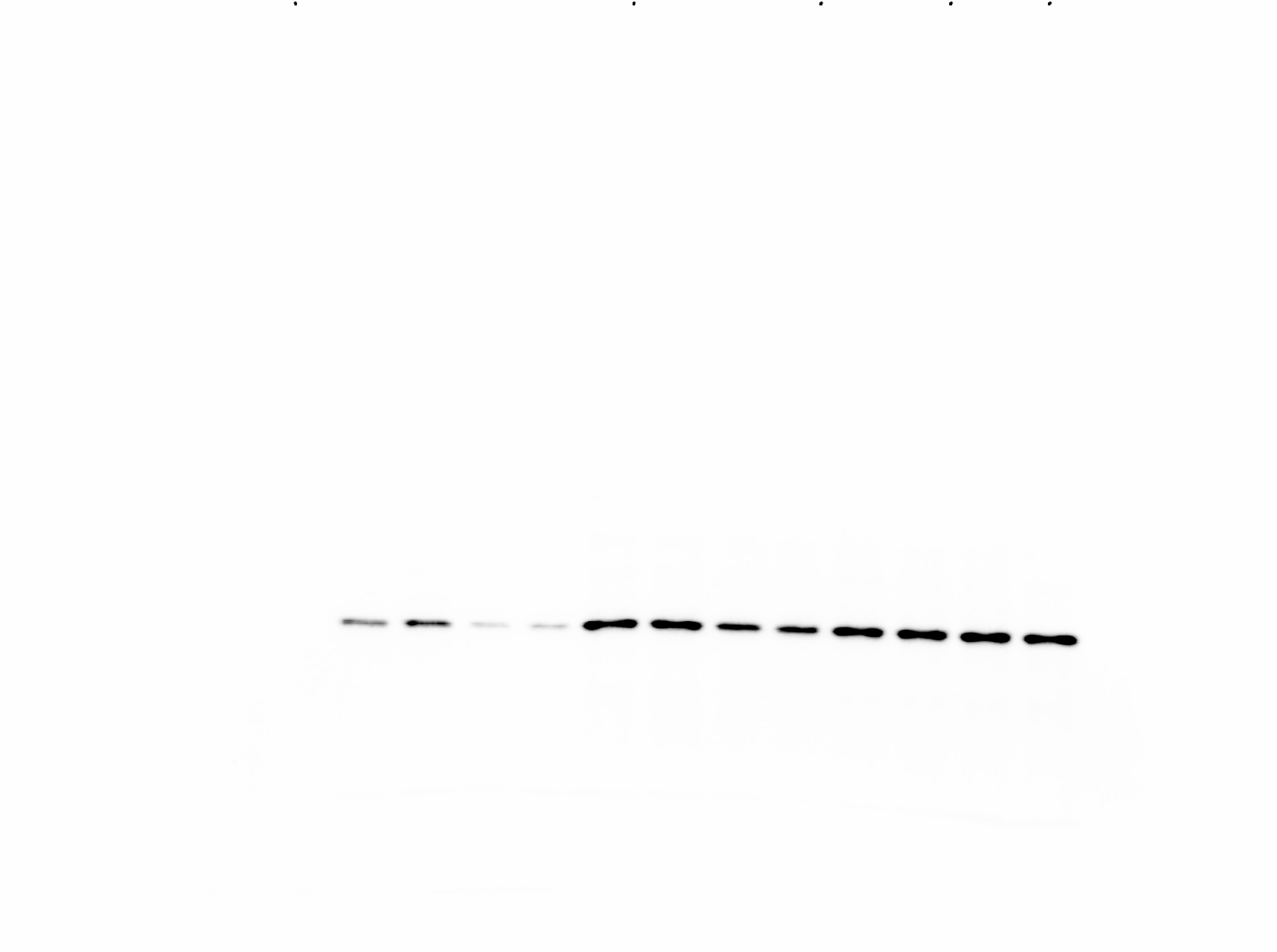

Supplement: Figure 4—source data 2. [file elife-68843-fig4-data2.zip › Figure 4B-Original WB images/Fig. 4B PCNA.tif]

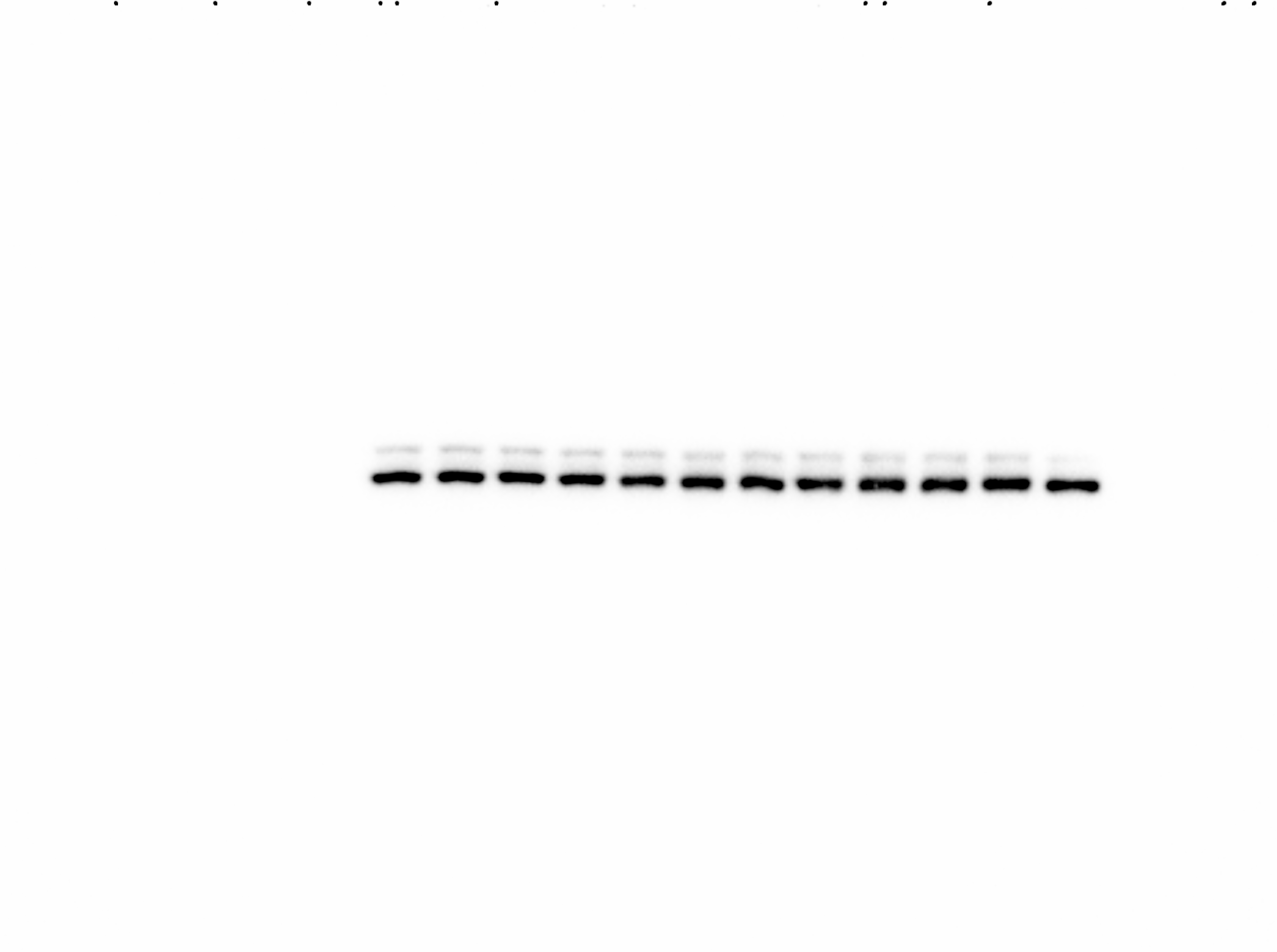

Supplement: Figure 4—source data 2. [file elife-68843-fig4-data2.zip › Figure 4G-Original WB images/Fig. 4G ERK.tif]

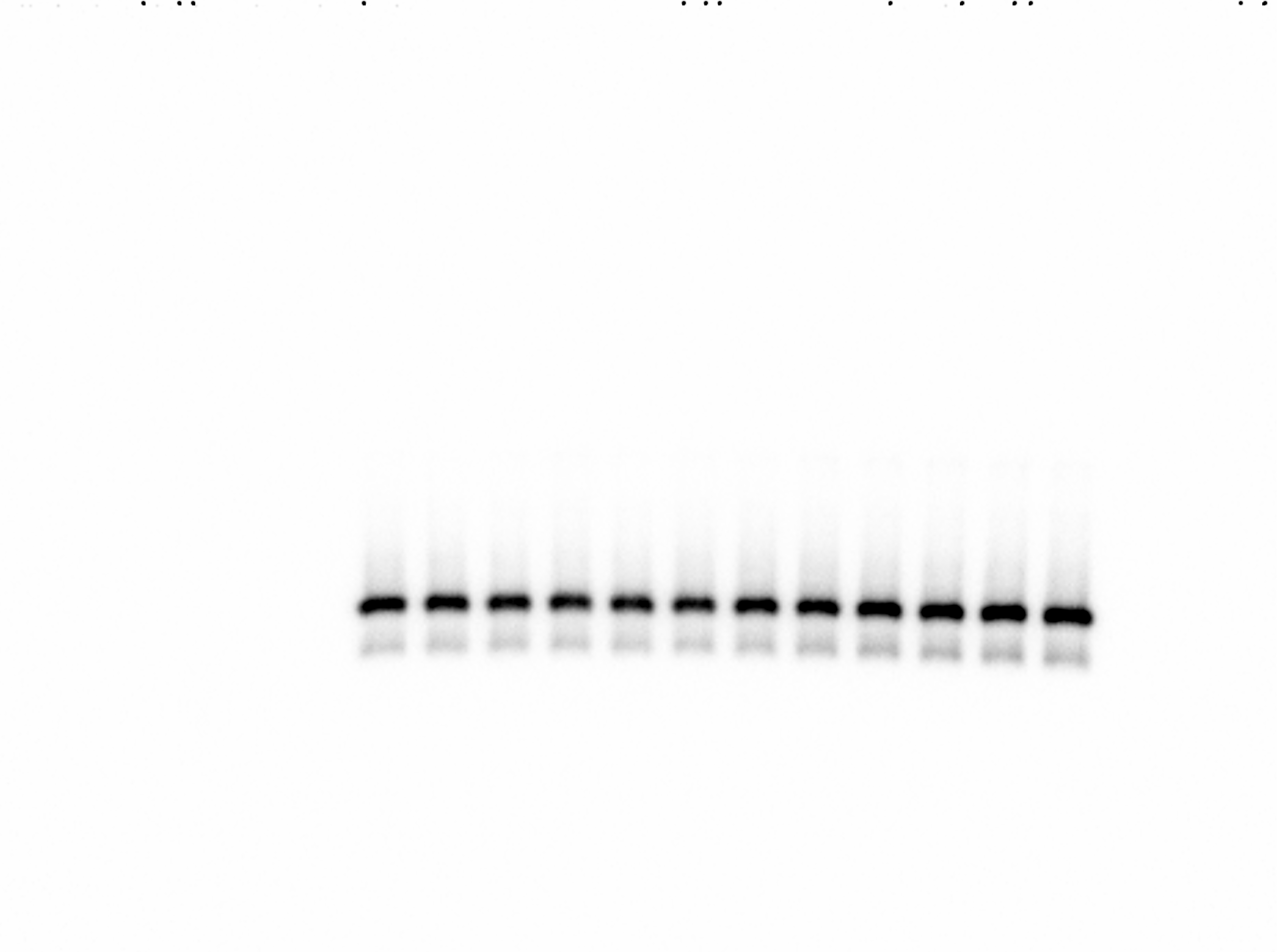

Supplement: Figure 4—source data 2. [file elife-68843-fig4-data2.zip › Figure 4G-Original WB images/Fig. 4G JNK.tif]

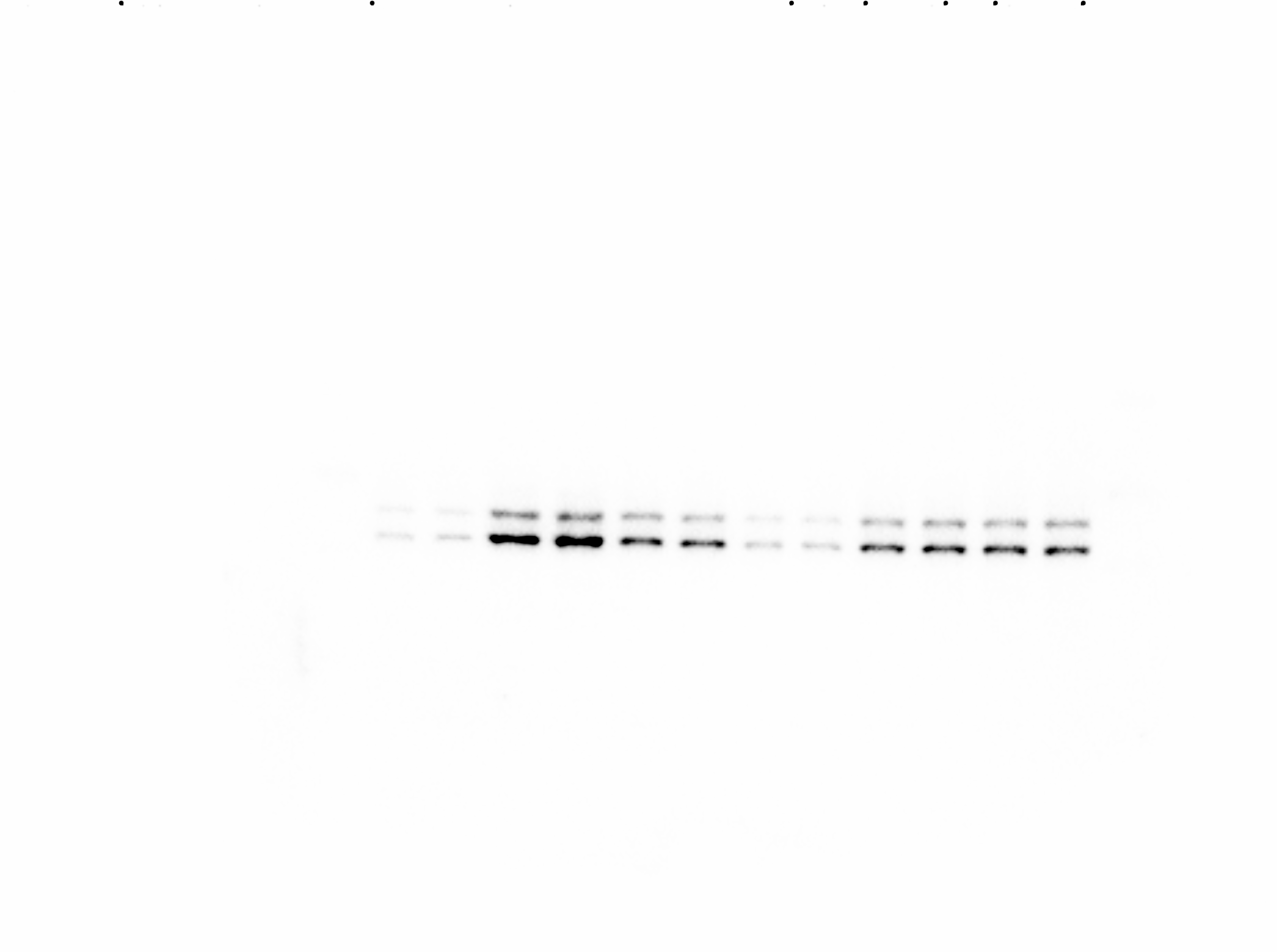

Supplement: Figure 4—source data 2. [file elife-68843-fig4-data2.zip › Figure 4G-Original WB images/Fig. 4G p-ERK.tif]

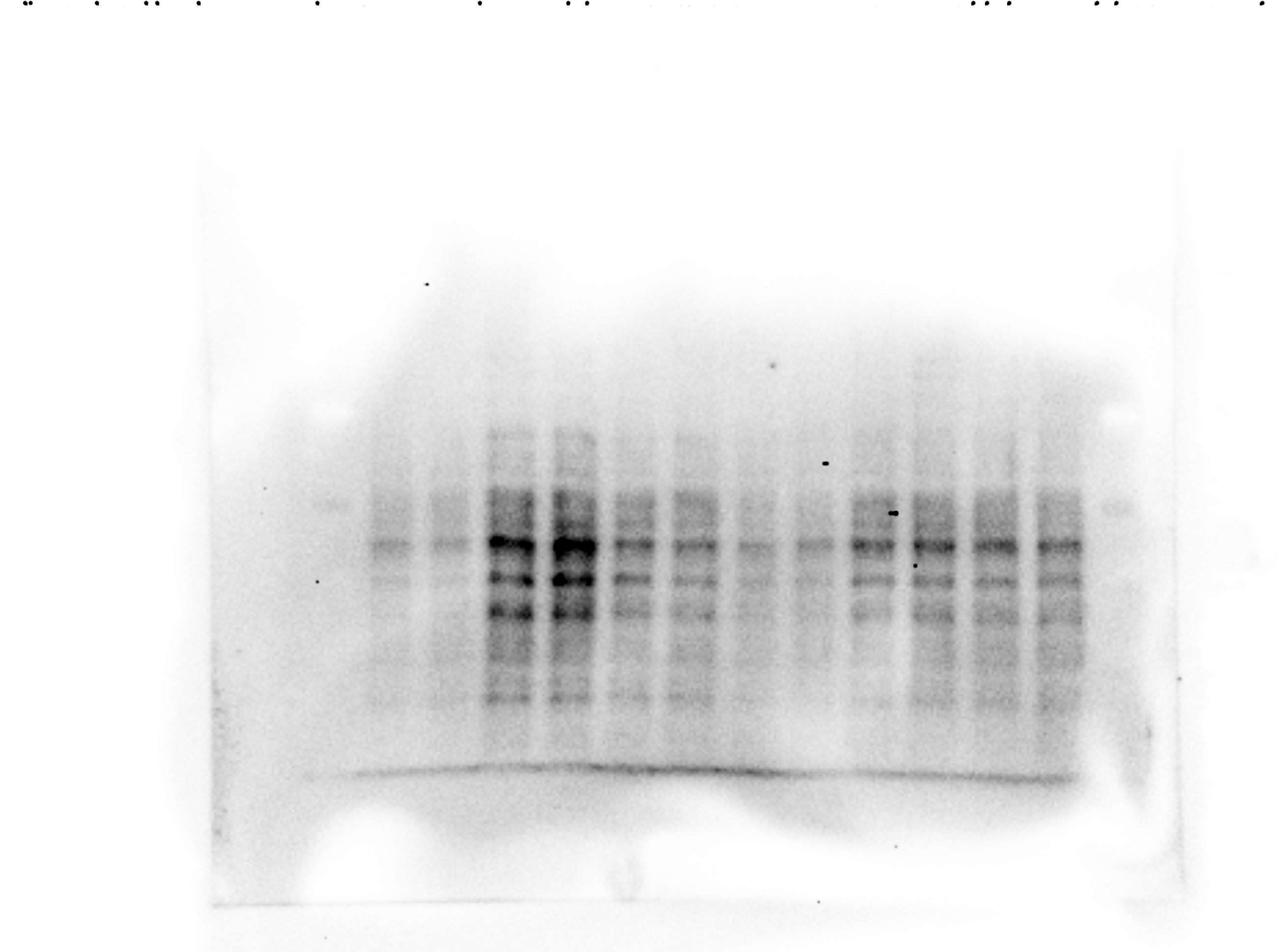

Supplement: Figure 4—source data 2. [file elife-68843-fig4-data2.zip › Figure 4G-Original WB images/Fig. 4G p-JNK.tif]

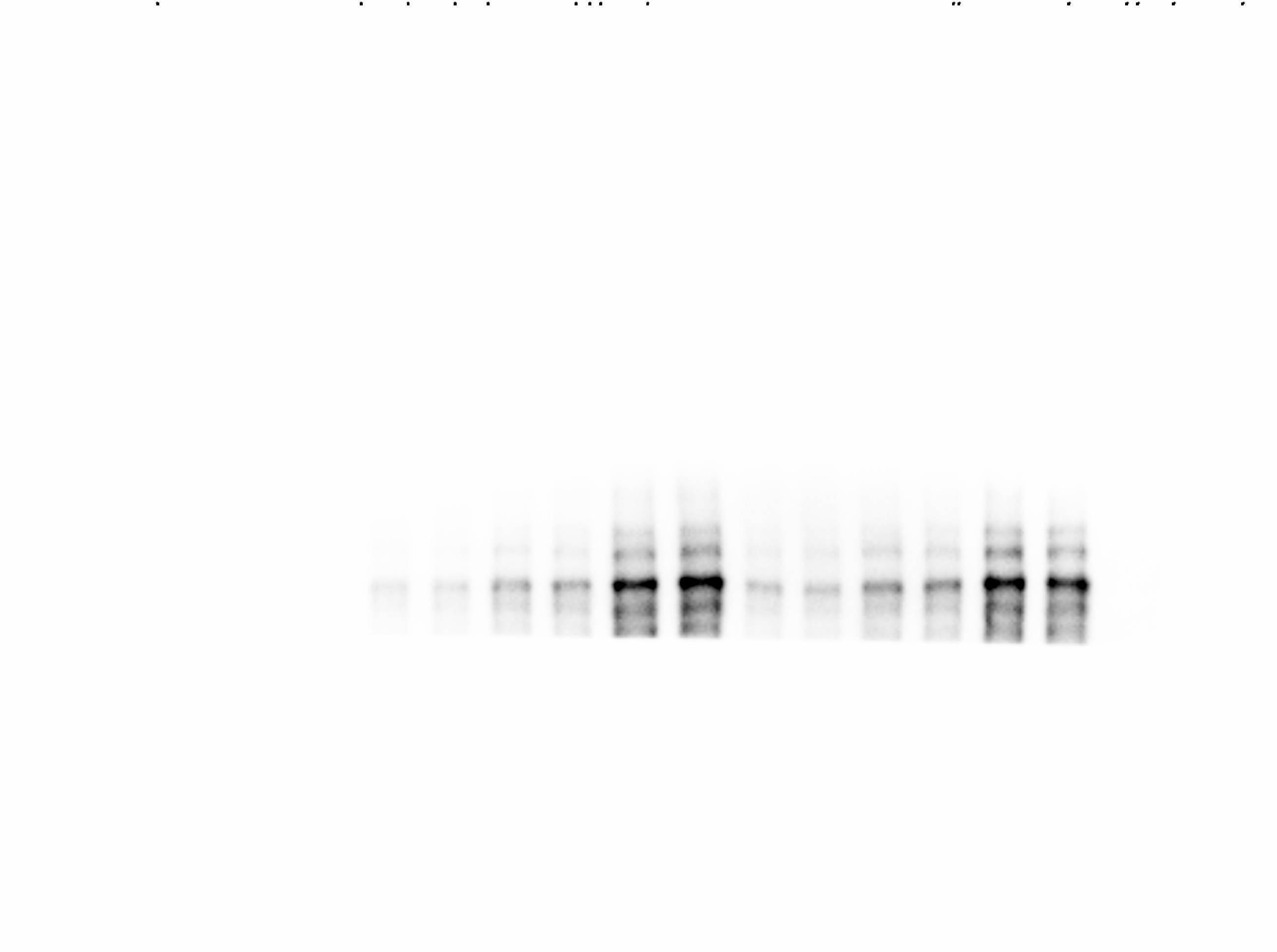

Supplement: Figure 4—source data 2. [file elife-68843-fig4-data2.zip › Figure 4G-Original WB images/Fig. 4G p-STAT3.tif]

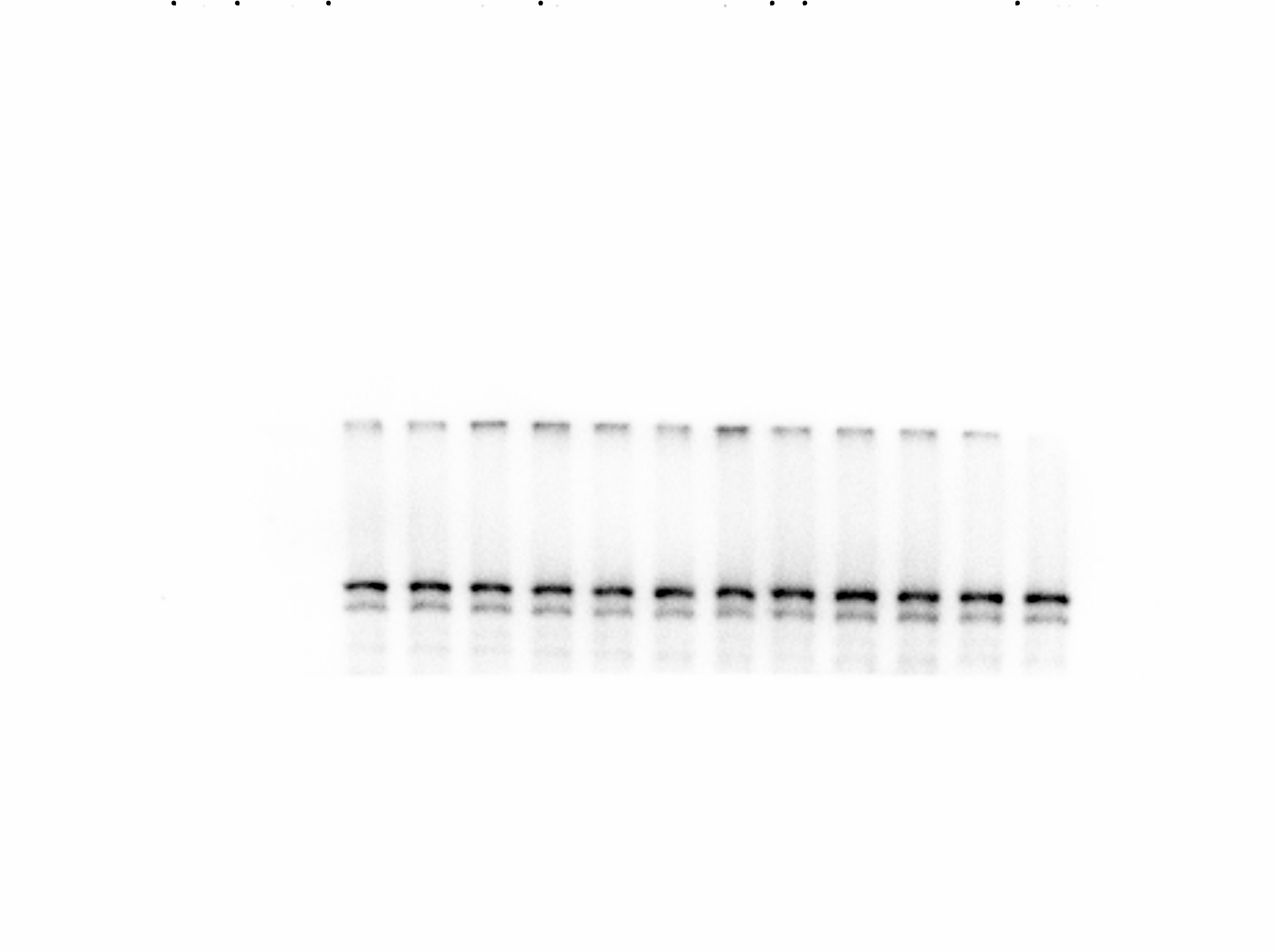

Supplement: Figure 4—source data 2. [file elife-68843-fig4-data2.zip › Figure 4G-Original WB images/Fig. 4G STAT3.tif]
