# Supplementary material for: The pro-regenerative effects of hyperIL6 in drug-induced liver injury are unexpectedly due to competitive inhibition of IL11 signaling
Source: eLife. 2021 Aug 26;10:e68843. doi: 10.7554/eLife.68843 (PMC8445623; doi:10.7554/eLife.68843)
Supplement: Figure 1—figure supplement 1—source data 2. [file elife-68843-fig1-figsupp1-data2.zip › Figure 1-figure supplement 1-Uncropped WB images with markers.pptx]

## Slide 1
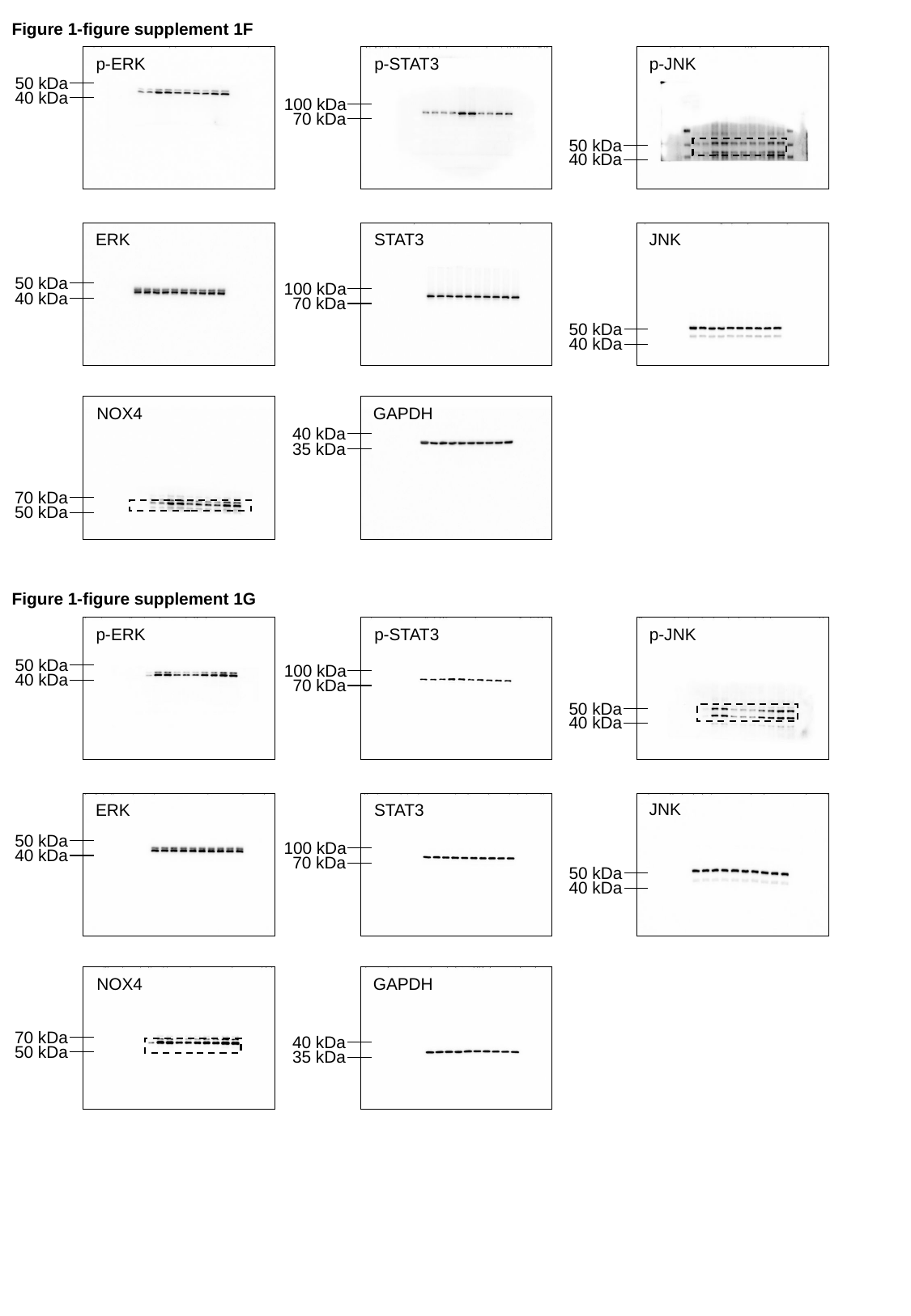

Figure 1-figure supplement 1F
p-JNK
p-ERK
p-STAT3
50 kDa
40 kDa
100 kDa
70 kDa
50 kDa
40 kDa
JNK
ERK
STAT3
50 kDa
40 kDa
100 kDa
70 kDa
50 kDa
40 kDa
GAPDH
NOX4
40 kDa
35 kDa
70 kDa
50 kDa
Figure 1-figure supplement 1G
p-JNK
p-ERK
p-STAT3
50 kDa
40 kDa
100 kDa
70 kDa
50 kDa
40 kDa
JNK
ERK
STAT3
50 kDa
40 kDa
100 kDa
70 kDa
50 kDa
40 kDa
GAPDH
NOX4
70 kDa
50 kDa
40 kDa
35 kDa
