# Supplementary material for: The pro-regenerative effects of hyperIL6 in drug-induced liver injury are unexpectedly due to competitive inhibition of IL11 signaling
Source: eLife. 2021 Aug 26;10:e68843. doi: 10.7554/eLife.68843 (PMC8445623; doi:10.7554/eLife.68843)
Supplement: Figure 2—source data 2. [file elife-68843-fig2-data2.zip › Figure 2-Uncropped WB images with markers.pptx]

## Slide 1
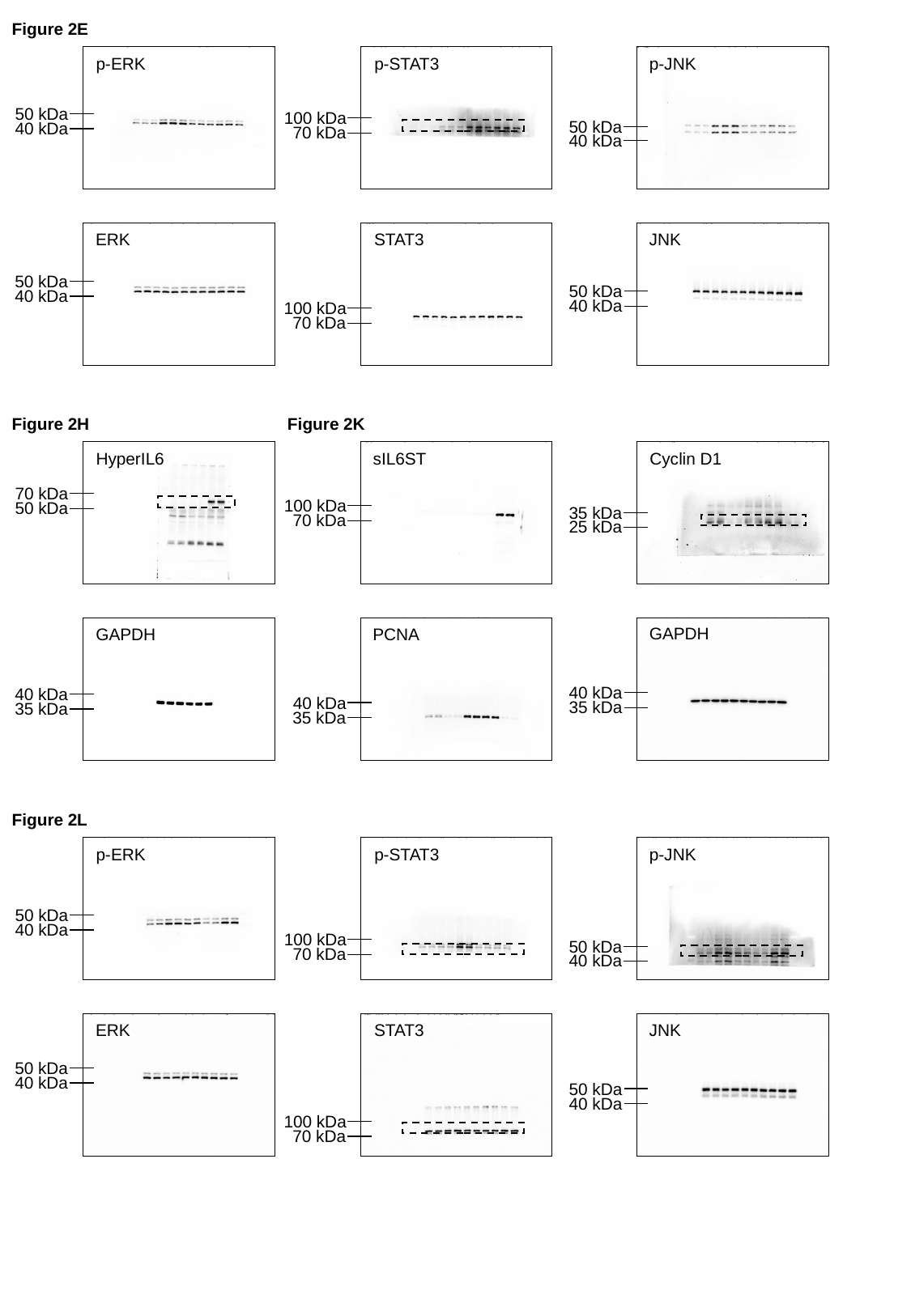

Figure 2E
p-JNK
p-ERK
p-STAT3
50 kDa
40 kDa
100 kDa
70 kDa
50 kDa
40 kDa
JNK
ERK
STAT3
50 kDa
40 kDa
50 kDa
40 kDa
100 kDa
70 kDa
Figure 2H
HyperIL6
70 kDa
50 kDa
GAPDH
40 kDa
35 kDa
Figure 2K
Cyclin D1
sIL6ST
100 kDa
70 kDa
35 kDa
25 kDa
GAPDH
PCNA
40 kDa
35 kDa
40 kDa
35 kDa
Figure 2L
p-JNK
p-ERK
p-STAT3
50 kDa
40 kDa
100 kDa
70 kDa
50 kDa
40 kDa
JNK
ERK
STAT3
50 kDa
40 kDa
50 kDa
40 kDa
100 kDa
70 kDa
