# Supplementary material for: The pro-regenerative effects of hyperIL6 in drug-induced liver injury are unexpectedly due to competitive inhibition of IL11 signaling
Source: eLife. 2021 Aug 26;10:e68843. doi: 10.7554/eLife.68843 (PMC8445623; doi:10.7554/eLife.68843)
Supplement: Figure 3—source data 2. [file elife-68843-fig3-data2.zip › Figure 3-Uncropped WB images with markers.pptx]

## Slide 1
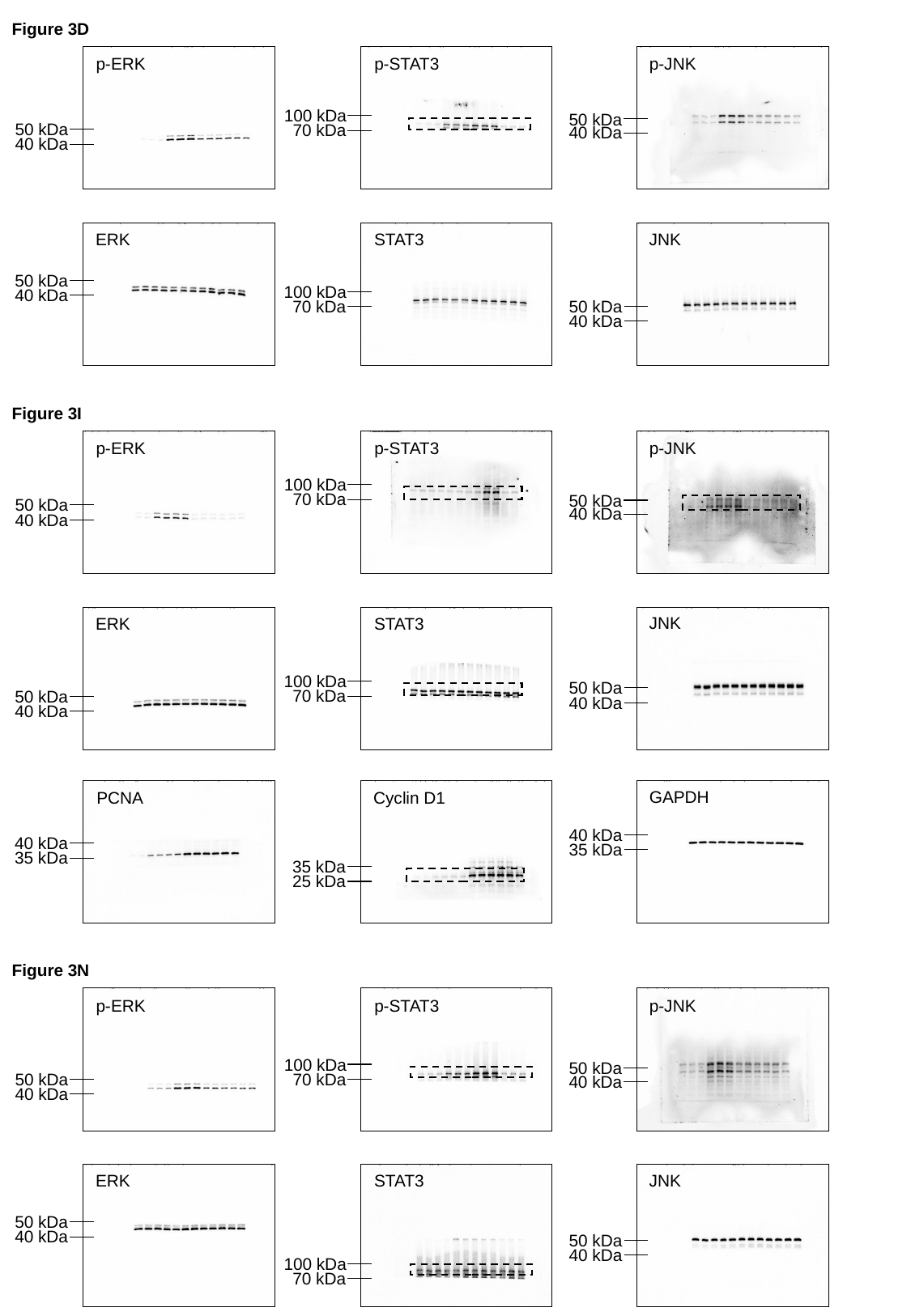

Figure 3D
p-JNK
p-ERK
p-STAT3
100 kDa
70 kDa
50 kDa
40 kDa
50 kDa
40 kDa
JNK
ERK
STAT3
50 kDa
40 kDa
100 kDa
70 kDa
50 kDa
40 kDa
Figure 3I
p-JNK
p-ERK
p-STAT3
100 kDa
70 kDa
50 kDa
40 kDa
50 kDa
40 kDa
JNK
ERK
STAT3
100 kDa
70 kDa
50 kDa
40 kDa
50 kDa
40 kDa
GAPDH
Cyclin D1
PCNA
40 kDa
35 kDa
40 kDa
35 kDa
35 kDa
25 kDa
Figure 3N
p-JNK
p-ERK
p-STAT3
100 kDa
70 kDa
50 kDa
40 kDa
50 kDa
40 kDa
JNK
ERK
STAT3
50 kDa
40 kDa
50 kDa
40 kDa
100 kDa
70 kDa
