# Supplementary material for: The pro-regenerative effects of hyperIL6 in drug-induced liver injury are unexpectedly due to competitive inhibition of IL11 signaling
Source: eLife. 2021 Aug 26;10:e68843. doi: 10.7554/eLife.68843 (PMC8445623; doi:10.7554/eLife.68843)
Supplement: Figure 4—source data 2. [file elife-68843-fig4-data2.zip › Figure 4-Uncropped WB images with markers.pptx]

## Slide 1
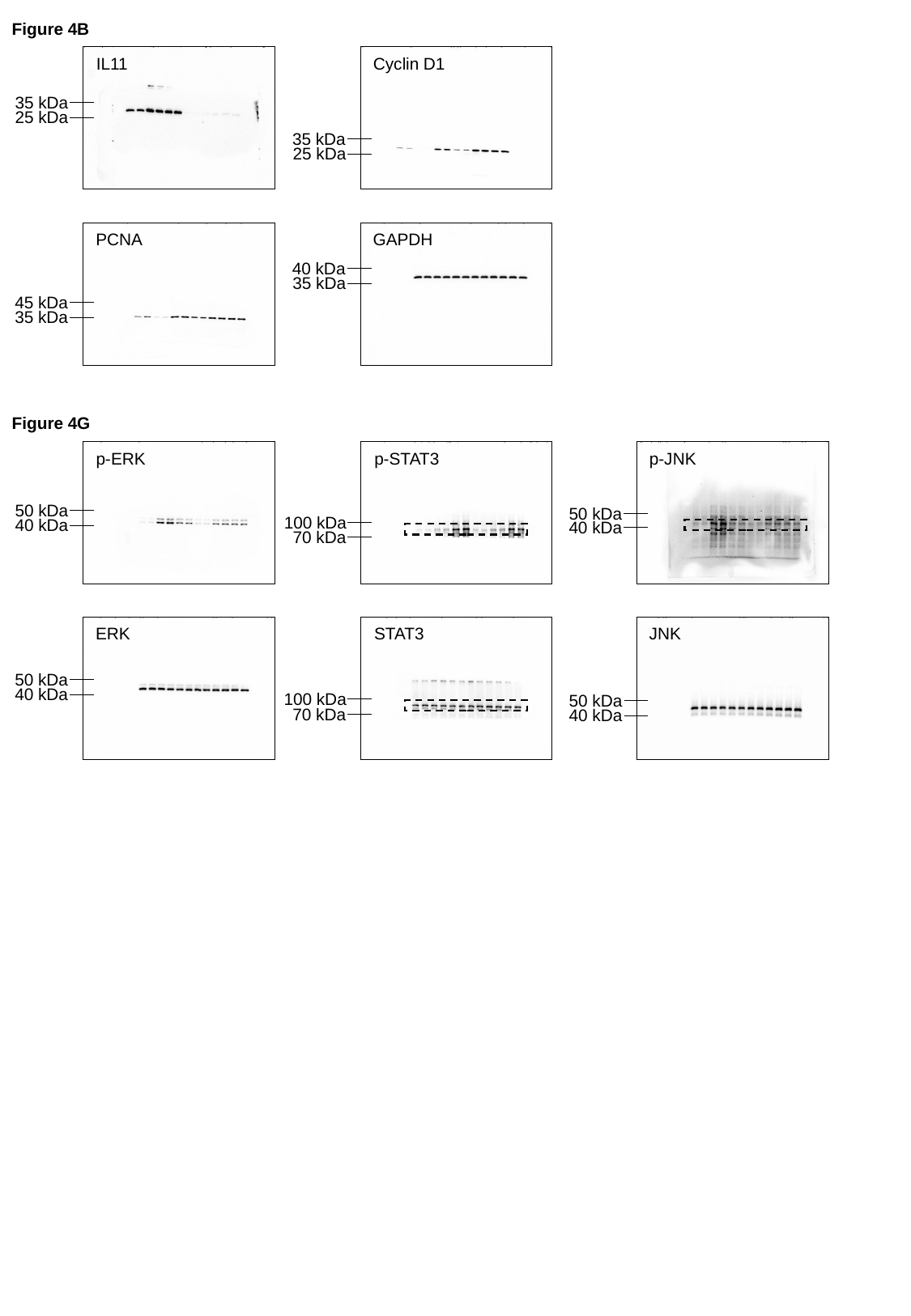

Figure 4B
IL11
Cyclin D1
35 kDa
25 kDa
35 kDa
25 kDa
PCNA
GAPDH
40 kDa
35 kDa
45 kDa
35 kDa
Figure 4G
p-JNK
p-ERK
p-STAT3
50 kDa
40 kDa
50 kDa
40 kDa
100 kDa
70 kDa
JNK
ERK
STAT3
50 kDa
40 kDa
100 kDa
70 kDa
50 kDa
40 kDa
