# Supplementary material for: The pro-regenerative effects of hyperIL6 in drug-induced liver injury are unexpectedly due to competitive inhibition of IL11 signaling
Source: eLife. 2021 Aug 26;10:e68843. doi: 10.7554/eLife.68843 (PMC8445623; doi:10.7554/eLife.68843)
Supplement: Transparent reporting form [file elife-68843-transrepform1.docx]

***eLife’s* transparent reporting form**

We encourage authors to provide detailed information *within their submission* to facilitate the interpretation and replication of experiments. Authors can upload supporting documentation to indicate the use of appropriate reporting guidelines for health-related research (see [EQUATOR Network](http://www.equator-network.org/%20)), life science research (see the [BioSharing Information Resource](https://biosharing.org/)), or the [ARRIVE guidelines](http://www.plosbiology.org/article/info:doi/10.1371/journal.pbio.1000412) for reporting work involving animal research. Where applicable, authors should refer to any relevant reporting standards documents in this form.

If you have any questions, please consult our Journal Policies and/or contact us: [editorial@elifesciences.org](mailto:editorial@elifesciences.org).

**Sample-size estimation**

- You should state whether an appropriate sample size was computed when the study was being designed
- You should state the statistical method of sample size computation and any required assumptions
- If no explicit power analysis was used, you should describe how you decided what sample (replicate) size (number) to use

Please outline where this information can be found within the submission (e.g., sections or figure legends), or explain why this information doesn’t apply to your submission:

The sample size for each experiment was provided in the relevant figure legends. Sample size was decided based on the experience of the authors with molecular and in vivo studies as published in many studies. Sample sizes may vary depending on animal availability.

**Replicates**

- You should report how often each experiment was performed
- You should include a definition of biological versus technical replication
- The data obtained should be provided and sufficient information should be provided to indicate the number of independent biological and/or technical replicates
- If you encountered any outliers, you should describe how these were handled
- Criteria for exclusion/inclusion of data should be clearly stated
- High-throughput sequence data should be uploaded before submission, with a private link for reviewers provided (these are available from both GEO and ArrayExpress)

Please outline where this information can be found within the submission (e.g., sections or figure legends), or explain why this information doesn’t apply to your submission:

The numbers of replicates used for each experiment were described in the relevant figure legends. No outliers were identified in this study, and all data were included in the analyses. No high-throughput sequence data was obtained in this study.

**Statistical reporting**

- Statistical analysis methods should be described and justified
- Raw data should be presented in figures whenever informative to do so (typically when N per group is less than 10)
- For each experiment, you should identify the statistical tests used, exact values of N, definitions of center, methods of multiple test correction, and dispersion and precision measures (e.g., mean, median, SD, SEM, confidence intervals; and, for the major substantive results, a measure of effect size (e.g., Pearson's r, Cohen's d)
- Report exact p-values wherever possible alongside the summary statistics and 95% confidence intervals. These should be reported for all key questions and not only when the p-value is less than 0.05.

Please outline where this information can be found within the submission (e.g., sections or figure legends), or explain why this information doesn’t apply to your submission:

The statistical tests used were described in the relevant Materials and Methods sections. All raw data points were shown in the figures. The statistical tests used, exact values of N, definitions of center, methods of multiple test correction, and dispersion and precision measures were provided in the relevant figure legends. Exact p-values were provided in the relevant figures.

(For large datasets, or papers with a very large number of statistical tests, you may upload a single table file with tests, Ns, etc., with reference to sections in the manuscript.)

**Group allocation**

- Indicate how samples were allocated into experimental groups (in the case of clinical studies, please specify allocation to treatment method); if randomization was used, please also state if restricted randomization was applied
- Indicate if masking was used during group allocation, data collection and/or data analysis

Please outline where this information can be found within the submission (e.g., sections or figure legends), or explain why this information doesn’t apply to your submission:

For *in vitro* studies, the cells from each cell line required for all tested conditions were pooled, equal number of cell were then seeded and stimulated/treated randomly. For *in vivo* studies, the animal were randomly allocated to experimental groups to ensure similar age across groups. Masking was not utilized.

**Additional data files (“source data”)**

- We encourage you to upload relevant additional data files, such as numerical data that are represented as a graph in a figure, or as a summary table
- Where provided, these should be in the most useful format, and they can be uploaded as “Source data” files linked to a main figure or table
- Include model definition files including the full list of parameters used
- Include code used for data analysis (e.g., R, MatLab)
- Avoid stating that data files are “available upon request”

Please indicate the figures or tables for which source data files have been provided:

Source data file has been provided for Figure 1A-G, I-J; Figure 2B-F, H-N; Figure 3B-E, G-K, M-N; Figure 4B-G; Figure 1-figure supplement 1A-H; Figure 2-figure supplement 1; Figure 3-figure supplement 1A-C; Figure 4-figure supplement 1.
